# Disease predisposition of human leukocyte antigen class II genes influences the gut microbiota composition in patients with primary biliary cholangitis

**DOI:** 10.3389/fimmu.2022.984697

**Published:** 2022-09-20

**Authors:** Chun-Yang Huang, Hai-Ping Zhang, Wei-Jia Han, Dan-Tong Zhao, Hui-Yu Liao, Yin-Xue Ma, Bin Xu, Li-Juan Li, Ying Han, Xiu-Hong Liu, Qi Wang, Jin-Li Lou, Xiao-Dan Zhang, Juan Zhao, Wen-Juan Li, Yan-Min Liu, Hui-Ping Yan

**Affiliations:** ^1^ Second Department of Liver Disease Center, Beijing YouAn Hospital, Capital Medical University, Beijing, China; ^2^ Clinical Laboratory Center and Clinical Research Center for Autoimmune Liver Disease, Beijing YouAn Hospital, Capital Medical University, Beijing, China; ^3^ Department of Gastroenterology, Integrated Clinical Microecology Center, Shenzhen Hospital, Southern Medical University, Shenzhen, China; ^4^ Institute of Hepatology, Beijing YouAn Hospital, Capital Medical University, Beijing, China

**Keywords:** primary biliary cholangitis, human leukocyte antigen, gut microbiota, susceptibility gene, bioinformatic analysis

## Abstract

**Background:**

The human leukocyte antigen (HLA) susceptibility gene is the main genetic risk factor for primary biliary cholangitis (PBC). The prognosis of patients with PBC is linked to gut microbiota dysbiosis. However, whether the HLA alleles are associated with the gut microbiota distribution and disease severity remains unknown.

**Methods:**

A cohort of 964 Chinese patients with PBC was enrolled at Beijing YouAn Hospital, Beijing, China. High-resolution genotyping of the HLA class I and class II loci from 151 of these patients was performed using sequence-based PCR. Stool samples were collected from 43 of the 151 fully HLA-typed patients to analyze their microbiota compositions *via* 16S RNA gene sequencing.

**Results:**

Of the 964 patients, the male:female ratio was 114:850, and 342 of these patients (35.5%) had already developed liver cirrhosis (LC) before enrollment. Patients with PBC showed a significantly higher frequency of HLA DRB1*08:03 than did the controls (21.2% vs. 9.0%, *P*=0.0001). HLA-DRB1*03:01, DRB1*07:01, DRB1*14:05, and DRB1*14:54 frequencies were also increased but did not reach significance after Bonferroni’s correction. Conversely, the DQB1*03:01 frequency was significantly lower in patients with PBC than in the controls (24.5% vs. 39.2%, *P*=0.0010). The patients’ gut microbiota were analyzed from four perspectives. The microbial community abundances were significantly lower in FHRAC-positive patients (patients with a combination of five HLA *DRB1* high-risk alleles) than in FHRAC-negative patients (*P*<0.05). Of the top 10 microbial genera, *Lachnospiraceae_incertae_sedis* was higher in the FHRAC-positive patients than in the FHRAC-negative patients (*P*<0.05). linear discriminant analysis (LDA) effect-size (LEfSe) analysis showed different microbes at different levels in the FHRAC-negative patients but not in the FHRAC-positive patients. DQB1*03:01-positive patients contained mostly Lactobacillaceae at the family level. A comparison of the FHRAC-positive patients with and without liver cirrhosis showed that the abundances of *Veillonella* were significantly higher in patients with cirrhosis and FHRAC than in those without cirrhosis and are FHRAC-negative.

**Conclusion:**

The HLA class II genes may influence the gut microbiota compositions in patients with PBC. Differential gut microbiota were expressed at different taxonomic levels. Some bacterial abundances may be increased in FHRAC-positive patients with PBC and cirrhosis.

## Introduction

Primary biliary cholangitis (PBC) is an autoimmune liver disease, with its hallmark being the serological signature of the antimitochondrial antibody (AMA) and specific bile duct pathology. The disease is chronic and often progressive, resulting in end-stage liver disease and associated complications. In China, a meta-analysis estimated that the prevalence of PBC was 20.5/100,000 persons (1). In European populations, the estimated incidence is 1–2/100,000 persons annually, and commonly cited ranges for PBC incidence and prevalence are 0.3–5.8 and 1.9–40.2/100,000 persons, respectively (2). Interactions among environmental, genetic/epigenetic, and immunological factors play crucial roles in the PBC pathogenesis, although the factors leading up to disease initiation and progression mechanisms are poorly understood.

Epidemiological studies from different countries have provided genetic evidence based on familial clustering and suggest that PBC results from the combination of “bad genes and bad luck”. In researching the genetic basis of PBC, case-control studies have revealed an association between human leukocyte antigen (HLA) alleles and PBC development. Specifically, members of the DRB1*08 allele family, including DRB1*08:01, DRB1*08:03, DRB1*14, and DPB1*03:01, were identified as susceptible alleles, and DRB1*11 and DRB1*13 were identified as protective alleles (3). HLA-DRB1*08:03, HLA-DRB1*07:01, and DPB1*17:01 were identified as the major susceptible alleles, and DQB1*03:01 and the DRB1*12:02-DQB1*03:01 haplotype were significantly decreased in a different cohort of Chinese patients (4, 5).

The gut microbiota has been proposed as a potential environmental component of PBC because it influences patients’ treatment and prognosis. Li et al. suggested that microbiota alterations were closely related to beneficial responses to PBC, highlighting the possibility of exploring bile acid–microbiota interactions for treating patients (6). Lammert et al. reported that microbiota alterations might correspond to liver fibrosis progression (7). Previous studies have also suggested changes in microbiota compositions in autoimmune diseases, such as rheumatoid arthritis, type 1 diabetes, and celiac disease, which are at least partially due to the effects of susceptible HLAs (8, 9). We previously reported that a distinct gut microbiome profile was associated with PBC prognosis (10). However, whether the HLA alleles influence the gut microbiota distribution, and whether a link exists between disease development, severity, and gut microbiota alterations in patients with PBC remain unknown.

In the present study, using the PBC cohort we established during the past decade, we analyzed the HLA genotyping and gut microbiota in a proportion of these well-defined patients. The results showed associations between HLA alleles, microbiota compositions, and functional characteristics.

## Methods

### Patients and samples

A cohort of 964 Chinese patients with PBC was enrolled from 2010 to 2020 in Beijing YouAn Hospital, Beijing, China. The average follow-up time was 40.1 ± 27.5 months; the longest was 107.6 months. The male:female ratio was 114:850, and the average age was 56.5 ± 10.9 years. Of the 964 patients, 151 underwent HLA genotyping. Forty-three of the 151 fully HLA-typed patients were enrolled, and their stool samples were analyzed. The stool samples were collected from April 2019 to December 2021 and stored at −80°C. The patients’ gut microbiota were analyzed from the samples shortly after collection.

Inclusion criteria were that (1) all patients had a definite diagnosis of PBC based on the European Association for the Study of the Liver Clinical Practice Guidelines of PBC (2); (2) all were aged >18 years; and (3) all were of Chinese nationality, with most being from northern China. Exclusion criteria were (1) liver diseases caused by hepatitis viruses or drugs, alcoholic and non-alcoholic fatty liver disease; (2) hepatocellular carcinoma or liver metastases; (3) severe cardiac or renal insufficiency; (4) intestinal diseases or intestinal surgery; and (5) antibiotics or intestinal microecologics used within 2 weeks prior. Beijing YouAn Hospital (Ethics: No. [2012] 44 and No. LL-2018-044-K) approved this study. All enrolled patients signed their informed consent.

### Serum biochemical and immunologic parameters

Routine laboratory tests were performed for alanine transaminase (ALT), aspartate transaminase (AST), total bilirubin (TBIL), alkaline phosphatase (ALP), gamma-glutamyltransferase (GGT), albumin (ALB), total bile acid (TBA), creatinine (Cr), white blood cells (WBCs), hemoglobin (HB), and platelets (PLTs). AMA, antinuclear antibody (ANA), anti-gp210, and anti-sp100 were detected by indirect immunofluorescence and immunoblotting assays (EUROIMMUN, Lübeck, Germany), and immunoglobulin (Ig)M and IgG were included as serum parameters.

### DNA extraction and HLA genotyping

Genomic DNA was purified from peripheral whole blood using the hydrochloride method as described previously (4, 11). High-resolution genotyping of the HLA class I (A, B, C) and class II (DRB1 and DQB1) loci was performed using sequence-based PCR (Beijing Genomics Institute-Shenzhen [BGI-Shenzhen]; Shenzhen, China). Data from 500 healthy people were used as controls (4).

### Stool analysis of the 16s RNA gene

Microbial community DNA was extracted using a MagPure Stool DNA KF kit B (Magen, Guangzhou, China) following the manufacturer’s instructions. DNA was quantified with a Qubit Fluorometer using a Qubit^®^ dsDNA BR Assay kit (Invitrogen, California, USA), and the quality was checked by running aliquots on 1% agarose gel.

Variable regions of the V4 region of the bacterial 16S rRNA gene were amplified with degenerate PCR primers: 515F (5′-GTGCCAGCMGCCGCGGTAA-3′) and 806R (5′-GGACTACHVGGGTWTCTAAT-3′). Both forward and reverse primers were tagged with Illumina adapter, a pad, and linker sequences. PCR enrichment was performed in a 50-μl reaction volume containing a 30-ng template, fusion PCR primer, and PCR master mix. PCR cycling conditions were 95°C for 3 min, 30 cycles of 95°C for 45 s, 56°C for 45 s, 72°C for 45 s, and a final extension at 72°C for 10 min. The PCR products were purified using Agencourt AMPure XP beads and eluted in an elution buffer. Libraries were qualified using the Agilent Technologies 2100 bioanalyzer. The validated libraries were used for sequencing on the Illumina platform (HiSeq 2500) following the manufacturer’s standard protocols and generating 2 × 250-bp paired-end reads **(**BGI-Shenzhen; Shenzhen, China).

### Data bioinformatics analysis

High-quality clean data were filtered and retained for analysis. Reads were spliced into tags. Operational taxonomic units (OTUs) with a 97% similarity cutoff were used. Tags were clustered into OTUs *via* USEARCH (v7.0.1090). Species were annotated by comparing OTUs with the GreenGene database (v.201305). From the OTUs and annotation results, α- and β-diversities were calculated using the Wilcoxon and Wilcoxon rank-sum tests, respectively. The differential genera and predicted pathways were screened using the Wilcoxon test (*P* < 0.05) and |log (fold change [FC]) |>1 through PICRUSt2 v2.2.0-b, R (v3.4.10). Differential microbiotas were analyzed through LDA effect size (LEfSe) (https://huttenhower.sph.harvard.edu/galaxy/). At the class level, microbes whose relative abundances were <0.5% in all samples were merged into “Others”. Spearman’s correlation coefficient analysis of the species revealed important patterns and relationships among dominant species **(**BGI-Shenzhen, Shenzhen, China).

### Statistical analysis

Continuous variables are presented as the mean ± standard deviation. All statistical analyses were performed using the SPSS17.0 software, and *P*<0.05 was considered statistically significant.

SPASS 17.0 software was used for the HLA genotyping statistical analysis. Allele distributions were compared between patients and controls, using the chi-square or Fisher’s exact tests. A two-sided *P*<0.05 was considered statistically significant. For multiple testing, *P*-values were corrected (*Pc*) by the number of comparisons according to Bonferroni’s inequality method. Association strengths were estimated by calculating the odds ratio (OR) and 95% confidence interval (CI).

In the gut microbiota analyses, continuous variables are expressed as means and standard deviations. Correlation coefficients ≥0.29 or ≤−0.29 were considered relevant. *P*<0.05 was considered statistically significant. All statistical analyses were performed using an SPSS software (v. 19). α-diversity and β-diversity analyses and partial least-squares-discriminant analysis (PLS-DA) were performed using R (v.3.2.1).

## Results

### Baseline characteristics of the 964 patients with PBC

**Figure 1:**
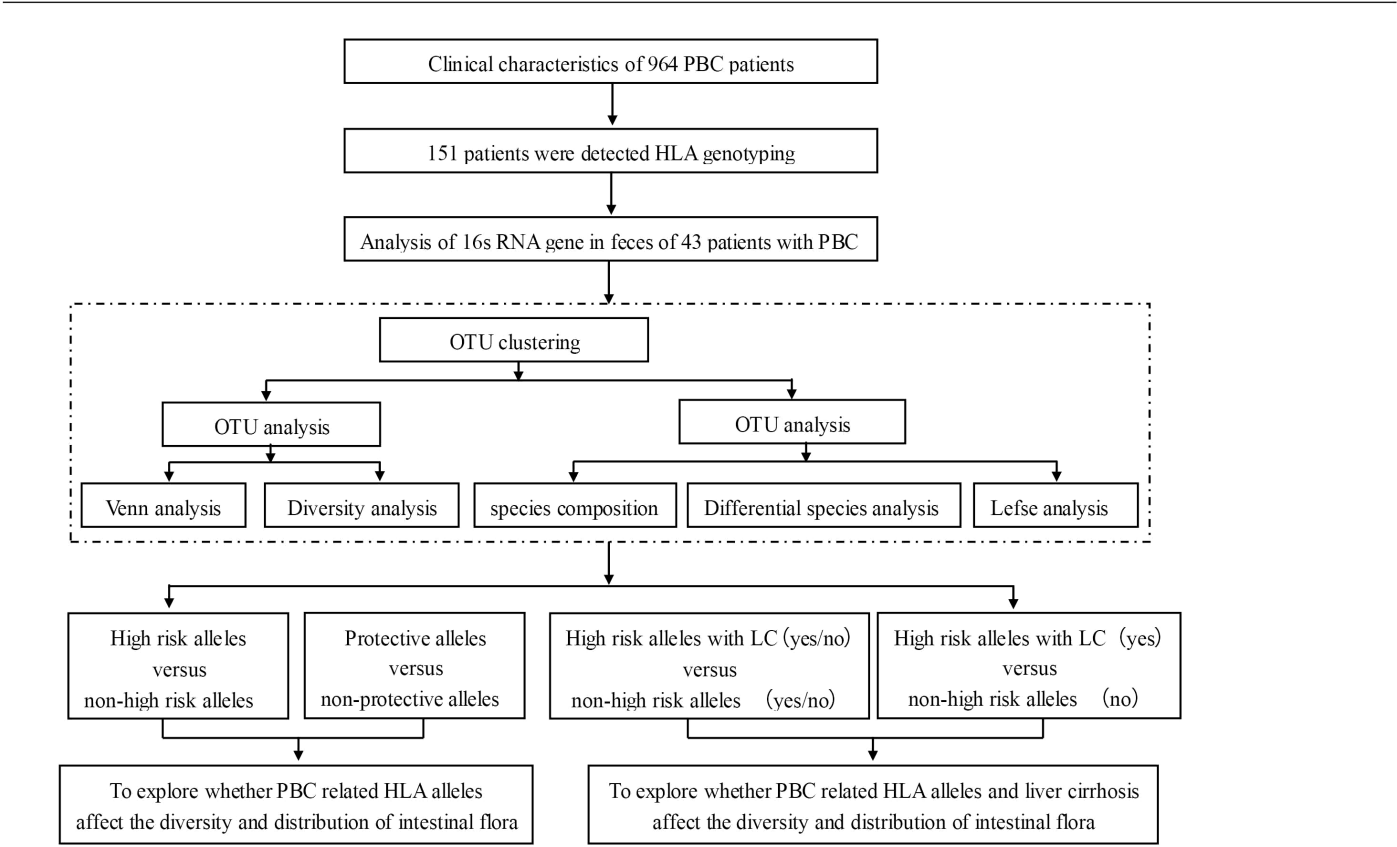
The study flowchart.

Of the patients in our cohort, 60.6% (584/964) were enrolled at initial diagnosis, 39.4% (380/964) were enrolled while undergoing treatment, 73.5% (709/964) had elevated serum ALP, and 55.9% (514/920) had elevated serum IgM (baseline results of IgM were absent from 44 patients). The specific autoantibodies AMA (or AMA-M2), anti-gp210, and anti-sp100 were detected in 92.7%, 28.2%, and 12.7% of patients, respectively. Of the 964 patients, 342 (35.5%) had developed liver cirrhosis (LC) prior to enrollment. From this cohort, we previously reported the HLA alleles and their associations with ANA (4), gut microbiota alterations and elevated serum bilirubin (10), and the clinical characteristics of the patients with anti-gp210 (12). Here, we further explored the association between the HLA alleles and the microbiota in these patients (**Figure 1**).

### HLA allele distribution

Patients with PBC showed a significantly higher frequency of HLA DRB1*08:03 than did the controls (21.2% vs. 9.0%, *P*=0.0001/*Pc*=0.0063, OR= 2.71, 95% CI:1.59–4.58). HLA-DRB1*03:01(13.1% vs. 7.8%, *P*=0.049), DRB1*07:01 (29.1% vs. 20.2%, *P*=0.0254), DRB1*14:05 (8.3% vs. 4.2%, *P*=0.05), DRB1*14:54 (9.9% vs. 5.2%, *P*=0.05), and DQB1*06:01 (25.8% vs. 17.8%, *P*=0.0353) frequencies were increased in patients with PBC, but did not reach significance after Bonferroni’s correction. The HLA DQB1*03:01 frequency was significantly lower in patients with PBC than in the controls (24.5% vs. 39.2%, *P*=0.0010/*Pc*=0.0153, OR=0.50, 95% CI:0.32–0.77). DRB1*11:01 (5.3% vs. 10.6%, *P*=0.05) and DRB1*12:02 (7.3% vs. 14.8%, *P*=0.0185) were less common in patients with PBC than in the controls.

### Gut microbiota analysis in patients with PBC with different HLA alleles

The intestinal flora of 43 patients were analyzed from four perspectives. The first was patients who were positive vs. negative for a combination of five HLA DRB1 alleles occurring at high-risk. Of these 43 patients, only seven carried susceptible DRB1*08:03, a combination group of these five high-risk HLA *DRB1* alleles: DRB1*03:01 (5/43), DRB1*07:01 (9/43), DRB1*08:03 (7/43), DRB1*14:05 (4/43), and DRB1*14:54 (4/43) was set up (abbreviation: Group FHRAC). Of the 43 patients, 25 were positive for any of the five alleles, and 18 were negative for all five alleles. We further analyzed patients who were positive vs. negative for the “protective” allele DQB1*03:01, patients who were FHRAC-positive with and without LC, and patients who were FHRAC-positive with LC vs. patients who were FHRAC-negative without LC.

### Gut microbiota in patients with vs. without high-risk HLA alleles

#### (1) Baseline characteristics

FHRAC-positive (n=25) and FHRAC-negative (n=18) patients were classified according to their conditions. The mean sex, age, body mass index (BMI), ALT, AST, TBIL, ALP, GGT, ALB, TBA, Cr, WBCs, HB, PLT, anti-sp100, IgM, and IgG did not significantly differ between the two groups. However, anti-gp210 occurred more frequently in FHRAC-negative than in FHRAC-positive patients (**Table 1**).

**Table 1:** The baseline characteristics of PBC in FHRAC-positive and FHRAC-negative groups.

Variables	FHRAC-positive ( n = 25 )	FHRAC-negative ( n = 18 )	*P* -value
Gender (female/male)	22 (91.67%)/3 (8.33%)	17 (94.4%)/1 (5.6%)	0.473
Age (years)	54.09 ± 10.35	54.63 ± 9.53	0.862
γ-GT (U/L)	263.68 ± 322.73	297.34 ± 243.70	0.712
ALB (g/L)	40.36 ± 7.64	39.19 ± 6.76	0.605
ALT (U/L)	70.14 ± 73.68	63.07 ± 49.94	0.726
ALP (U/L)	316.90 ± 258.68	277.81 ± 123.39	0.514
AST (U/L)	90.59 ± 86.14	79.11 ± 46.26	0.610
GLO (g/L)	37.74 ± 8.70	36.72 ± 7.36	0.689
TBIL (μmol/L)	30.62 ± 23.43	43.27 ± 41.00	0.207
TBA (μmol/L)	37.15 ± 40.51	58.79 ± 49.85	0.124
Cr (μmol/L)	47.46 ± 10.53	48.94 ± 12.08	0.678
WBC (*10^9/L)	4.66 ± 1.45	4.60 ± 2.38	0.915
HB (g/L)	121.76 ± 20.01	112.67 ± 14.66	0.110
PLT (*10^9^/L)	185.56 ± 79.24	171.83 ± 97.85	0.614
IgA (g/L)	3.79 ± 1.87	3.69 ± 1.65	0.865
IgG (g/L)	20.43 ± 10.14	17.49 ± 4.54	0.287
IgM (g/L)	4.01 ± 3.79	4.39 ± 2.98	0.738
PT (s)	12.03 ± 1.71	11.34 ± 1.62	0.636
INR	1.09 ± 0.167	1.02 ± 0.146	0.703
Anti-gp210 (neg/pos)	21 (84%)/4 (16%)	8 (44.44%)/10 (55.56%)	0.006*
Anti-sp100 (neg/pos)	20 (80%)/5 (20%)	17 (94.44%)/1 (5.56%)	0.177
Cirrhosis (no/yes)	20 (80%)/5 (20%)	13 (72.22%)/5 (27.78%)	0.406

PBC, primary biliary cholangitis; γ-GT, γ-glutamyl transpeptidase; ALB, albumin; ALT, alanine aminotransferase; ALP, alkaline phosphatase; AST, aspartate aminotransferase; GLO, globulin; TBIL, total bilirubin; TBA, total bile acid; Cr, creatinine; WBC, white blood cell; HB, hemoglobin; PLT, platelet; IgA, immunoglobulin A; IgG, immunoglobulin G; IgM, immunoglobulin M; PT, prothrombin time; INR, international standardized ratio; neg, negative; pos, positive; *P < 0.05.

#### (2) Bacterial richness and diversity analyses

**Figure 2:**
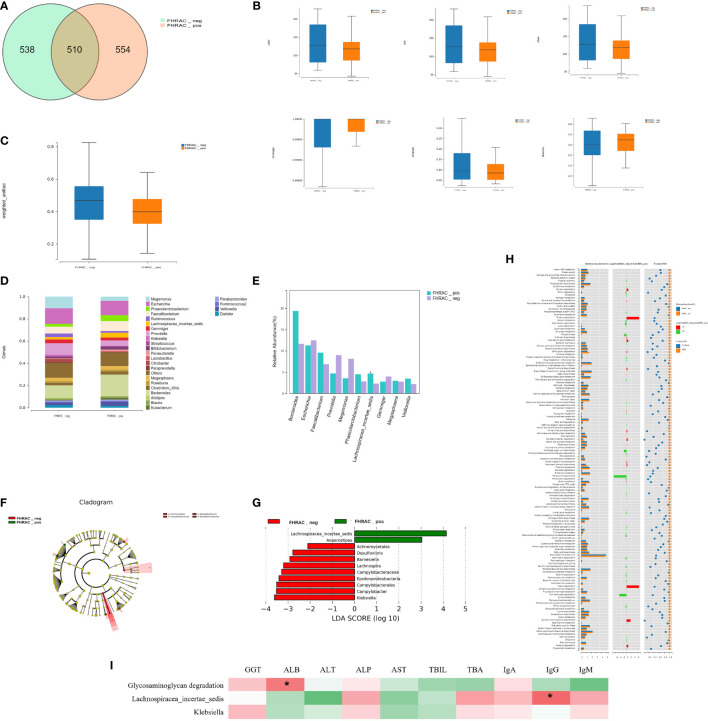
Bacterial richness and diversity analysis. **(A)** We found 510 shared universal OTUs in samples from both FHRAC-positive and FHRAC-negative patients. FHRAC-positive patients contained more unique OTUs than did FHRAC-negative patients (554 vs. 538 OTUs). **(B)** α-diversity analysis showed no significant differences in the Simpson’s, Shannon’s, Sobs, abundance-based coverage estimator (ACE), Chao, or other observed species indexes between the two groups (*P* > 0.05). **(C)** Microbial community diversity in the FHRAC-positive patients was significantly lower than that of the FHRAC-negative patients (*P* < 0.05). **(D)** Twenty-seven microbial genera were detected. In FHRAC-positive patients, *Bacteroides*, *Escherichia*, Others, *Faecalibacterium*, *Phascolarctobacterium*, *Prevotella*, *Lachnospiraceae incertae*, *Megamonas*, *Veillonella*, and *Megasphaera* were the top genera, constituting 79% of the taxa. *Escherichia*, Others, *Bacteroides*, *Megamonas*, *Prevotella*, *Faecalibacterium*, *Gemmiger*, *Ruminococcus*, *Megasphaera* and *Citrobacter* were the top genera in the FHRAC-negative patients (78%). **(E)** The Wilcoxon test was used to compare the significantly different top 10 genera. *Lachnospiraceae_incertae_sedis* was higher in FHRAC-positive patients than in FHRAC-negative patients. **(F)** linear discriminant analysis (LDA) effect-size (LEfSe) analysis showed that FHRAC-negative patient samples contained mainly Epsilonproteobacteria at the class level, Campylobacterales and Actinomycetales at the order level and Campylobacteraceae at the family level. **(G)** *Lachnospiraceae_incertae_sedis*, *Anaerostipes*, *Actinomycetales*, *Desulfovibrio*, *Barnesiella*, *Lachnospira*, *Campylobacteraceae*, *Epsilonproteobacteria*, *Campylobacterales*, *Campylobacter*, and *Klebsiella* were differentially expressed among the taxonomic levels. **(H)** We detected 157 metabolites or pathways at the KEGG level 3. The Wilcoxon test was used to compare the significantly different features. The differentially expressed microbiota were closely related to four differential metabolic pathways, including glycosaminoglycan degradation. **(I)** The Spearman’s correlation coefficient showed an association between clinical manifestations and microbial taxa. Glycosaminoglycan degradation was positively correlated with albumin (ALB). *Lachnospiraceae_incertae_sedis* was positively correlated with IgG. *P<0.05.

Five hundred and ten shared “universal” OTUs were found in both FHRAC-positive and FHRAC-negative patients. FHRAC-positive patients contained more unique OTUs than did FHRAC-negative patients (554 vs. 538; **Figure 2A**).

The α-diversity analysis showed no significant differences on the Simpson’s, Shannon’s, Sobs, abundance-based coverage estimator (ACE), Chao, or other observed species indexes between the two groups (*P*>0.05; **Figure 2B**). However, the microbial community abundance was significantly lower in FHRAC-positive patients than in FHRAC-negative patients (*P*<0.05; **Figure 2C**).

#### (3) Comparison of bacterial compositions

Twenty-seven microorganismal genera were detected. In the FHRAC-positive patients, *Bacteroides*, *Escherichia*, Others, *Faecalibacterium*, *Phascolarctobacterium*, *Prevotella*, *Lachnospiraceae incertae*, *Megamonas*, *Veillonella*, and *Megasphaera* were the top genera, constituting 79% of all genera. *Escherichia*, Others, *Bacteroides*, *Megamonas*, *Prevotella*, *Faecalibacterium*, *Gemmiger*, *Ruminococcus*, *Megasphaera*, and *Citrobacter* were the top genera in the FHRAC-negative patients (78%; **Figure 2D** and **Supplementary Table S1A**). The Wilcoxon test was used to compare the significantly different top 10 genera. *Lachnospiraceae_incertae_sedis* was higher in FHRAC-positive patients than in FHRAC-negative patients (**Figure 2E** and **Supplementary Table S1B**).

LEfSe analysis showed that samples from the FHRAC-negative patients mainly contained Epsilonproteobacteria at the class level, Campylobacterales and Actinomycetales at the order level, and Campylobacteraceae at the family level (**Figure 2F**). *Lachnospiraceae_incertae_sedis*, *Anaerostipes*, *Actinomycetales*, *Desulfovibrio*, *Barnesiella*, *Lachnospira*, *Campylobacteraceae*, *Epsilonproteobacteria*, *Campylobacterales*, *Campylobacter*, and *Klebsiella* were differentially expressed among the taxonomic levels (**Figure 2G**). LEfSe analysis showed no distinguishing characteristics among the FHRAC-positive patients.

#### (4) Association between featured bacterial taxa and pathways

PICRUSt was used to predict the functional pathways based on the 16S rRNA gene to obtain information on metabolites or pathways that may be involved in PBC. We detected 157 metabolites or pathways at the Kyoto Encyclopedia of Genes and Genomes (KEGG) level 3. The Wilcoxon test was used to compare the significantly different features. The differentially expressed microbes were closely related to four differential metabolic pathways, including glycosaminoglycan degradation (**Figure 2H**). The Spearman’s correlation coefficient revealed an association between clinical manifestations and microbial taxa. Glycosaminoglycan degradation was positively correlated with ALB, and *Lachnospiraceae_incertae_sedis* was positively correlated with IgG (**Figure 2I**). In FHRAC-positive patients, *Lachnospiraceae_incertae_sedis* and *Anaerostipes* were significantly decreased, and *Campylobacter*, *Lachnospira*, *Desulfovibrio*, *Klebsiella*, and *Barnesiella* were significantly increased. *Lachnospiraceae_incertae_sedis* may affect liver function in patients with PBC by affecting glycosaminoglycan metabolism.

### Gut microbiota compositions in patients with vs. without the protective allele DQB1*03:01

#### (1) Baseline characteristics

Of 42 patients, 13 were DQB1*03:01-positive, and 29 were DQB1*03:01-negative. The mean sex, age, BMI, ALT, AST, GGT, ALB, Cr, WBCs, HB, PLT, anti-sp100, anti-gp210, TBA, IgM, and IgG did not significantly differ between the two groups. However, TBIL and ALP levels were higher in the DQB1*03:01-negative than in the DQB1*03:01-positive patients (**Table 2**).

**Table 2:** The baseline characteristics of PBC in the DQB1* 03:01-positive and DQB1* 03:01-negative groups.

Variables	DQB1* 03:01-positive ( n = 13 )	DQB1* 03:01-negative ( n = 29 )	*P* -value
Gender (female/male)	12 (92.31%)/1 (7.69%)	26 (89.66%)/3 (10.34%)	0.787
Age (years)	55.73 ± 7.19	54.11 ± 10.88	0.626
γ-GT (U/L)	203.49 ± 186.75	295.96 ± 317.88	0.337
ALB (g/L)	39.26 ± 6.26	40.03 ± 7.81	0.755
ALT (U/L)	60.05 ± 52.33	70.69 ± 70.52	0.630
ALP (U/L)	228.73 ± 108.53	320.47 ± 234.10	0.090
AST (U/L)	68.95 ± 40.86	92.88 ± 82.48	0.329
GLO (g/L)	41.26 ± 7.79	35.84 ± 7.75	0.043
TBIL (μmol/L)	21.47 ± 9.40	42.88 ± 36.86	0.006*
TBA (μmol/L)	33.12 ± 25.64	53.09 ± 51.47	0.102
Cr (μmol/L)	51.85 ± 14.77	46.71 ± 9.10	0.185
WBC (*10^9^/L)	4.85 ± 2.24	4.52 ± 1.75	0.612
HB (g/L)	116.15 ± 11.54	119.10 ± 21.00	0.562
PLT (*10^9^/L)	178.46 ± 95.40	179.93 ± 85.774	0.961
IgA (g/L)	3.82 ± 1.52	3.81 ± 1.859	0.982
IgG (g/L)	23.22 ± 13.00	17.81 ± 5.18	0.208
IgM (g/L)	4.86 ± 3.35	3.99 ± 3.53	0.488
INR	1.03 ± 0.14	1.07 ± 0.17	0.522
PT (s)	11.56 ± 1.55	11.85 ± 1.81	0.638
Anti-gp210 (neg/pos)	5 (71.4%)/2 (28.6%)	24 (66.7%)/12 (33.4%)	0.345
Anti-sp100 (neg/pos)	6 (85.7%)/1 (14.2%)	31 (86.1%)/5 (13.9%)	0.414

PBC, primary biliary cholangitis; γ-GT, γ-glutamyl transpeptidase; ALB, albumin; ALT, alanine aminotransferase; ALP, alkaline phosphatase; AST, aspartate aminotransferase; GLO, globulin; TBIL, total bilirubin; TBA, total bile acid; Cr, creatinine; WBC, white blood cell; HB, hemoglobin; PLT, platelet; IgA, immunoglobulin A; IgG, immunoglobulin G; IgM, immunoglobulin M; INR, international standardized ratio; PT, prothrombin time; neg, negative; pos, positive; *, P < 0.05.

#### (2) Richness and diversity analyses

**Figure 3:**
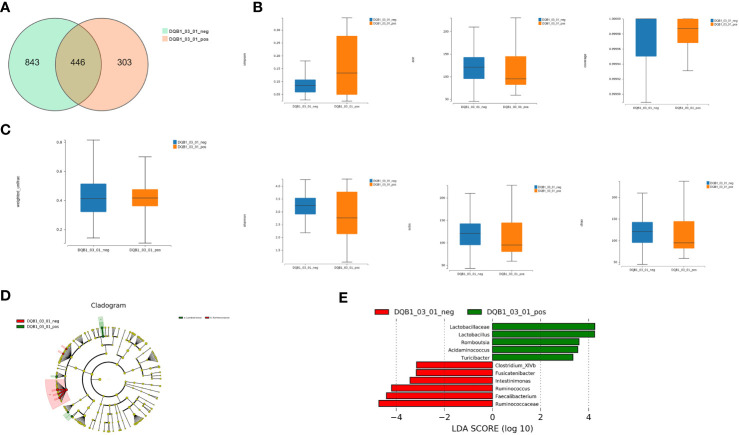
Richness and diversity analysis. **(A)** We detected 446 shared universal OTUs in samples from both DQB1*03:01-positive and DQB1*03:01-negative patients. DQB1*03:01-negative patients contained more unique OTUs than did DQB1*03:01-positive patients (843 vs. 303 OTUs). **(B)** α-diversity analysis revealed non-significant differences in the Simpson’s, Shannon’s, Sobs, ACE, Chao, and other observed species indexes between the two patient groups (*P*>0.05). **(C)** Microbial communities did not statistically differ between these two groups (*P*>0.05). **(D)** LEfSe analysis showed that the DQB1*03:01-negative patient samples contained mainly Ruminococcaceae at the family level, and the DQB1*03:01-positive samples contained mainly Lactobacillaceae. **(E)** Lactobacillaceae, *Lactobacillus*, *Romboutsia*, *Acidaminococcus*, *Turicibacter*, *Clostridium_XlVb*, *Fusicatenibacter*, *Intestinimonas*, *Ruminococcus*, *Faecalibacterium*, and Ruminococcaceae were differentially expressed among the taxonomic levels.

We detected 446 shared universal OTUs in both DQB1*03:01-positive and DQB1*03:01-negative patients. DQB1*03:01-negative patients contained more exclusive OTUs than did DQB1*03:01-positive patients (843 vs. 303; **Figure 3A**).

The α-diversity analysis revealed some differences that were not significant by the Simpson’s, Shannon’s, Sobs, ACE, Chao, or other observed species indexes between the two groups (*P*>0.05; **Figure 3B**). Likewise, the microbial communities did not statistically differ between these two groups (*P*>0.05; **Figure 3C**).

#### (3) Comparison of bacterial compositions

LEfSe analysis showed that samples from the DQB1*03:01-negative patients mainly contained Ruminococcaceae at the family level, and the DQB1*03:01-positive samples mainly contained Lactobacillaceae (**Figure 3D**). Lactobacillaceae, *Lactobacillus*, *Romboutsia*, *Acidaminococcus*, *Turicibacter*, *Clostridium_XlVb*, *Fusicatenibacter*, *Intestinimonas*, *Ruminococcus*, *Faecalibacterium*, and Ruminococcaceae were differentially expressed among the taxonomic levels (**Figure 3E**).

Although bacterial richness did not significantly differ between the DQB1*03:01-positive and DQB1*03:01-negative patients, *Lactobacillus*, *Romboutsia*, *Turicibacter*, and *Acidaminococcus* were increased, and *Clostridium_XlVb*, *Ruminococcus*, *Faecalibacterium*, and *Fusicatenibacter* were significantly decreased in patients with the protective DQB1*03:01 allele.

### Gut microbiota compositions in patients carrying high-risk alleles with or without LC

The 43 patients were divided into four subgroups: FHRAC-positive with LC (n=5), FHRAC-negative with LC (n=5), FHRAC-positive without LC (n=20), and FHRAC-negative without LC (n=13).

#### (1) Bacterial richness and diversity analyses

**Figure 4:**
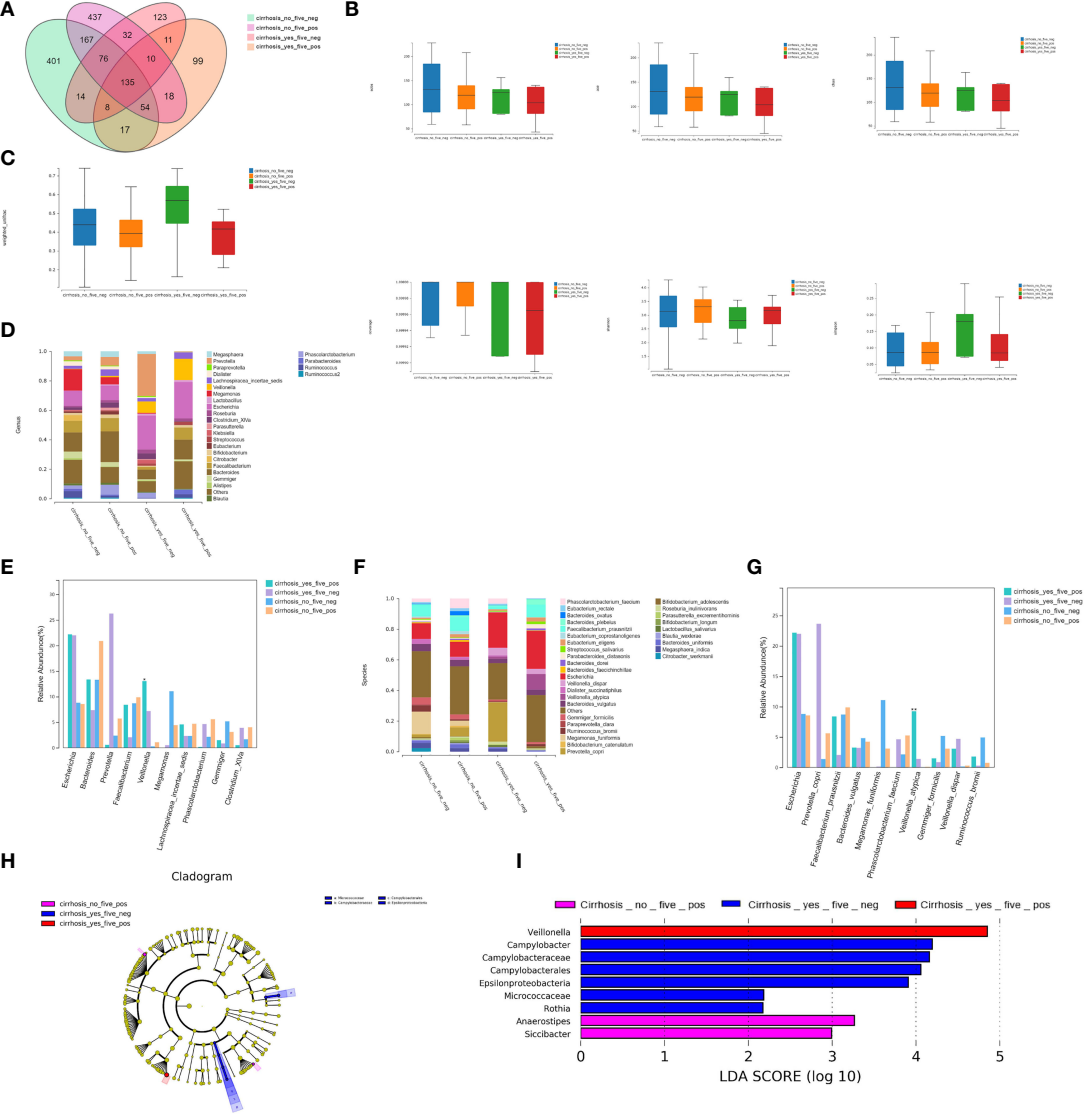
Bacterial richness and diversity analysis. **(A)** We found 135 shared universal OTUs among the four patient subgroups. **(B)** α-diversity analysis showed no significant differences in the Simpson’s, Shannon’s, Sobs, ACE, Chao, or other observed species indexes between the four groups (*P*>0.05). **(C)** The microbial communities differed statistically among the four groups (*P<*0.05). **(D)** Twenty-seven bacterial genera were detected. **(E)** The relative abundances of *Veillonella* were 13.05% for FHRAC-positive patients with LC, 7.18% for FHRAC-negative patients with LC, 0.28% for FHRAC-positive patients without LC, and 1.08% for FHRAC-negative patients without LC (all *P<*0.05). **(F)** Thirty-two bacterial species were detected. **(G)** The relative abundances of *Veillonella atypica* were 9.24%for FHRAC-positive patients with LC, 1.40% for FHRAC-negative patients with LC, 0.05% for FHRAC-positive patients without LC, and 0.10% for FHRAC-negative patients without LC (all *P<*0.05). **(H)** LEfSe analysis showed that samples from the FHRAC-negative patients with LC contained mainly Campylobacteraceae at the class level, Campylobacterales at the order level, and Micrococcaceae and Epsilonproteobacteria at the family level. **(I)** *Anaerostipes*, *Campylobacter*, *Campylobacteraceae*, *Epsilonproteobacteria*, *Veillonella*, *Rothia*, *Siccibacter*, *Micrococcaceae*, and *Campylobacterales* were differentially expressed among the taxonomic levels.

We detected 135 shared universal OTUs in the four subgroups (**Figure 4A**). The α-diversity analysis showed no significant differences in the Simpson’s, Shannon’s, Sobs, ACE, Chao, and other observed species indexes among the four groups (*P*>0.05; **Figure 4B**). However, the microbial communities differed significantly among the four groups (*P<*0.05; **Figure 4C** and **Supplementary Table S3A**).

#### (2) Comparison of bacterial compositions

(2.1) Twenty-seven bacterial genera were detected in total (**Figure 4D**). The relative abundances of *Veillonella* differed significantly among the groups at 13.05% for FHRAC-positive patients with LC, 7.18% for FHRAC-negative patients with LC, 0.28% for FHRAC-positive patients without LC, and 1.08% for FHRAC-negative patients without LC (*P<*0.05; **Figure 4E** and **Supplementary Table S3B**).

(2.2) Thirty-two bacterial species were detected in total (**Figure 4F**). The relative abundance of *Veillonella_atypica* differed significantly among the groups at 9.24% for FHRAC-positive patients with LC, 1.40% for FHRAC-negative patients with LC, 0.05% for FHRAC-positive patients without LC, and 0.10% for FHRAC-negative patients without LC (*P<*0.05; **Figure 4G** and **Supplementary Table S3C**).

LEfSe analysis showed that samples from FHRAC-negative patients with LC mainly contained Campylobacteraceae at the class level, Campylobacterales at the order level, and Micrococcaceae and Epsilonproteobacteria at the family level (**Figure 4H**). *Anaerostipes*, *Campylobacter*, Campylobacteraceae, *Epsilonproteobacteria*, *Veillonella*, *Rothia*, *Siccibacter*, Micrococcaceae, and Campylobacterales were differentially expressed among the taxonomic levels (**Figure 4I**).

### Gut microbiota compositions in patients carrying high-risk alleles with LC vs. without high-risk alleles and LC

#### (1) Bacterial richness and diversity analyses

**Figure 5:**
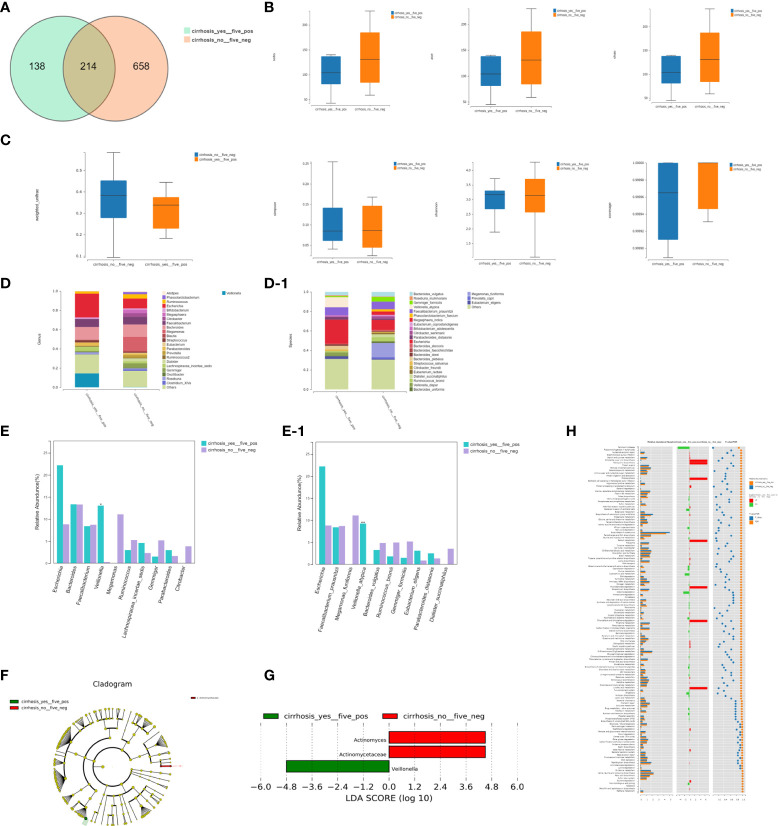
Bacterial richness, diversity, and function. **(A)** We detected 214 shared universal OTUs in FHRAC-positive patients with LC (n=5) and FHRAC-negative patients without LC (n = 13). Samples from FHRAC-positive patients with LC contained fewer unique OTUs than did FHRAC-negative patients without LC (138 vs. 658). **(B)** α-diversity analysis showed no significant differences in the Simpson’s, Shannon’s, Sobs, ACE, Chao, or other coverage indexes between the two groups (*P* > 0.05). **(C)** The microbial communities did not statistically differ between the two groups (*P*>0.05). **(D, D-1)** Twenty-four bacterial genera and 27 bacterial species were detected **(E, E-1)**. The abundances of *Veillonella* at the genus level and *Veillonella atypica* at the species level were greater in the FHRAC-positive patients with LC than in the FHRAC-negative patients without LC. **(F)** LEfSe analysis showed that the samples from the FHRAC-negative patients without LC mainly contained Antinomycetaceae at the family level. **(G)** Three flora were differentially expressed among the taxonomic levels. **(H)** We detected 151 metabolites or pathways at the KEGG level 3. The Wilcoxon test was used to compare the significantly different features. The differentially expressed microbiota were closely related to four differential metabolic pathways: plant hormone signal transduction, Parkinson’s disease, spliceosomes, and protein processing in the endoplasmic reticulum.

We detected 214 shared universal OTUs among the FHRAC-positive patients with LC (n=5) and FHRAC-negative patients without LC (n=13). The samples from FHRAC-positive patients with LC contained fewer unique OTUs than did those from FHRAC-negative patients without LC (138 vs. 658; **Figure 5A**).

The α-diversity analysis showed no significant differences on the Simpson’s, Shannon’s, Sobs, ACE, Chao, or other coverage indexes between the two groups (*P*>0.05; **Figure 5B**). The microbial communities did not differ significantly between the two groups (*P*>0.05; **Figure 5C**). However, FHRAC-positive patients with LC exhibited unique diversity and bacterial compositions compared with those of FHRAC-negative patients without LC.

#### (2) Comparison of bacterial compositions

Twenty-four bacterial genera and 27 bacterial species were detected in total (**Figures 5D, 5D-1** **)**, with some significantly different flora at both levels. The abundances of *Veillonella* at the genus level and *Veillonella atypica* at the species level were higher in FHRAC-positive patients with LC than in FHRAC-negative patients without LC (**Figures 5E, 5E-1** **)**.

LEfSe analysis showed that samples from the FHRAC*-*negative patients without LC mainly contained Antinomycetaceae at the family level (**Figure 5F**). Three flora were differentially expressed among the taxonomic levels (**Figure 5G**).

#### (3) Association between featured bacterial taxa and pathways

We used PICRUSt to predict the functional pathways based on the 16S rRNA gene to obtain information on the metabolites or pathways potentially involved with PBC, and 151 metabolites or pathways were detected at the KEGG level 3. We used the Wilcoxon test to compare the significantly different features. The differentially expressed microbiota were closely related to four differential metabolic pathways: plant hormone signal transduction, Parkinson’s disease, spliceosomes, and protein processing in the endoplasmic reticulum (**Figure 5H**).

## Discussion

We analyzed the associations between the HLA class II genes (including susceptible, high-risk, and protective alleles) and the gut microbiome compositions and distributions in patients with PBC. Some characteristics of the HLA class II genes and gut microbiome distributions in patients with PBC and LC were observed. First, we confirmed HLA-*DRB1* high-risk alleles in these patients. The results suggested that the gut species richness and microbiome compositions were lower in patients with high-risk alleles than in those without high-risk alleles. Next, using LEfSe analysis of taxonomic classifications, significant associations were noted between patients with high-risk alleles and those with protective alleles. Furthermore, in patients with PBC and LC with high-risk alleles, some flora were increased significantly compared with those of patients without high-risk alleles and LC. Finally, we observed different flora distributions with biochemical markers and pathways.

The pathogenesis and progression mechanisms of PBC are complex and poorly understood. However, environmental influences likely play significant roles in driving PBC development and progression, interacting with immunogenetic and epigenetic risks (12–14). Previous reports described the correlation between susceptible HLA genes and PBC and between intestinal flora and PBC, and similar studies have been conducted in other diseases with LC (15–17). We focused on the microbiota composition and functional features in patients with PBC with susceptible or protective HLA alleles. To avoid having too few samples of a single allele affecting the statistical results, we set up an FHRAC group, which combined five high-risk HLA-DRB1 alleles (DRB1*03:01, DRB1*07:01, DRB1*08:03, DRB1*14:05, and DRB1*14:54), then analyzed the associations between these high-risk HLA alleles and patients’ gut microbiota. In the FHRAC group, species richness and microbiome compositions were decreased in patients with high-risk HLA alleles compared with those in patients without high-risk HLA alleles. Thus, the HLA class II genes may influence the gut microbiome compositions of patients with PBC.

Microbiota compositions are reported to be significantly correlated with poor prognoses for patients with PBC (10). Some intestinal microflora have been associated with bile acid metabolism in PBC, and some microbes produced by short-chain fatty acids were significantly decreased in patients with PBC with inferior remission (6). Chen et al. reported decreased taurine-conjugated TBA in patients with PBC treated with ursodeoxycholic acid (UDCA) (18), raising questions regarding the distribution characteristics of the microbiome compositions of patients with PBC influenced by the HLA II gene. We further analyzed the different microbiota between the HLA groups and found that the gut species richness and microbiome compositions were lower in patients with high-risk alleles than in those without high-risk alleles.

LEfSe analysis of the taxonomic classification levels showed significant associations between patients with high-risk HLA genes. *Lachnospiraceae_incertae_sedis* and *Anaerostipes* were significantly decreased, whereas *Campylobacter*, *Lachnospira*, *Desulfovibrio*, *Klebsiella* and *Barnesiella* were significantly increased in FHRAC-positive patients. Additionally, *Lactobacillus*, *Romboutsia*, *Turicibacter*, and *Acidaminococcus* were increased, whereas *Clostridium_XlVb*, *Ruminococcus*, *Faecalibacterium*, and *Fusicatenibacter* were decreased in patients carrying DQB1*03:01.

Does genetic susceptibility affect disease progression? Genetic predisposition to autoimmune hepatitis (AIH) has been associated with HLA alleles. Ma et al. (19) studied 236 children of European ancestry. Possession of homozygous DRB1*03 or of DRB1*13 was associated with fibrosis at disease onset, and possession of both genes along with DRB1*07 was associated with a more severe disease in three subgroups of patients with AIH, including type 1 and type 2 AIH and autoimmune cholangitis. Therefore, we analyzed the interactions between LC, HLA, and the intestinal flora in patients with PBC. First, patients were divided into four groups, then two subgroups according to the presence of LC and HLA alleles. Interestingly, their microbiome compositions differed significantly. *Veillonella* and *Veillonella atypica* abundances differed among the four groups, with the highest in patients with LC and FHRAC, and the lowest in patients without LC and FHRAC. Antinomycetaceae abundances also differed. Because of limitations in our four-group analysis, we further analyzed these two subgroups. *Veillonella* were significantly higher in patients with LC and FHRAC than in those without LC or FHRAC. A literature reviewed revealed that the levels of secondary bile acids such as deoxycholic acid (DCA) and conjugated DCA were inversely correlated with PBC-enriched gut microbes (*Veillonella*) (18). *Veillonella*, which is associated with chronic inflammatory and fibrotic conditions, was enriched in primary sclerosing cholangitis (20). *Veillonella* is also reported to be associated with urinary tract infections (UTIs) (21). UTI appears to be the only bacterial infection identified as a risk factor for PBC. *Escherichia coli* is a predominant pathogen in most UTI cases, and its infection is a key factor in breaking immunological tolerance through molecular mimicry mechanisms (22). Our results showed for the first time that *Veillonella* was increased in patients with PBC and LC carrying susceptible HLA genes. However, this finding requires further investigation in larger samples of patients with PBC to identify its pathway.

A significant proportion of the microbiota can be linked to maintaining intestinal and cellular homeostasis. The microbiota helps modulate histone deacetylases, thus affecting immune cell immigration, chemotaxis, programmed cell death, cytokine production, and cell attachment. Therefore, the relative abundances of differentially expressed microbes in diseases should be considered in disease treatment and prevention (23, 24).

Previous studies showed that some of the above intestinal flora are related to liver metabolism and function. *Anaerostipes* is closely related to bile acid metabolism, and its abundance is inversely correlated with bile acid levels in UDCA-treated patients with PBC (25). Ruminococcaceae is closely related to bile acid metabolism, functions in 7α-dehydroxylation, and is important for bile-acid synthesis (26). Leclercq et al. reported that Lachnospiraceae and *Clostridiales cluster XIV* were present and specifically increased in the stools of patients with mild hepatic fibrosis (27). Accumulation of partially undegraded glycosaminoglycans causes severe and chronic disturbances in liver function (28).

We also correlated different flora with biochemical indicators and pathways. Differentially expressed microbiota in patients with high-risk HLA DRB1 alleles may influence the pathway related to glycosaminoglycan metabolism. *Lachnospiraceae_incertae_sedis* was positively correlated with serum IgG levels. Disrupted microbial balances in both short- and long-term conditions can severely reduce microbial community richness and diversity (17). Genetic variations of major histocompatibility complex (MHC) genes among individuals mediate disease susceptibility by controlling microbiota diversity. An individual MHC genotype against the microbiota may result in compositional differences (29). HLA likely influences specific bacterial abundances because differences in community member overlap vary significantly by genetic risk (9). Our results indicated that the HLA genes were related to PBC pathogenesis and disease progression, and pathways corresponding to different intestinal flora may mediate this process.

Our results indicate that the HLA class II genes may influence the gut microbiome composition in patients with PBC. However, bacterial associations with HLA and clinical features require confirmation in a larger sample of patients. Personalized medicine is an emerging practice in modern medicine, which is committed to surveying, monitoring, and diagnosing risks to provide patients with a specific treatment, taking into account their particular genetic profile, molecular phenotype, and intestinal flora. Our study will be helpful for the personalized-medicine approach for patients with PBC.

## Data availability statement

The data presented in the study are deposited in the NCBI repository, accession number PRJNA875394.

## Ethics statement

The studies involving human participants were reviewed and approved by The Institutional Ethics Review Board of Beijing YouAn Hospital, Capital Medical University (Ethics: No. [2012] 44 and No. LL-2018-044-K). The patients/participants provided their written informed consent to participate in this study.

## Author contributions

Conception and design: H-PY, Y-ML, C-YH, and H-PZ. Patient’s follow up and clinical data collection: Y-ML, C-YH, W-JH, H-YL, BX, YH, X-DZ, JZ, and W-JL. Sample collection, detection, and analysis: H-PZ, D-TZ, Y-XM, L-JL, X-HL, QW, and J-LL. Data analysis and manuscript writing: C-YH, W-JH, H-PZ, Y-ML, and H-PY. Final approval of the manuscript: All authors.

## Funding

This work was supported by Beijing Municipal Administration of Hospitals Incubating Program (Code: PX2019062); WBE Liver Foundation (Grant No. WBE2022018); 2020 Science and technology innovation competition of Beijing YouAn Hospital, Capital Medical University (YAKJCXDS202003); and Young and Middle-aged Talent Incubation Project of Beijing YouAn Hospital, Capital Medical University, 2021 (Youth Innovation) (YNKTQN2021004).

## Acknowledgments

The authors highly appreciate all patients who participated in the study.

## Conflict of interest

The authors declare that the research was conducted in the absence of any commercial or financial relationships that could be construed as a potential conflict of interest.

The reviewer JJ declared a shared parent affiliation with the authors C-YH, H-PZ, D-TZ, H-YL, Y-XM, BX, L-JL, YH, X-HL, QW, J-LL, X-DZ, JZ, W-JL, Y-ML, and H-PY to the handling editor at the time of review.

## Publisher’s note

All claims expressed in this article are solely those of the authors and do not necessarily represent those of their affiliated organizations, or those of the publisher, the editors and the reviewers. Any product that may be evaluated in this article, or claim that may be made by its manufacturer, is not guaranteed or endorsed by the publisher.
