# Supplementary material for: Disease predisposition of human leukocyte antigen class II genes influences the gut microbiota composition in patients with primary biliary cholangitis
Source: Front Immunol. 2022 Sep 20;13:984697. doi: 10.3389/fimmu.2022.984697 (PMC9531677; doi:10.3389/fimmu.2022.984697)
Supplement: Supplementary file 1 [file DataSheet_1.zip › supplementary table S2A.docx]

**TABLE S2A** | The relative abundance of PBC in FHRAC positive and FHRAC negative groups

| **microbiota** | **FHRAC negative** | **FHRAC positive** |
| --- | --- | --- |
| *Bacteroides* | 0.110313096 | 0.193156343 |
| *Escherichia* | 0.140121027 | 0.12843802 |
| *Others* | 0.134794858 | 0.12304278 |
| *Faecalibacterium* | 0.061334293 | 0.089271627 |
| *Phascolarctobacterium* | 0.026150183 | 0.053238218 |
| *Prevotella* | 0.103196257 | 0.047798982 |
| *Lachnospiracea_incertae_sedis* | 0.021396362 | 0.043697044 |
| *Megamonas* | 0.104395131 | 0.038549414 |
| *Veillonella* | 0.024434529 | 0.037149701 |
| *Megasphaera* | 0.029487026 | 0.030733325 |
| *Clostridium_XlVa* | 0.023016162 | 0.029313148 |
| *Gemmiger* | 0.034370042 | 0.025809772 |
| *Bifidobacterium* | 0.012504133 | 0.0224169 |
| *Eubacterium* | 0.006070777 | 0.016794513 |
| *Roseburia* | 0.015301969 | 0.016557135 |
| *Dialister* | 0.023755213 | 0.016394449 |
| *Ruminococcus* | 0.032154278 | 0.015265879 |
| *Parasutterella* | 0.00268809 | 0.013458939 |
| *Blautia* | 0.010959349 | 0.01218917 |
| *Parabacteroides* | 0.011769249 | 0.011369601 |
| *Streptococcus* | 0.00887278 | 0.009164643 |
| *Lactobacillus* | 0.004314836 | 0.00876151 |
| *Ruminococcus2* | 0.005312277 | 0.007932732 |
| *Paraprevotella* | 0.00632361 | 0.004364895 |
| *Alistipes* | 0.009068657 | 0.003034759 |
| *Klebsiella* | 0.01051064 | 0.001951209 |
