# Supplementary material for: Disease predisposition of human leukocyte antigen class II genes influences the gut microbiota composition in patients with primary biliary cholangitis
Source: Front Immunol. 2022 Sep 20;13:984697. doi: 10.3389/fimmu.2022.984697 (PMC9531677; doi:10.3389/fimmu.2022.984697)
Supplement: Supplementary file 1 [file DataSheet_1.zip › supplementary table S2B .docx]

**TABLE S2B** | The relative abundance of PBC in FHRAC positive and FHRAC negative groups

| **microbiota** | **FHRAC**  **negative (%)** | **FHRAC**  **positive (%)** | ***P*-Value** |
| --- | --- | --- | --- |
| *Bacteroides* | 19.38071 | 11.65469 | 0.063072 |
| *Escherichia* | 11.28667 | 12.4688 | 0.901998 |
| *Faecalibacterium* | 9.587646 | 6.85624 | 0.283579 |
| *Prevotella* | 4.694334 | 9.004695 | 0.90619 |
| *Megamonas* | 3.545005 | 8.14106 | 0.583256 |
| *Phascolarctobacterium* | 4.498902 | 2.827609 | 0.889958 |
| *Lachnospiracea_incertae_sedis* | 4.67435 | 2.311397 | 0.033214 |
| *Gemmiger* | 2.757939 | 3.977127 | 0.638899 |
| *Megasphaera* | 2.967106 | 2.780556 | 0.227812 |
| *Veillonella* | 3.4755 | 2.195352 | 0.629631 |
