# Supplementary material for: Disease predisposition of human leukocyte antigen class II genes influences the gut microbiota composition in patients with primary biliary cholangitis
Source: Front Immunol. 2022 Sep 20;13:984697. doi: 10.3389/fimmu.2022.984697 (PMC9531677; doi:10.3389/fimmu.2022.984697)
Supplement: Supplementary file 1 [file DataSheet_1.zip › supplementary table S3A.docx]

**TABLE S3A** | The statistical significance of microbial communities in four groups of patients

| **Comparison group** | ***P*-Value** |
| --- | --- |
| cirrhosis_no_five_neg-vs-cirrhosis_no_five_pos-vs-cirrhosis_yes_five_neg-vs-cirrhosis_yes_five_pos | 0.010861 |

Abbreviation: neg, negative; pos, positive.
