# Supplementary material for: Disease predisposition of human leukocyte antigen class II genes influences the gut microbiota composition in patients with primary biliary cholangitis
Source: Front Immunol. 2022 Sep 20;13:984697. doi: 10.3389/fimmu.2022.984697 (PMC9531677; doi:10.3389/fimmu.2022.984697)
Supplement: Supplementary file 1 [file DataSheet_1.zip › supplementary table S4B.docx]

**TABLE S4B** | The relative abundance of the four groups of patients

| **Top 10 microbes** | **cirrhosis_yes_five_pos.(%)** | **cirrhosis_yes_five_neg.(%)** | **cirrhosis_no_five_neg.(%)** | **cirrhosis_no_five_pos.(%)** | ***P*-Value** |
| --- | --- | --- | --- | --- | --- |
| *Escherichia* | 22.197258 | 21.9953 | 8.804754 | 8.559023 | 0.328189 |
| *Bacteroides* | 13.358832 | 7.346739 | 13.311595 | 20.886184 | 0.183573 |
| *Prevotella* | 0.575609 | 26.249851 | 2.371942 | 5.724016 | 0.545346 |
| *Faecalibacterium* | 8.403896 | 2.049142 | 8.705123 | 9.883584 | 0.349234 |
| *Veillonella* | 13.048839 | 7.180705 | 0.277909 | 1.082165 | 0.020903 |
| *Megamonas* | 0.004483 | 0.511313 | 11.075578 | 4.430135 | 0.861602 |
| *Lachnospiracea_incertae_sedis* | 4.568378 | 2.317046 | 2.309224 | 4.700842 | 0.177918 |
| *Phascolarctobacterium* | 0.169637 | 4.637376 | 2.131544 | 5.581218 | 0.542434 |
| *Gemmiger* | 1.483131 | 0.860266 | 5.175919 | 3.07664 | 0.179952 |
| *Clostridium_XlVa* | 0.512491 | 3.905134 | 1.662098 | 4.015843 | 0.095368 |

Abbreviation: neg, negative; pos, positive.
