# Supplementary material for: Disease predisposition of human leukocyte antigen class II genes influences the gut microbiota composition in patients with primary biliary cholangitis
Source: Front Immunol. 2022 Sep 20;13:984697. doi: 10.3389/fimmu.2022.984697 (PMC9531677; doi:10.3389/fimmu.2022.984697)
Supplement: Supplementary file 1 [file DataSheet_1.zip › supplementary table S4C.docx]

**TABLE S4C** | The relative abundance of the four groups of patients

| **Top 10 microbes** | **cirrhosis_yes_five_pos.(%)** | **cirrhosis_yes_five_neg.(%)** | **cirrhosis_no_five_neg.(%)** | **cirrhosis_no_five_pos.(%)** | ***P*-Value** |
| --- | --- | --- | --- | --- | --- |
| *Escherichia* | 22.197258 | 21.9953 | 8.803788 | 8.559023 | 0.328189 |
| *Prevotella_copri* | 0.01031 | 23.653344 | 1.379714 | 5.615985 | 0.572102 |
| *Faecalibacterium_prausnitzii* | 8.403896 | 2.049142 | 8.705123 | 9.883584 | 0.349234 |
| *Bacteroides_vulgatus* | 3.256061 | 3.206519 | 4.823895 | 4.230889 | 0.887605 |
| *Megamonas_funiformis* | 0.004483 | 0.137454 | 11.075578 | 3.089278 | 0.849011 |
| *Megamonas_funiformis* | 0.004483 | 0.137454 | 11.075578 | 3.089278 | 0.849011 |
| *Phascolarctobacterium_faecium* | 0.023022 | 4.637376 | 2.131544 | 5.262375 | 0.350223 |
| *Veillonella_atypica* | 9.241936 | 1.397047 | 0.04922 | 0.103122 | 0.001595 |
| *Gemmiger_formicilis* | 1.483131 | 0.860266 | 5.175919 | 3.07664 | 0.179952 |
| *Veillonella_dispar* | 3.0697 | 4.687385 | 0.148998 | 0.283631 | 0.139257 |
| *Ruminococcus_bromii* | 1.786679 | 0.281256 | 4.939285 | 0.73935 | 0.570109 |

Abbreviation: neg, negative; pos, positive.
