# Supplementary figures and images for: Disease predisposition of human leukocyte antigen class II genes influences the gut microbiota composition in patients with primary biliary cholangitis
Source: Front Immunol. 2022 Sep 20;13:984697. doi: 10.3389/fimmu.2022.984697 (PMC9531677; doi:10.3389/fimmu.2022.984697)

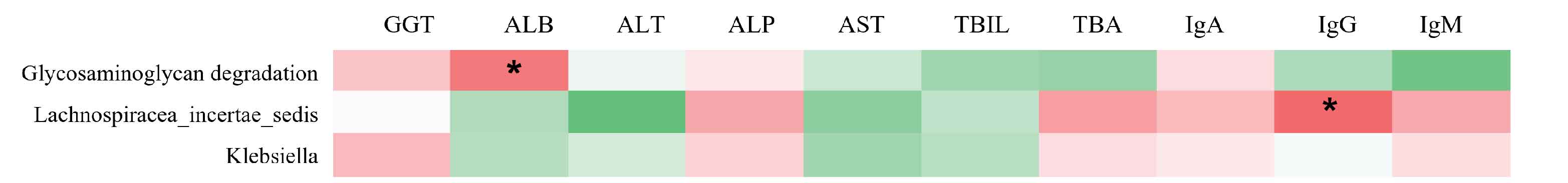

Supplement: Supplementary file 2 [file DataSheet_2.zip › Figure2-5/Figure2/figure 2I.jpg]

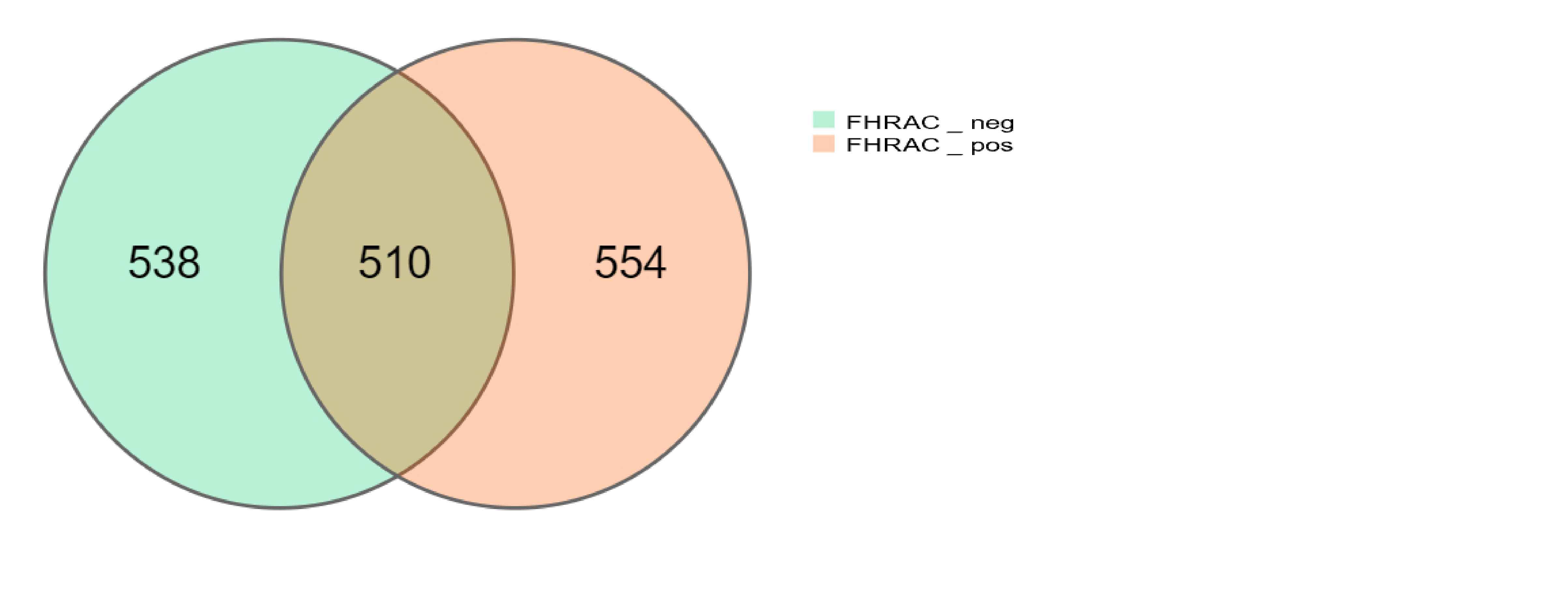

Supplement: Supplementary file 2 [file DataSheet_2.zip › Figure2-5/Figure2/Figure2A .jpg]

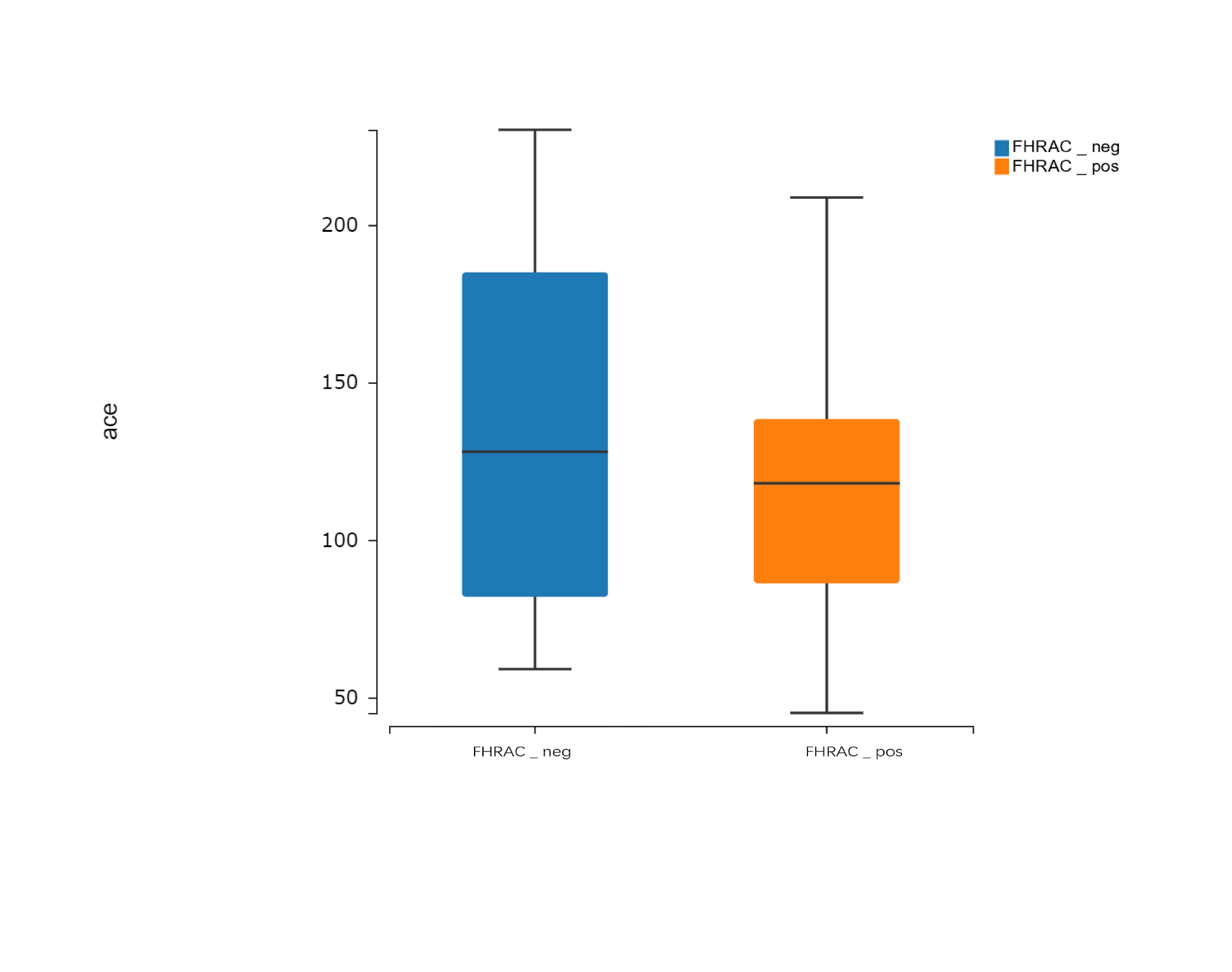

Supplement: Supplementary file 2 [file DataSheet_2.zip › Figure2-5/Figure2/Figure2B ace.jpg]

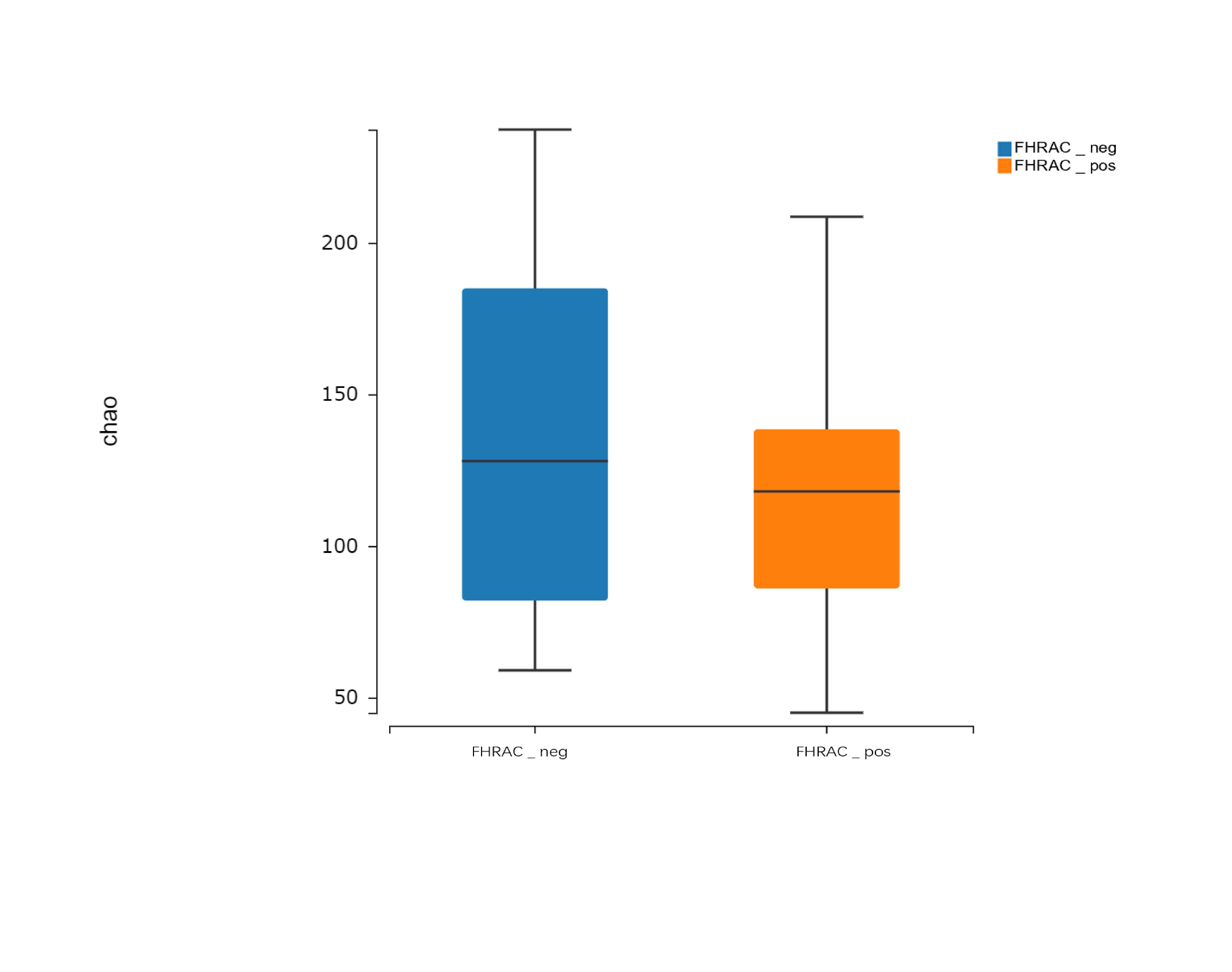

Supplement: Supplementary file 2 [file DataSheet_2.zip › Figure2-5/Figure2/Figure2B chao.jpg]

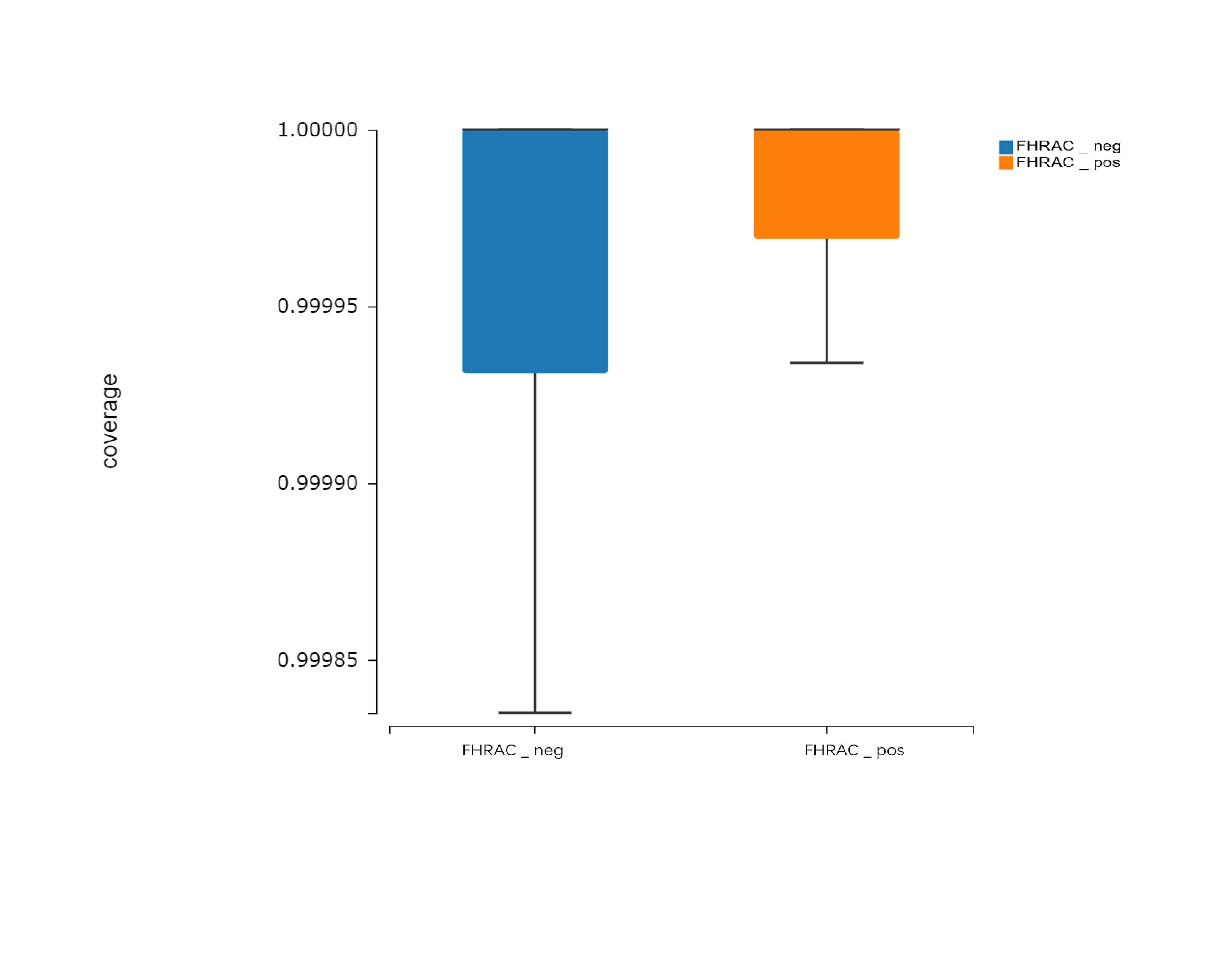

Supplement: Supplementary file 2 [file DataSheet_2.zip › Figure2-5/Figure2/Figure2B coverage.jpg]

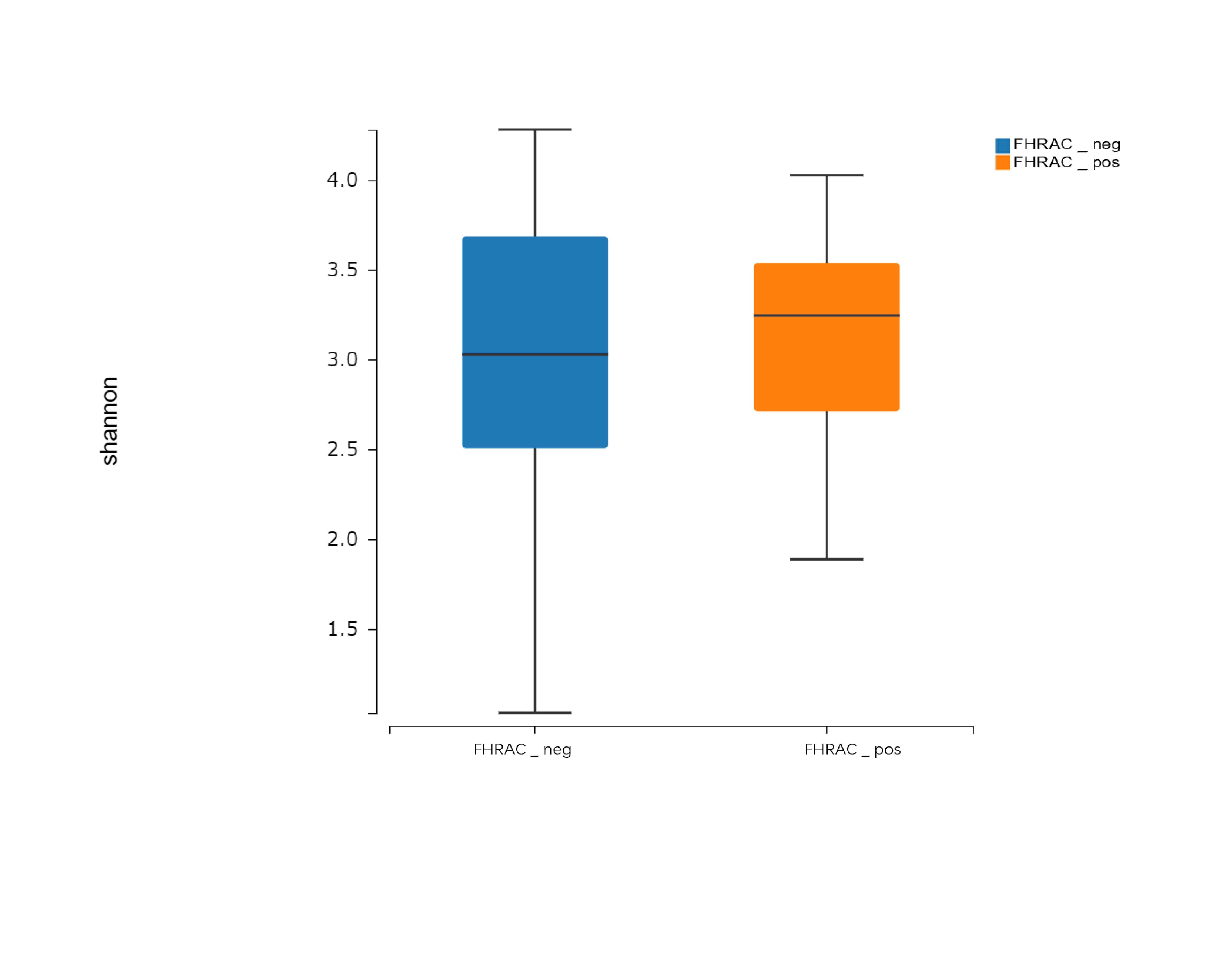

Supplement: Supplementary file 2 [file DataSheet_2.zip › Figure2-5/Figure2/Figure2B shannon.jpg]

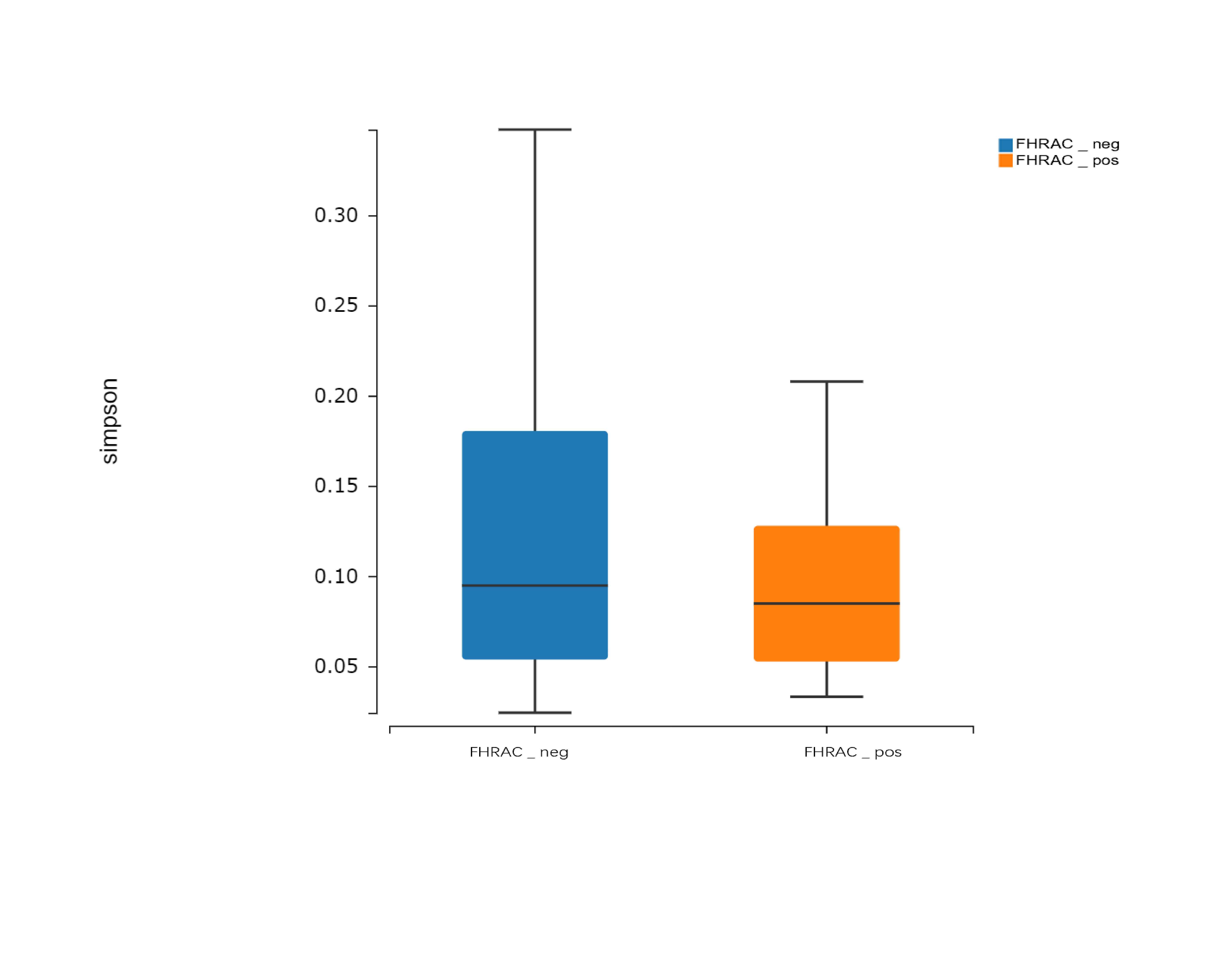

Supplement: Supplementary file 2 [file DataSheet_2.zip › Figure2-5/Figure2/Figure2B simpson.jpg]

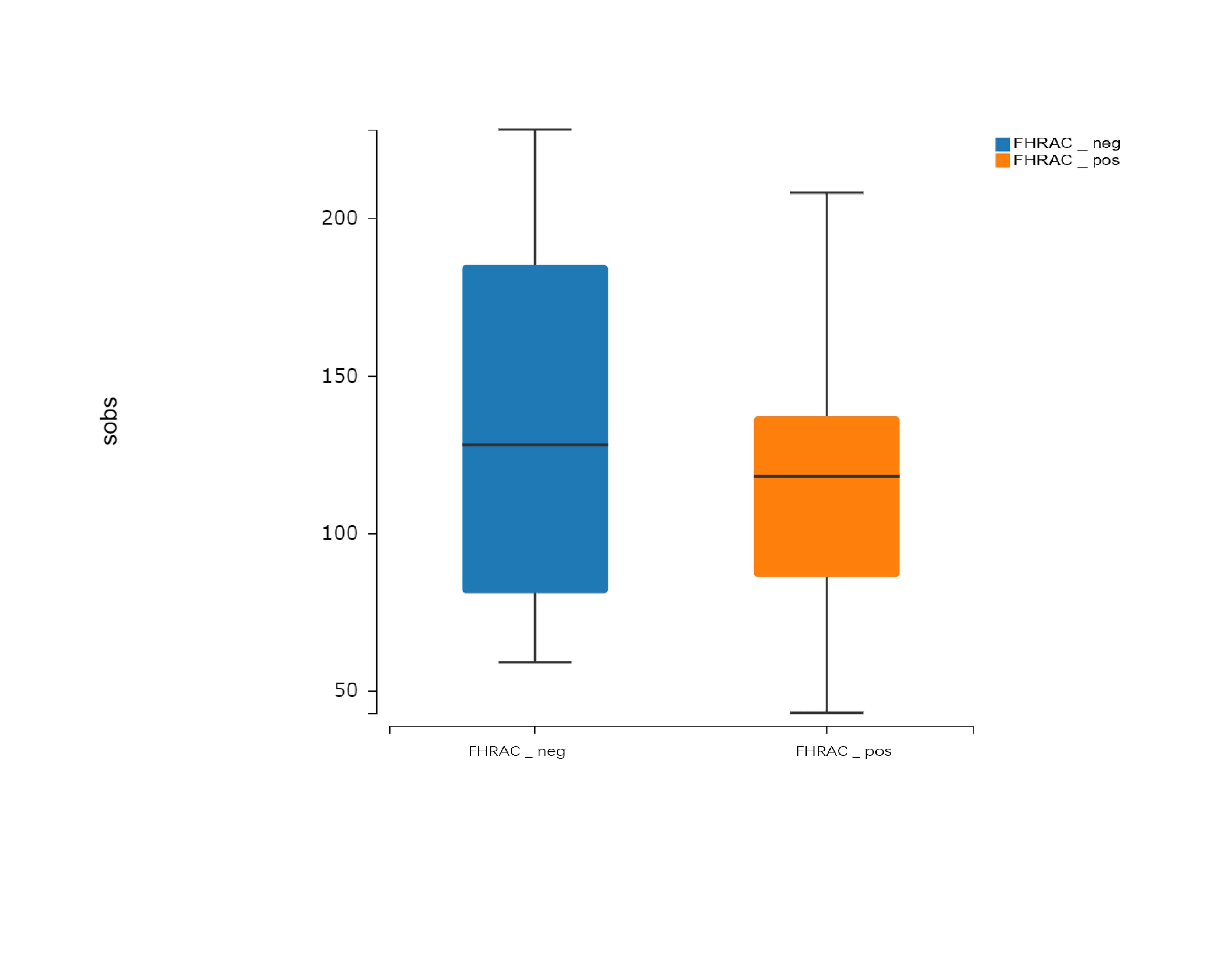

Supplement: Supplementary file 2 [file DataSheet_2.zip › Figure2-5/Figure2/Figure2B sobs.jpg]

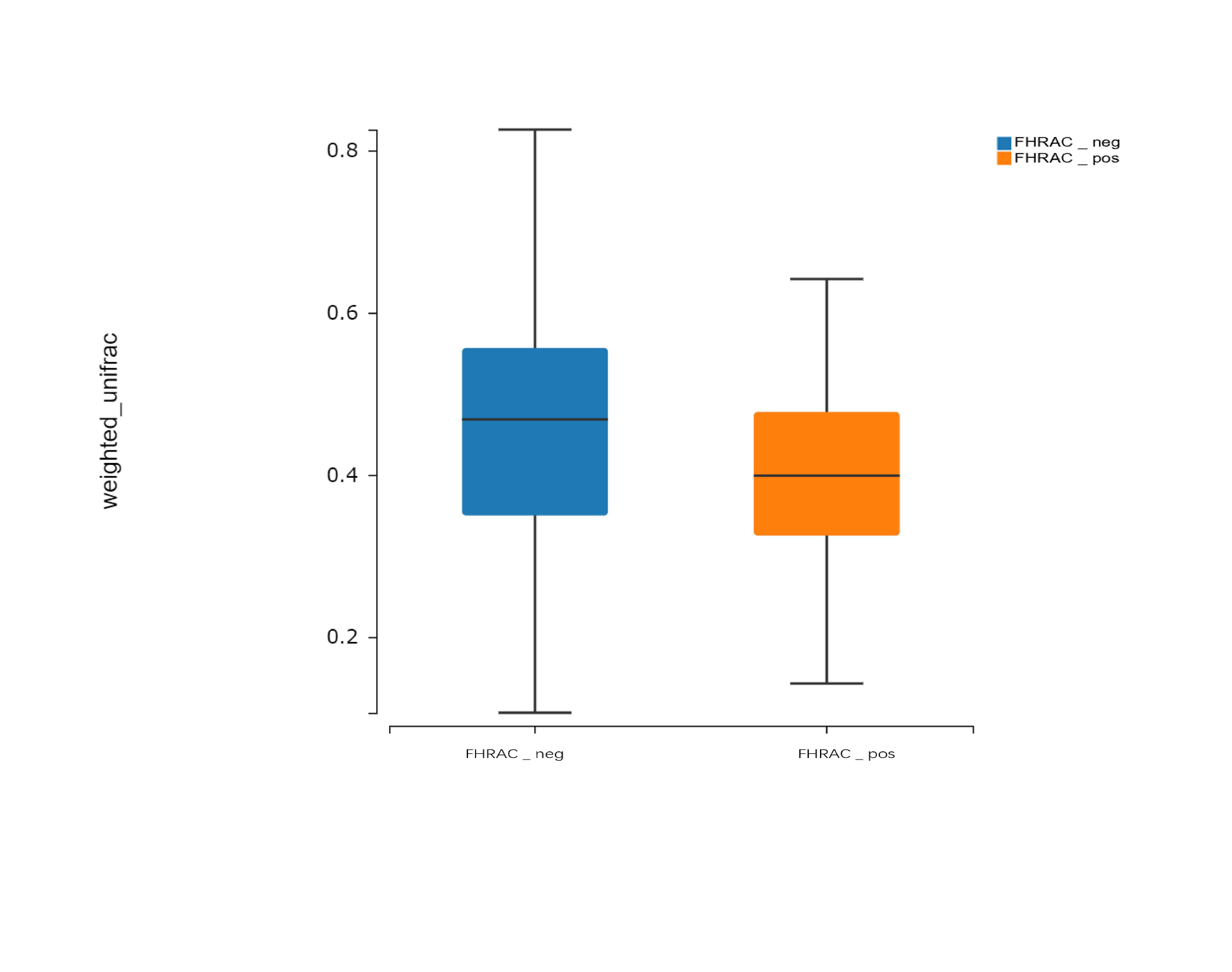

Supplement: Supplementary file 2 [file DataSheet_2.zip › Figure2-5/Figure2/Figure2C .jpg]

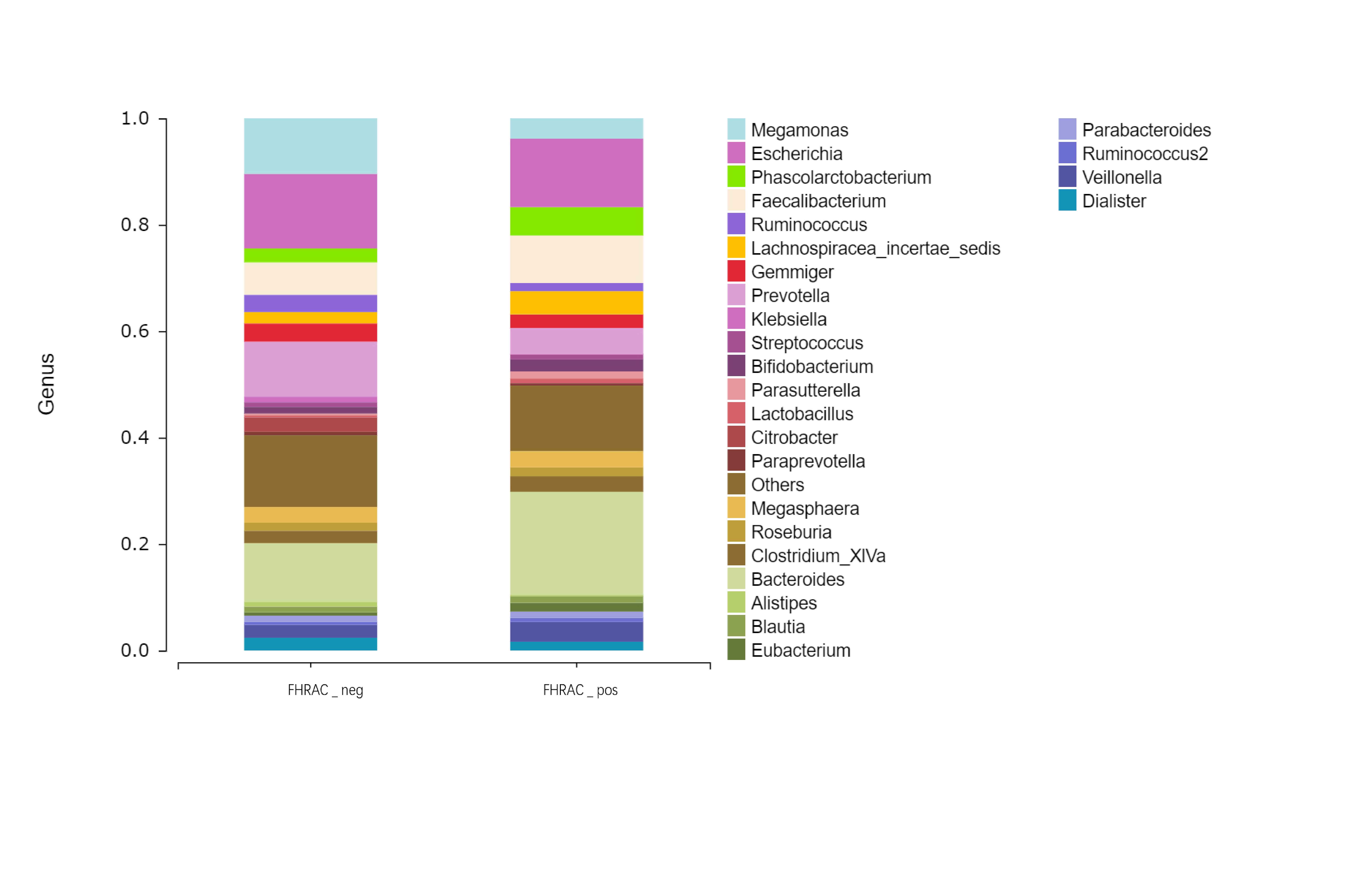

Supplement: Supplementary file 2 [file DataSheet_2.zip › Figure2-5/Figure2/Figure2D .jpg]

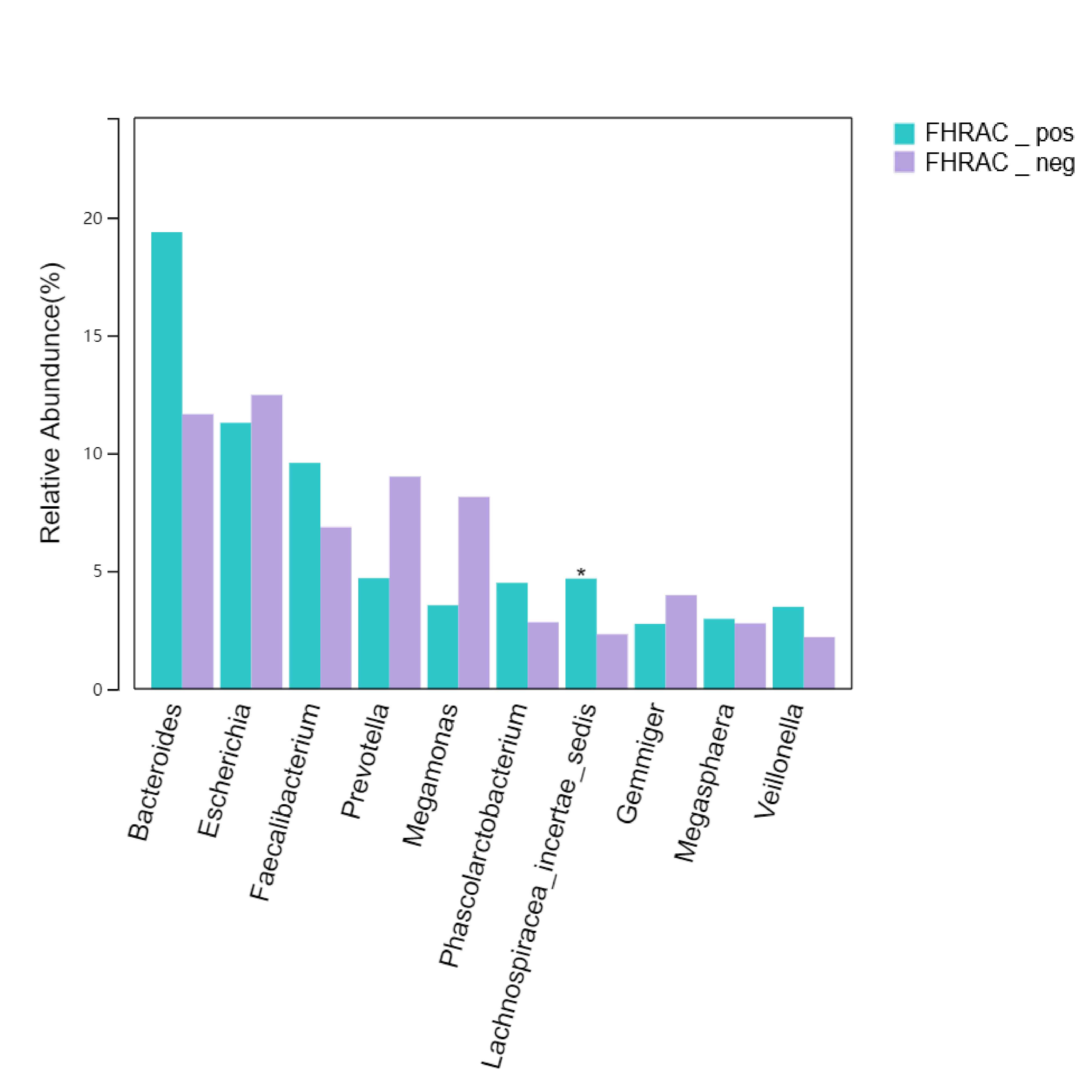

Supplement: Supplementary file 2 [file DataSheet_2.zip › Figure2-5/Figure2/Figure2E.jpg]

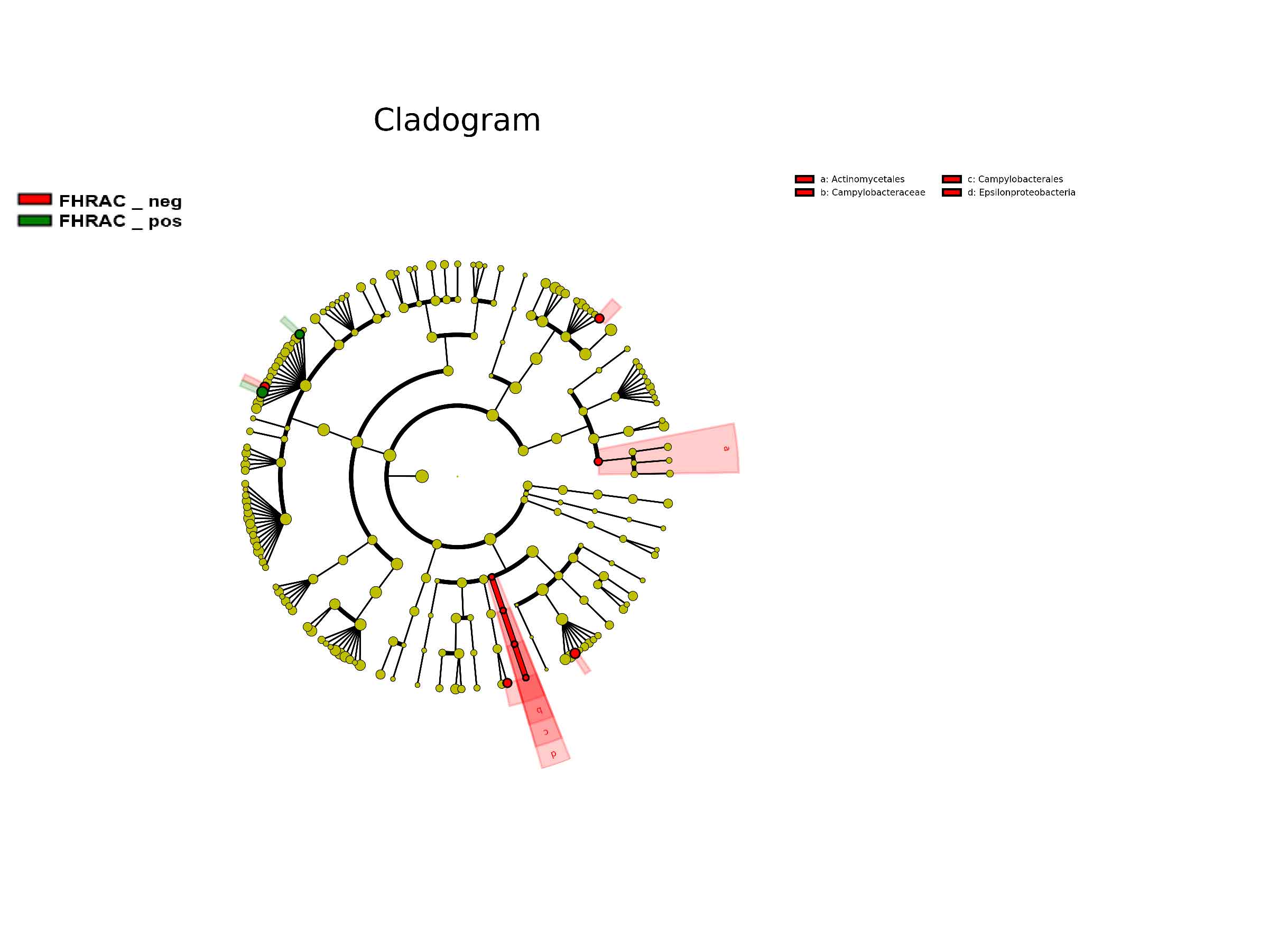

Supplement: Supplementary file 2 [file DataSheet_2.zip › Figure2-5/Figure2/Figure2F .jpg]

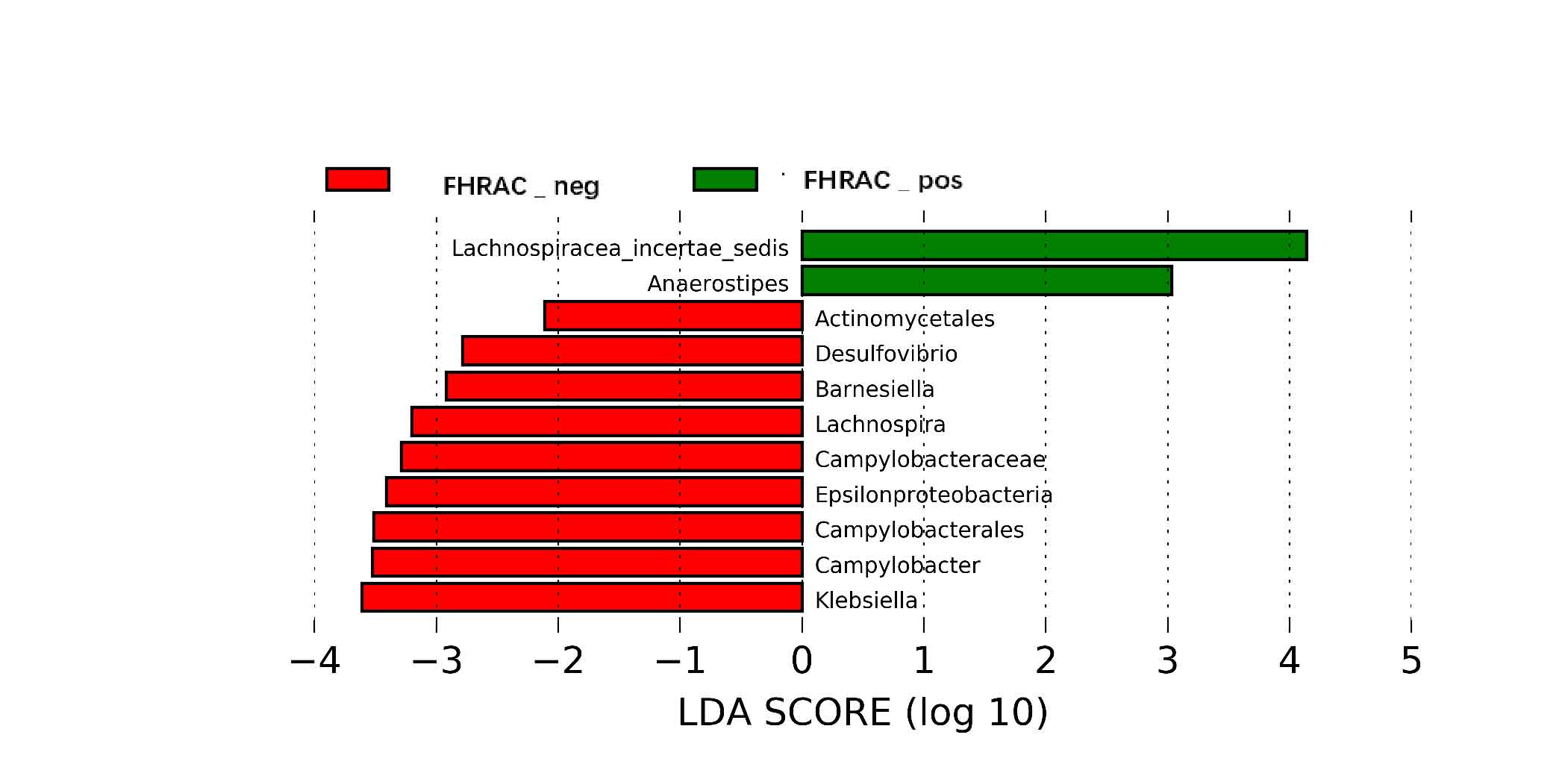

Supplement: Supplementary file 2 [file DataSheet_2.zip › Figure2-5/Figure2/Figure2G .jpg]

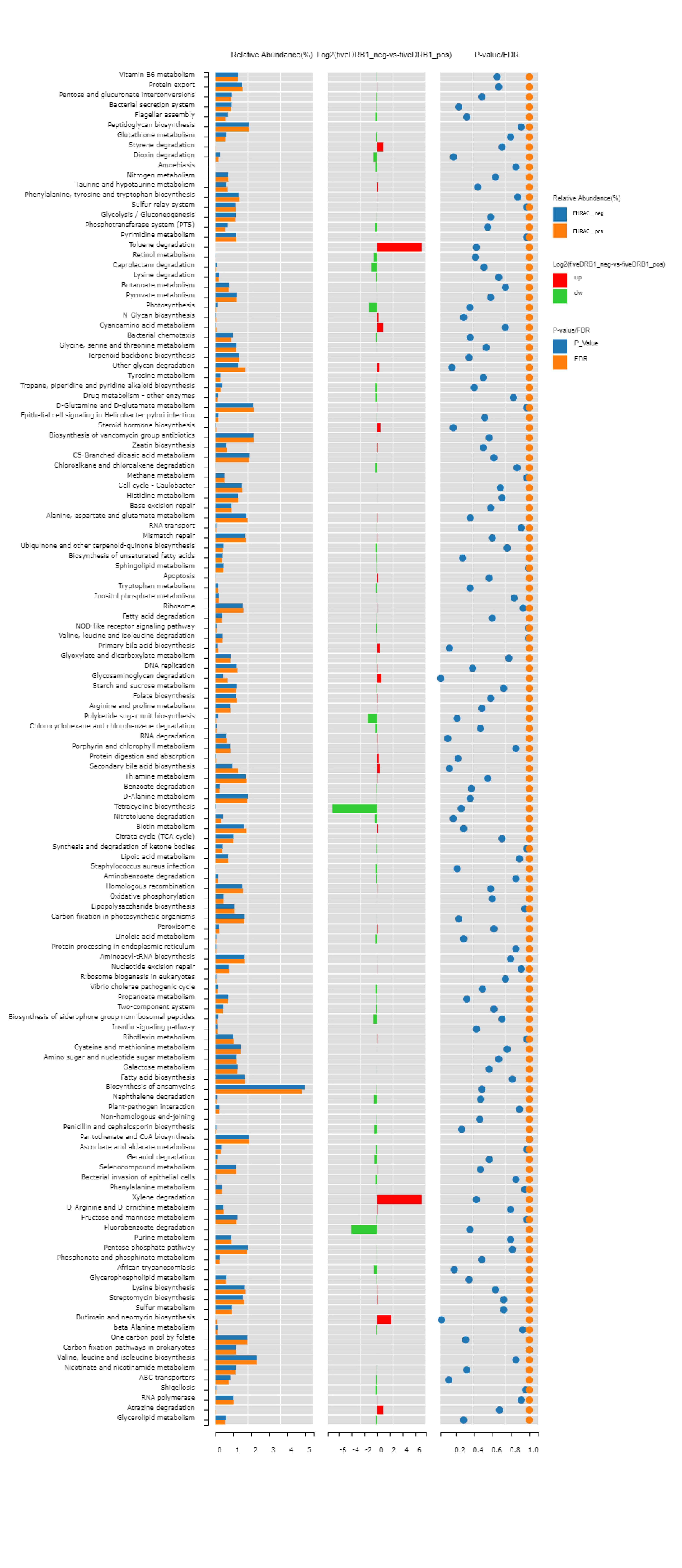

Supplement: Supplementary file 2 [file DataSheet_2.zip › Figure2-5/Figure2/Figure2H.jpg]

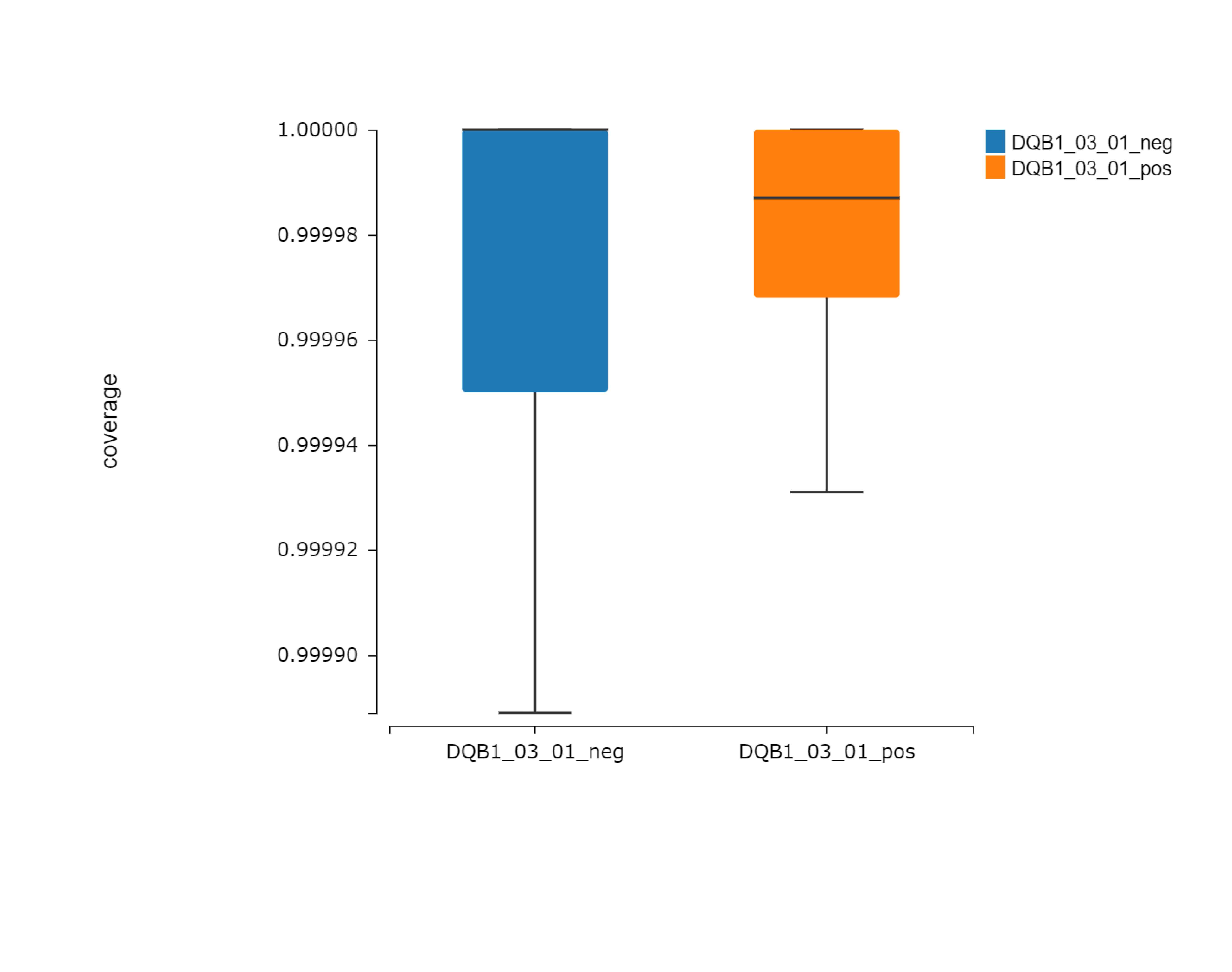

Supplement: Supplementary file 2 [file DataSheet_2.zip › Figure2-5/Figure3/Figure3 B coverage.jpg]

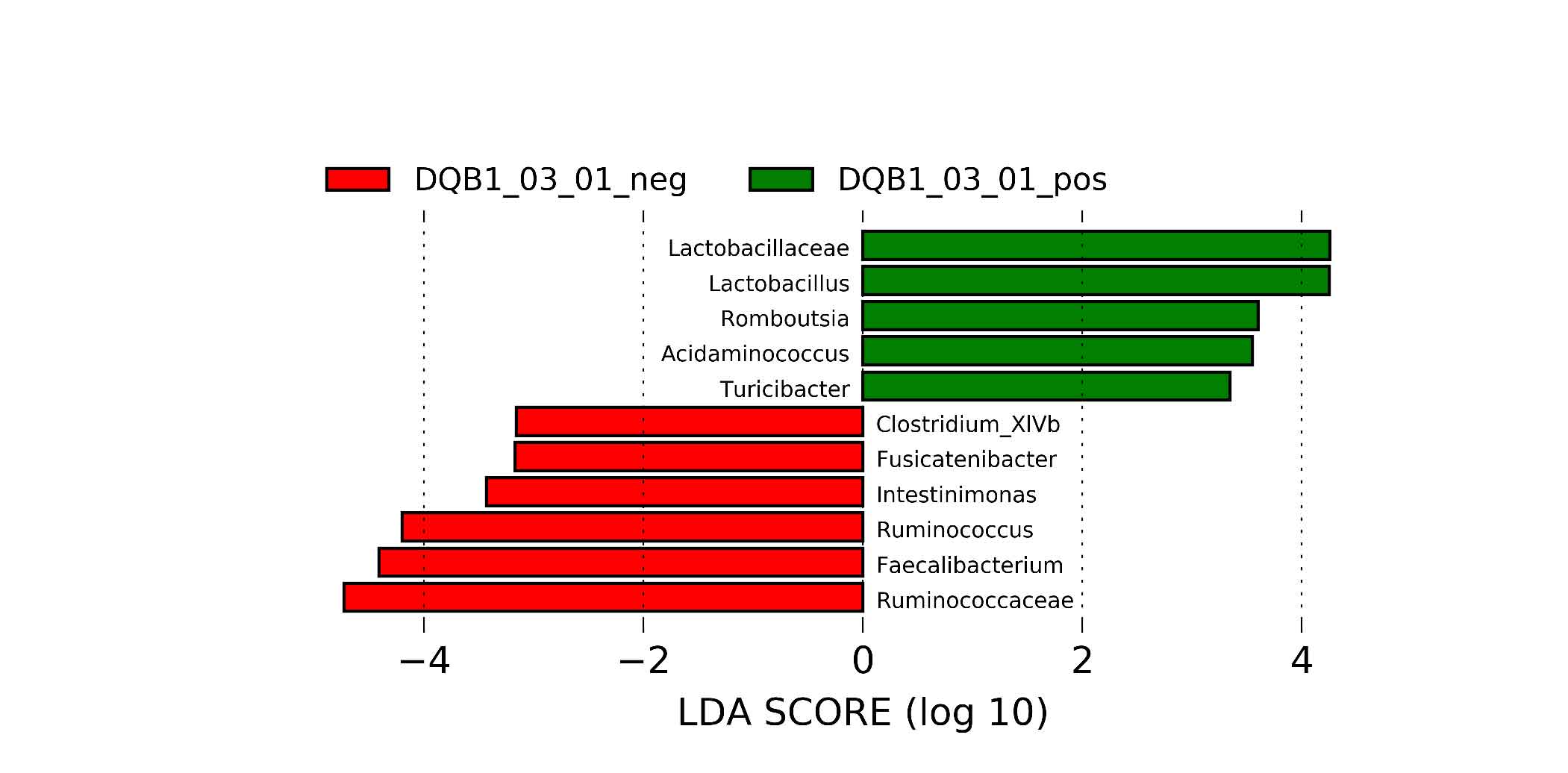

Supplement: Supplementary file 2 [file DataSheet_2.zip › Figure2-5/Figure3/Figure3 E.jpg]

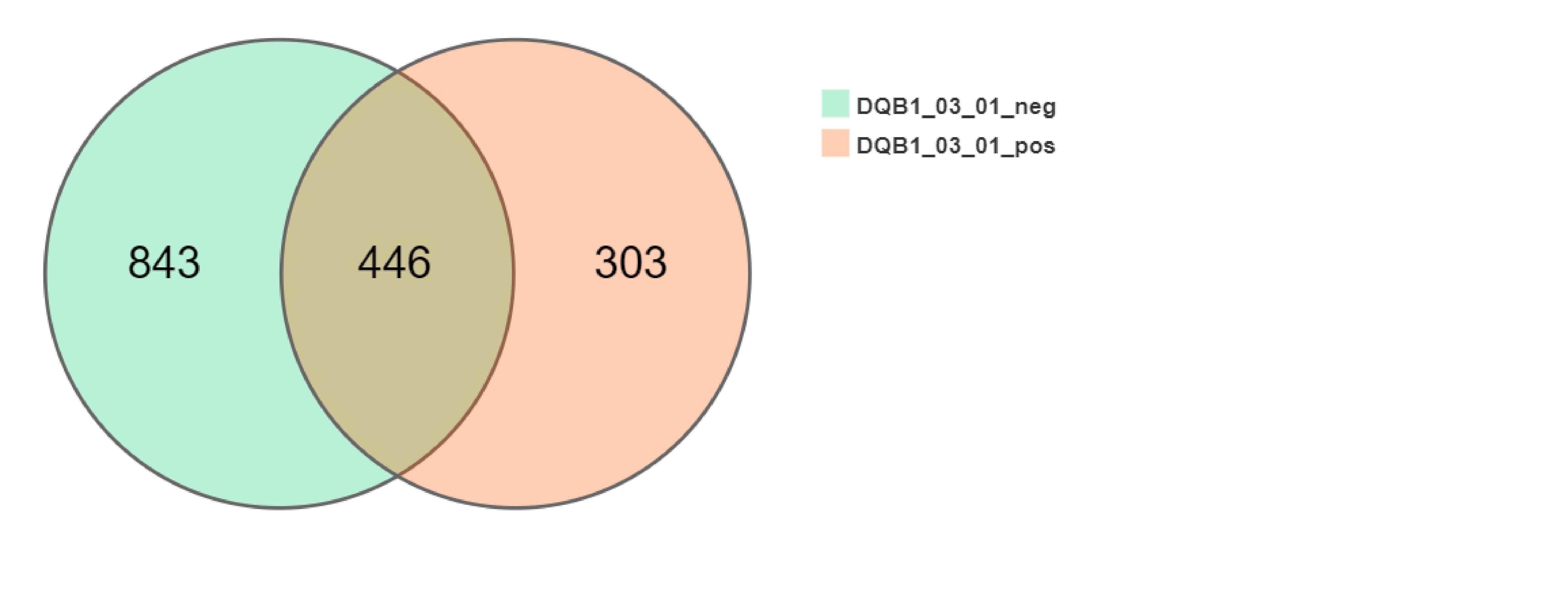

Supplement: Supplementary file 2 [file DataSheet_2.zip › Figure2-5/Figure3/Figure3A.jpg]

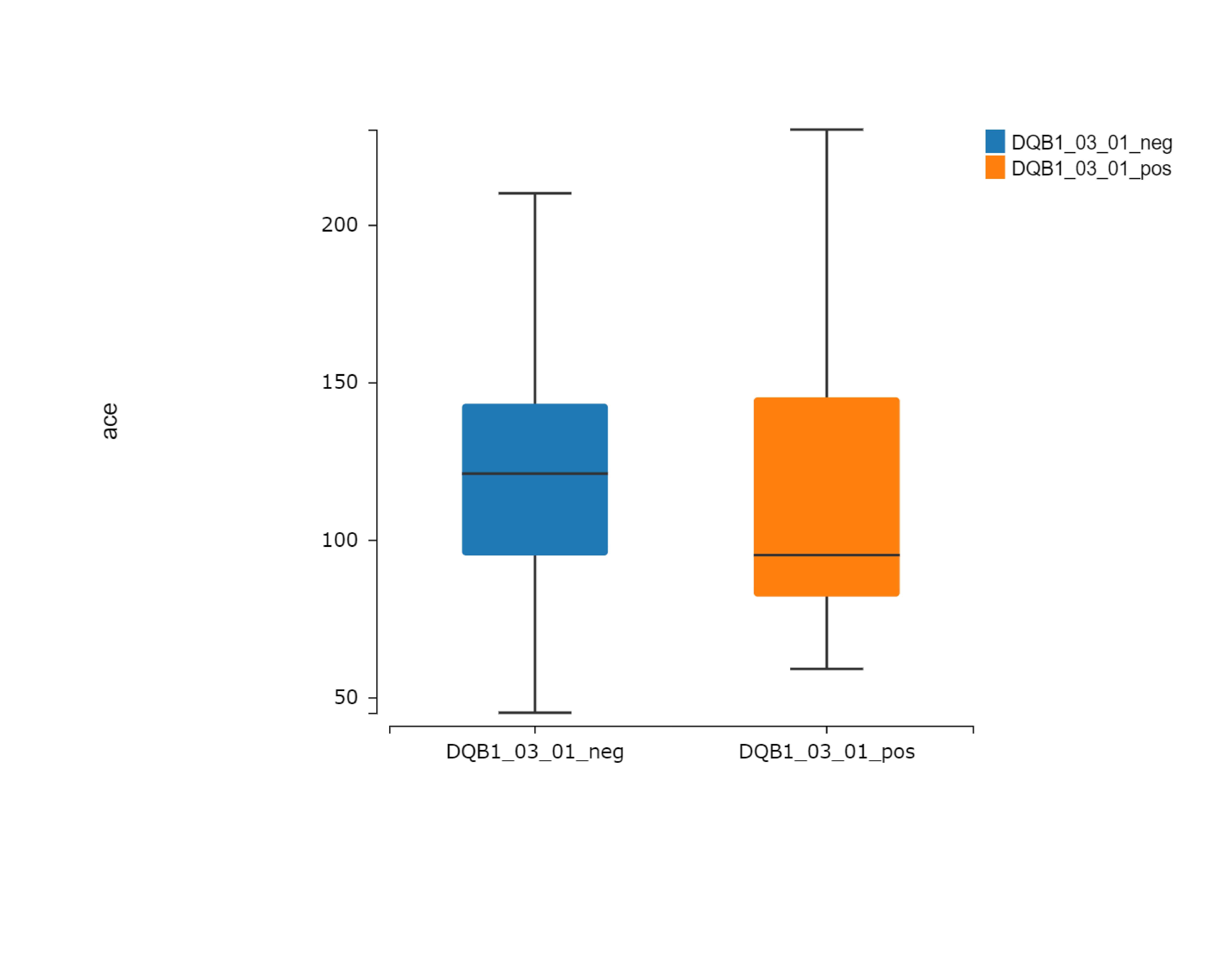

Supplement: Supplementary file 2 [file DataSheet_2.zip › Figure2-5/Figure3/Figure3B ace .jpg]

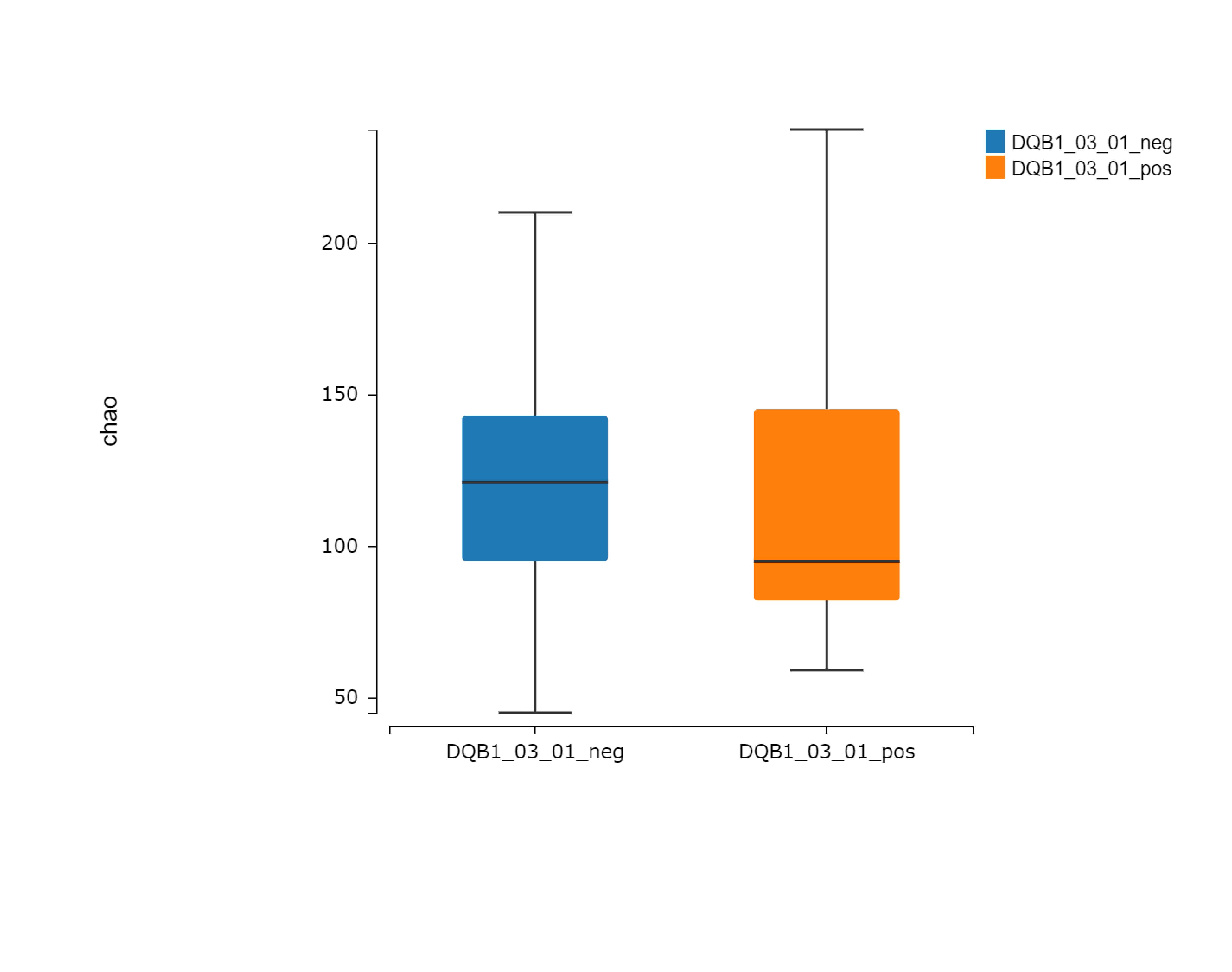

Supplement: Supplementary file 2 [file DataSheet_2.zip › Figure2-5/Figure3/Figure3B chao.jpg]

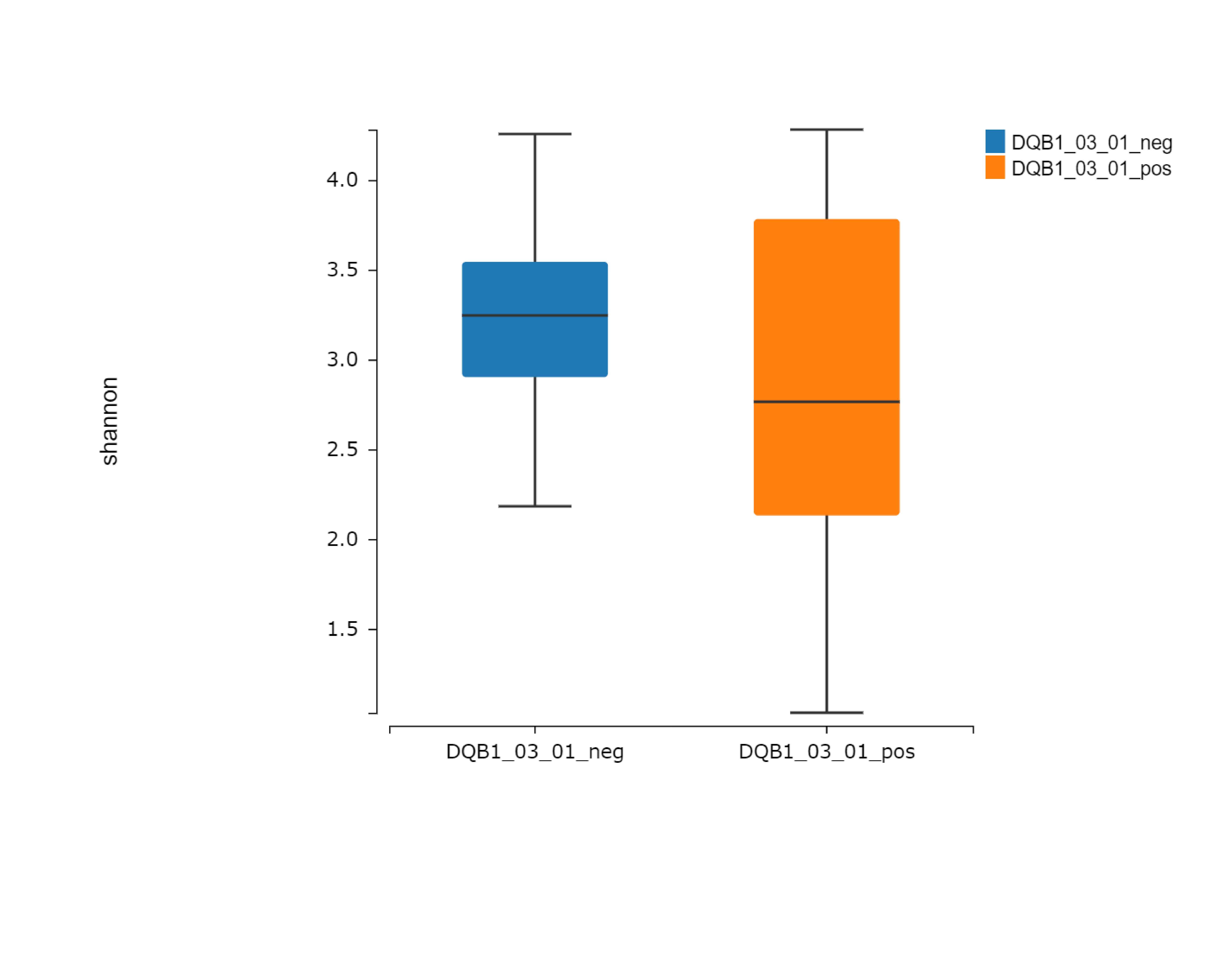

Supplement: Supplementary file 2 [file DataSheet_2.zip › Figure2-5/Figure3/Figure3B shannon.jpg]

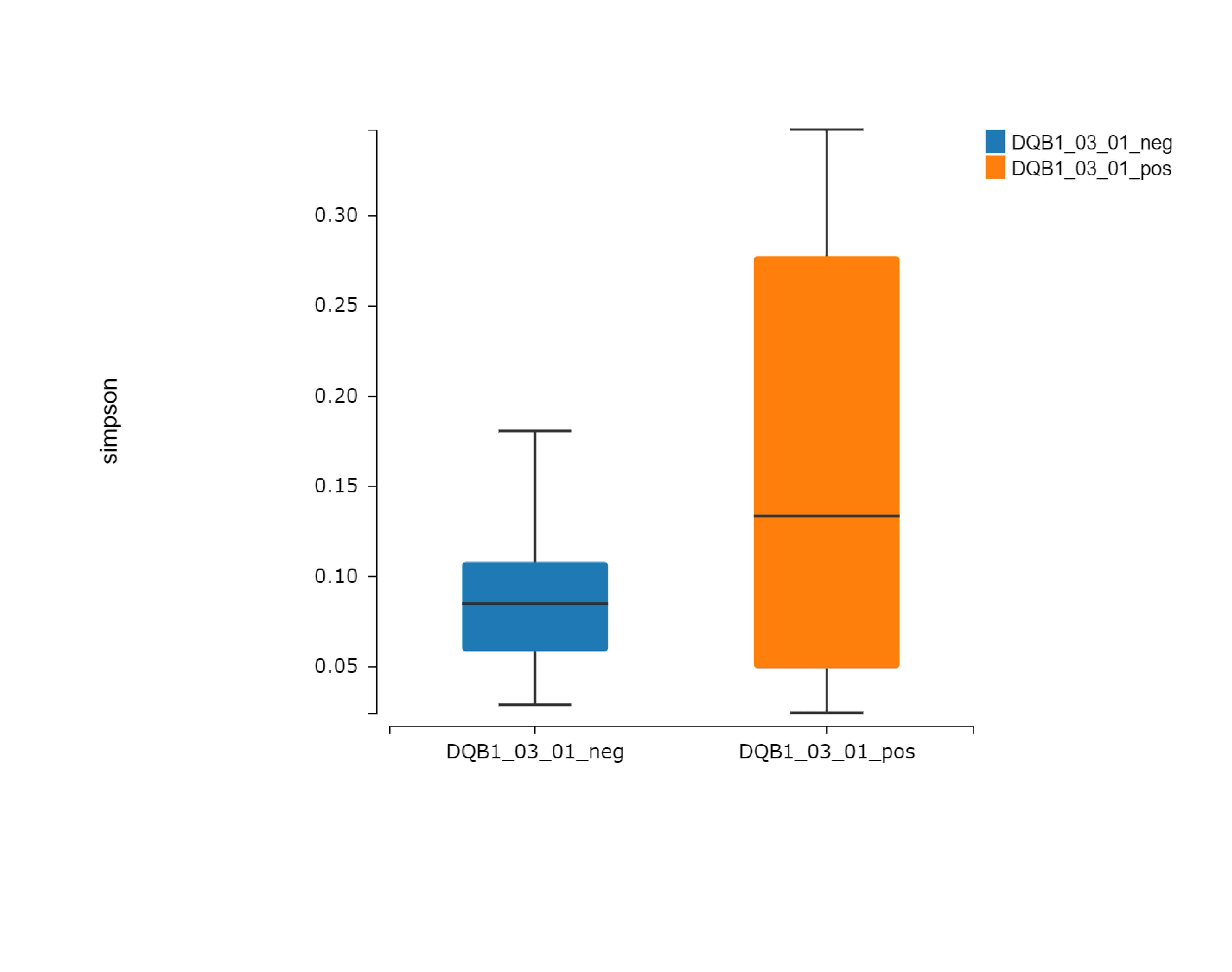

Supplement: Supplementary file 2 [file DataSheet_2.zip › Figure2-5/Figure3/Figure3B simpson.jpg]

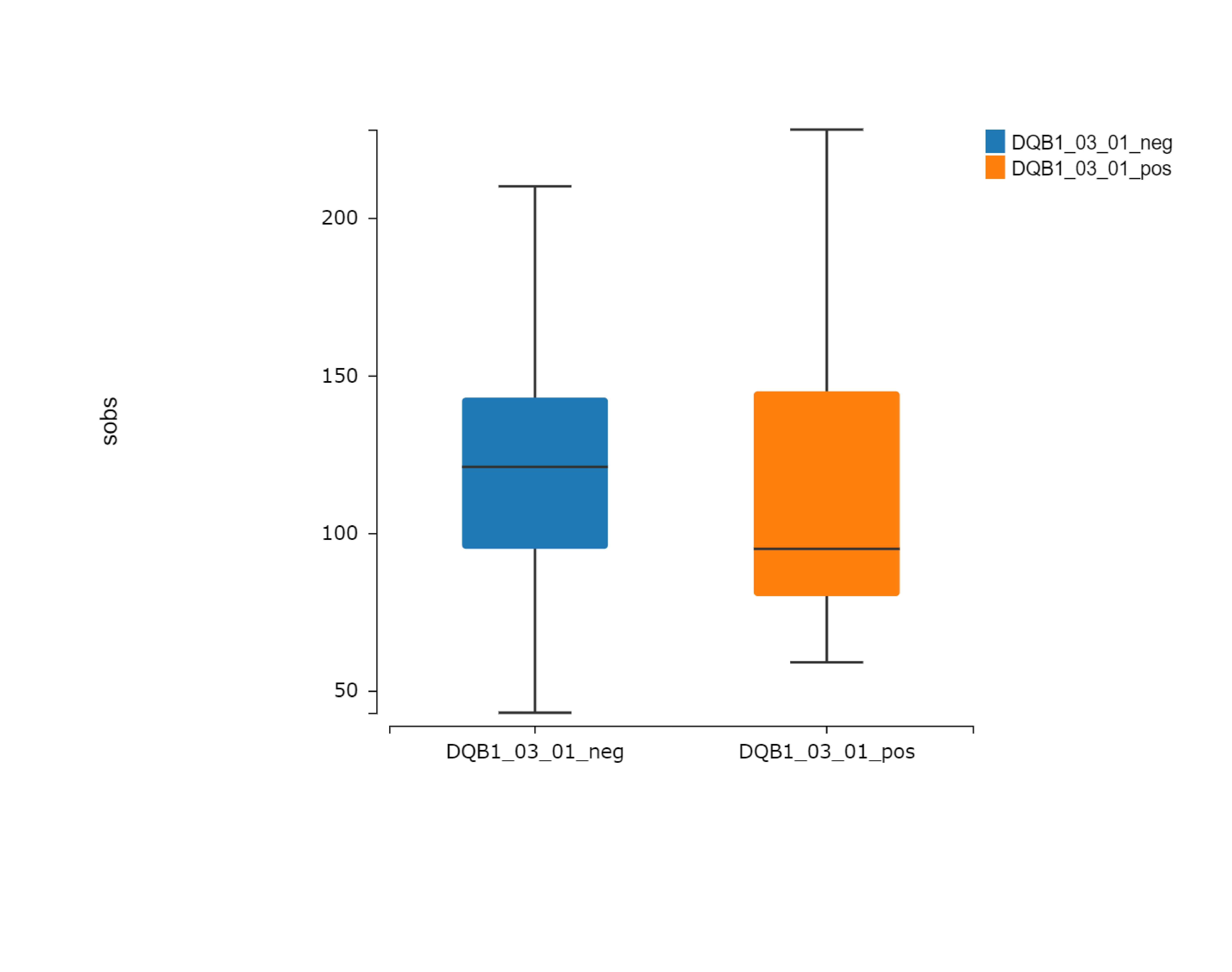

Supplement: Supplementary file 2 [file DataSheet_2.zip › Figure2-5/Figure3/Figure3B sobs.jpg]

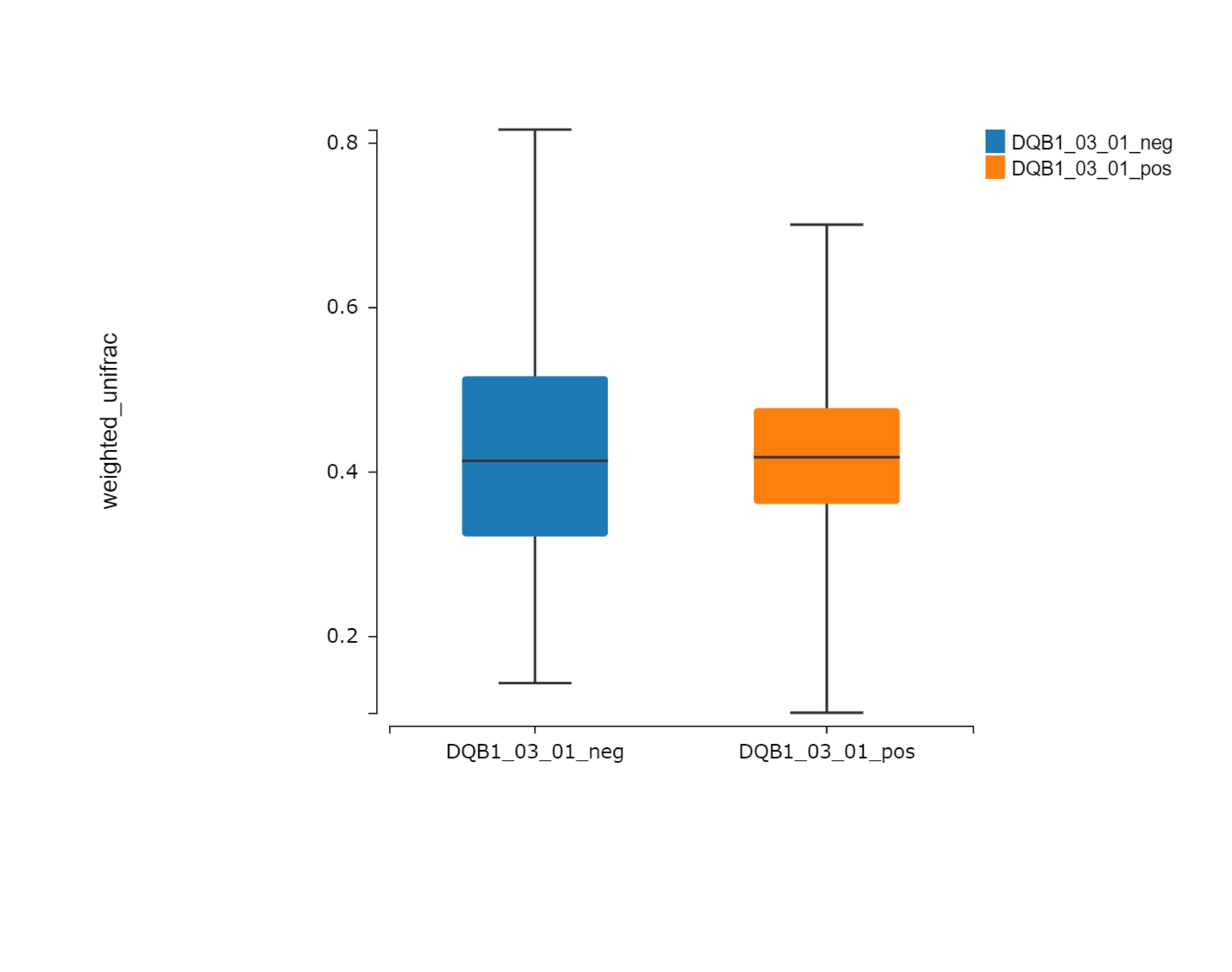

Supplement: Supplementary file 2 [file DataSheet_2.zip › Figure2-5/Figure3/Figure3C.jpg]

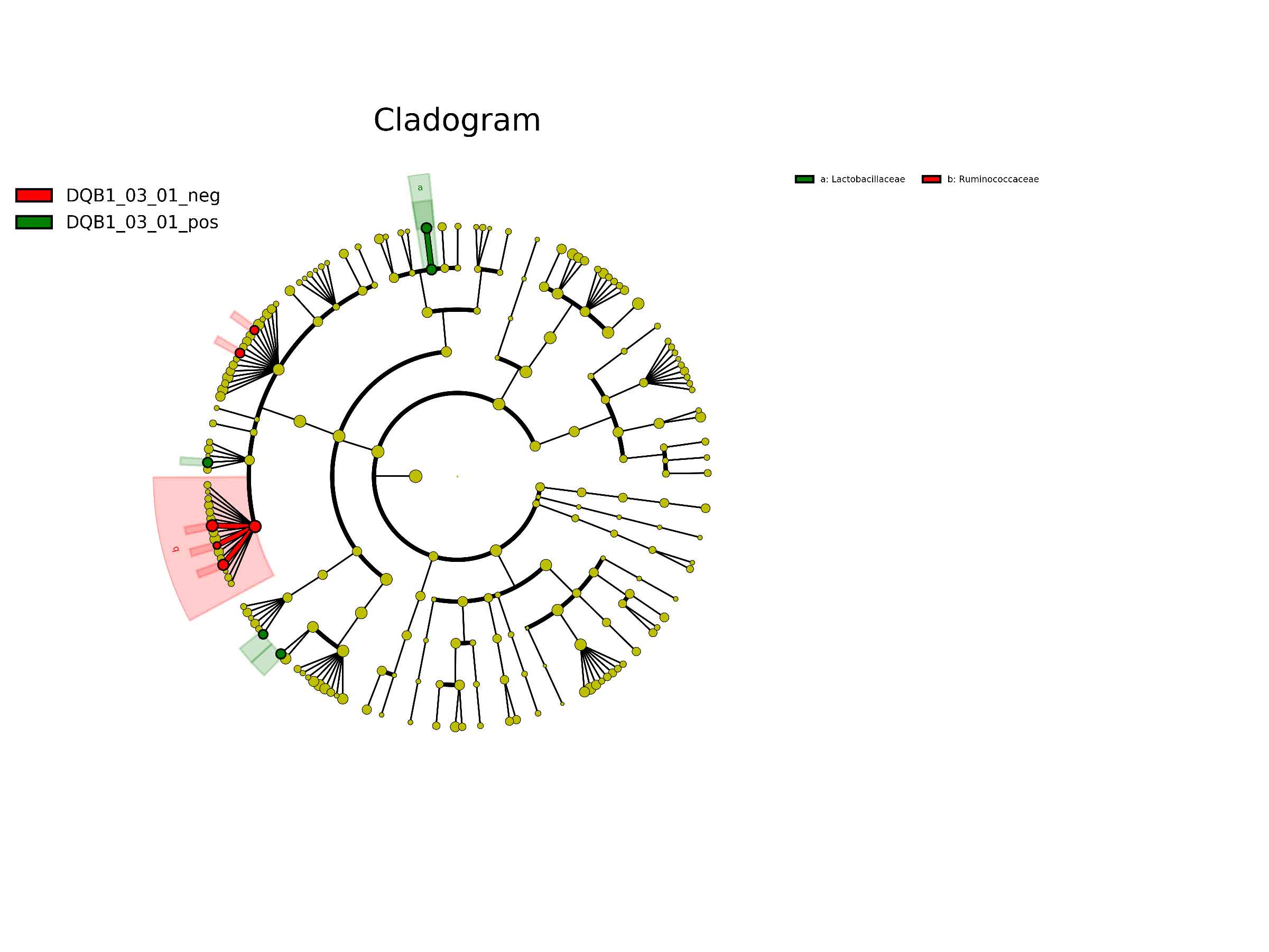

Supplement: Supplementary file 2 [file DataSheet_2.zip › Figure2-5/Figure3/Figure3D.jpg]

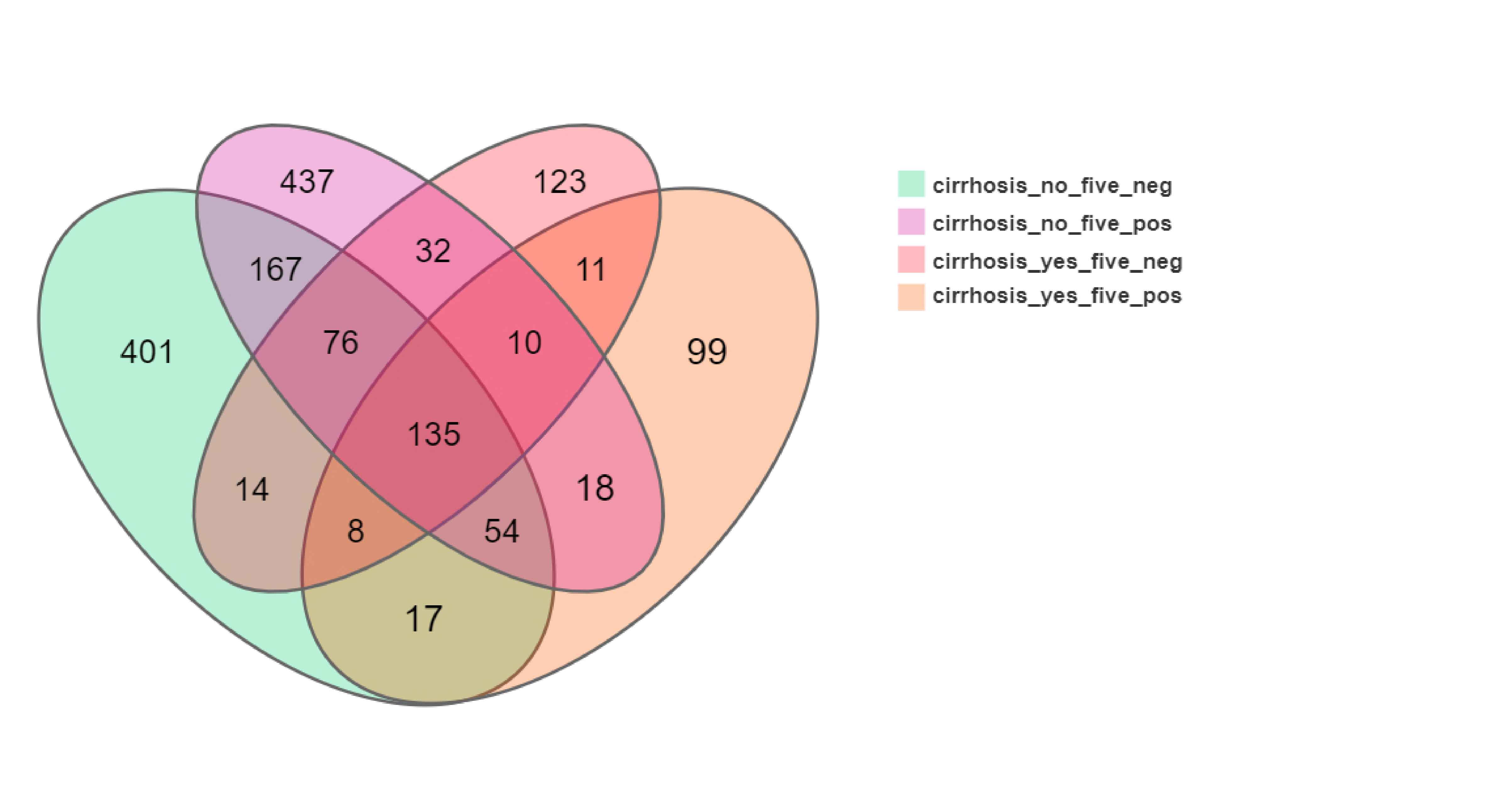

Supplement: Supplementary file 2 [file DataSheet_2.zip › Figure2-5/Figure4/Figure4A .jpg]

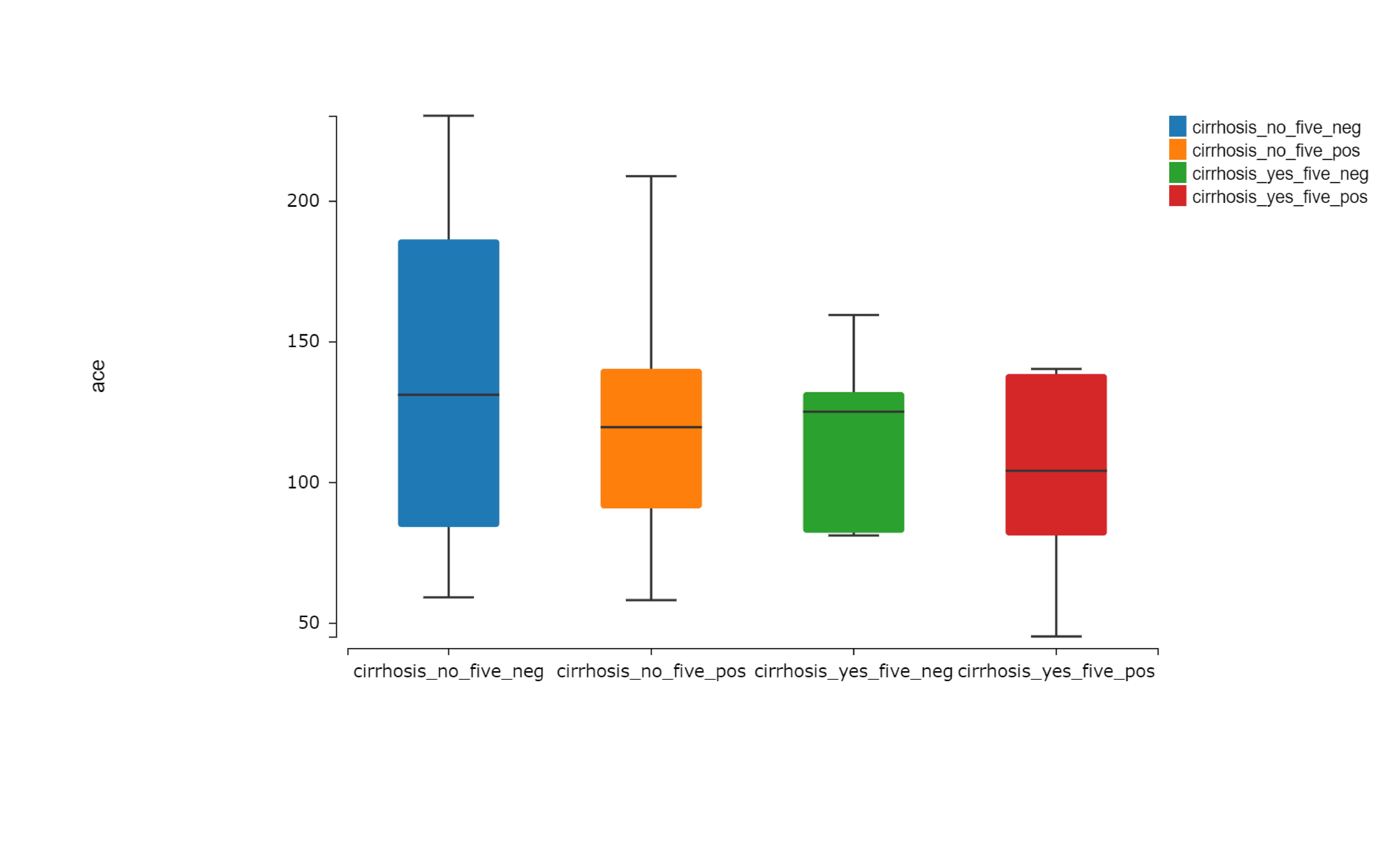

Supplement: Supplementary file 2 [file DataSheet_2.zip › Figure2-5/Figure4/Figure4B ace .jpg]

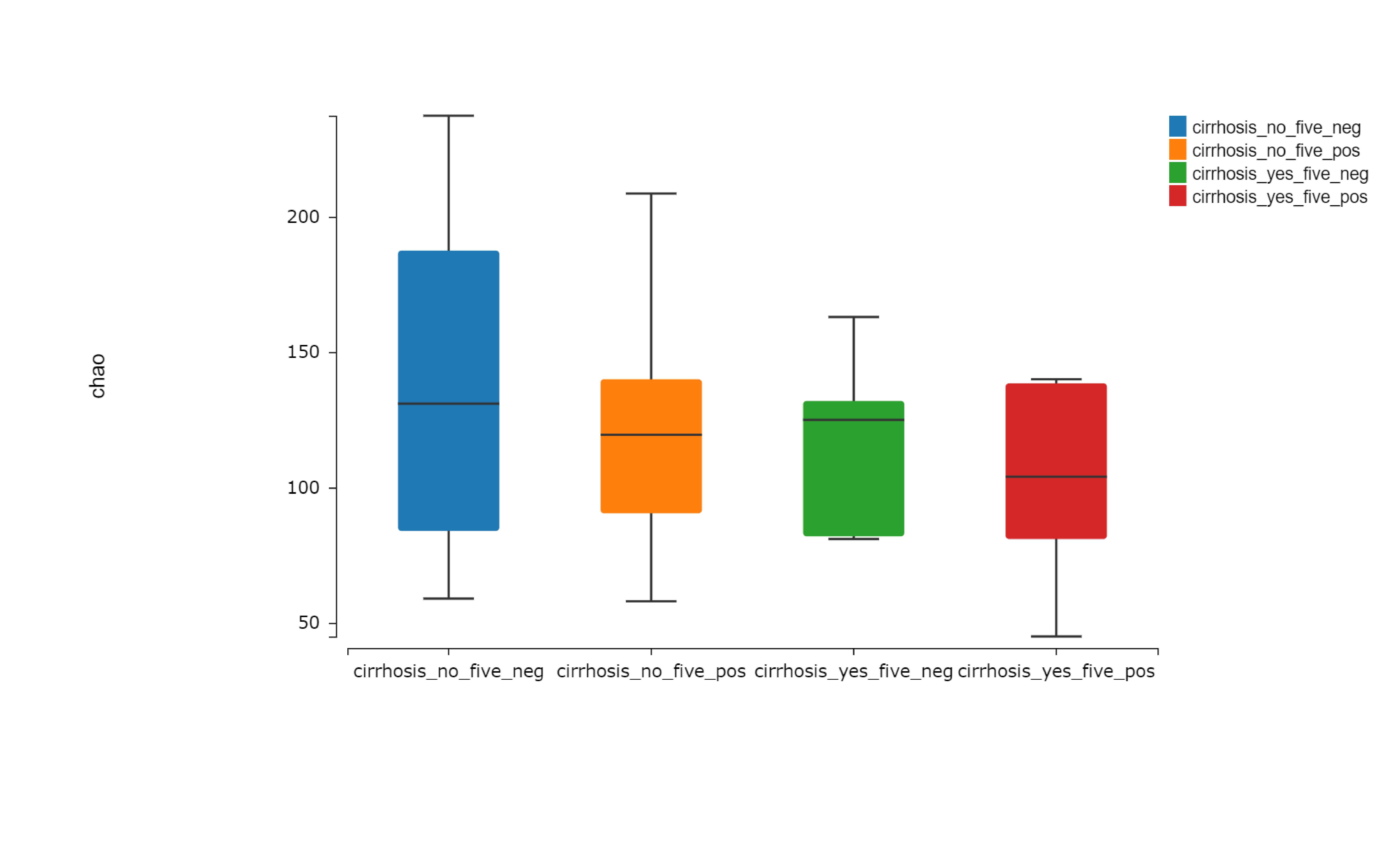

Supplement: Supplementary file 2 [file DataSheet_2.zip › Figure2-5/Figure4/Figure4B chao.jpg]

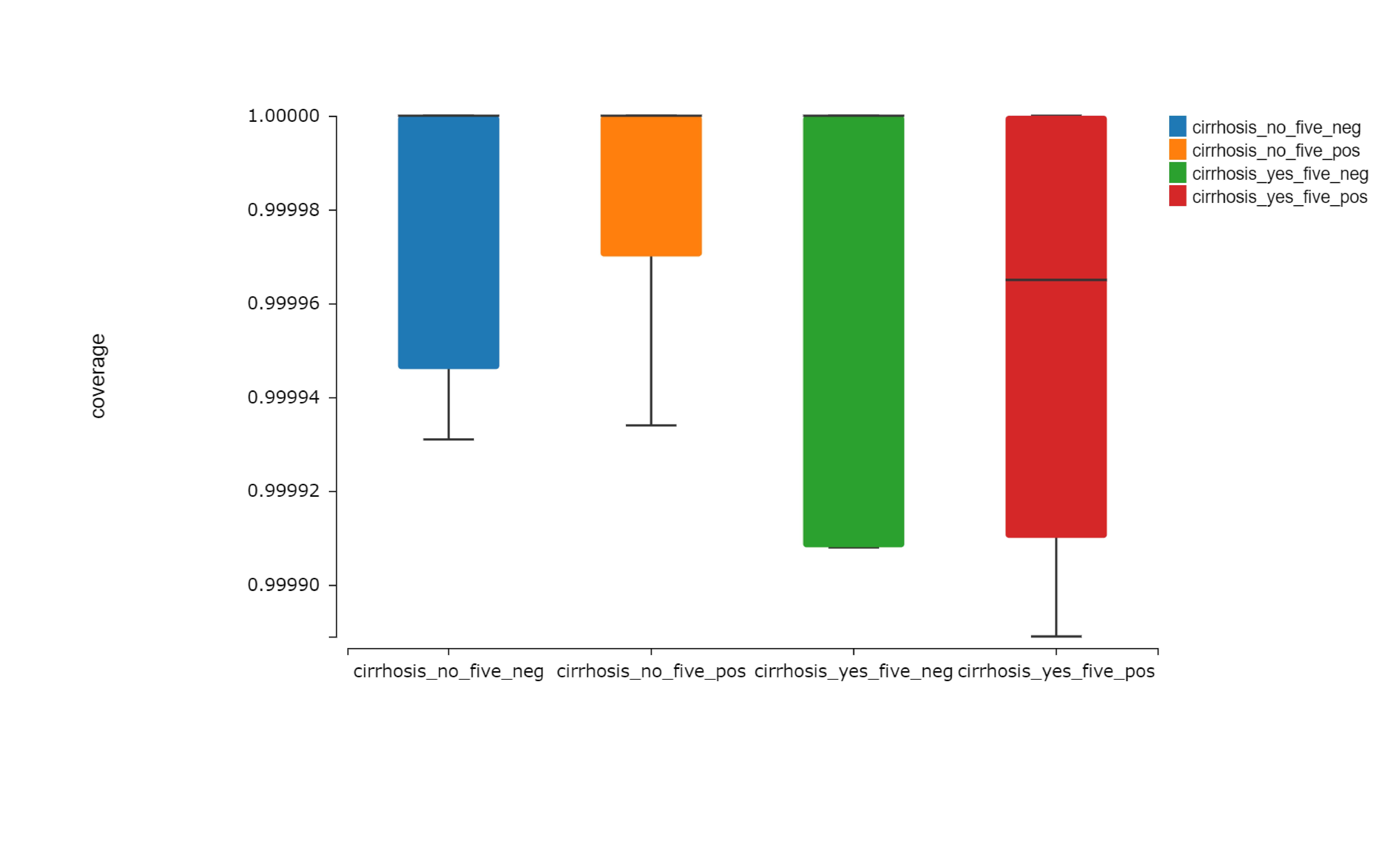

Supplement: Supplementary file 2 [file DataSheet_2.zip › Figure2-5/Figure4/Figure4B coverage.jpg]

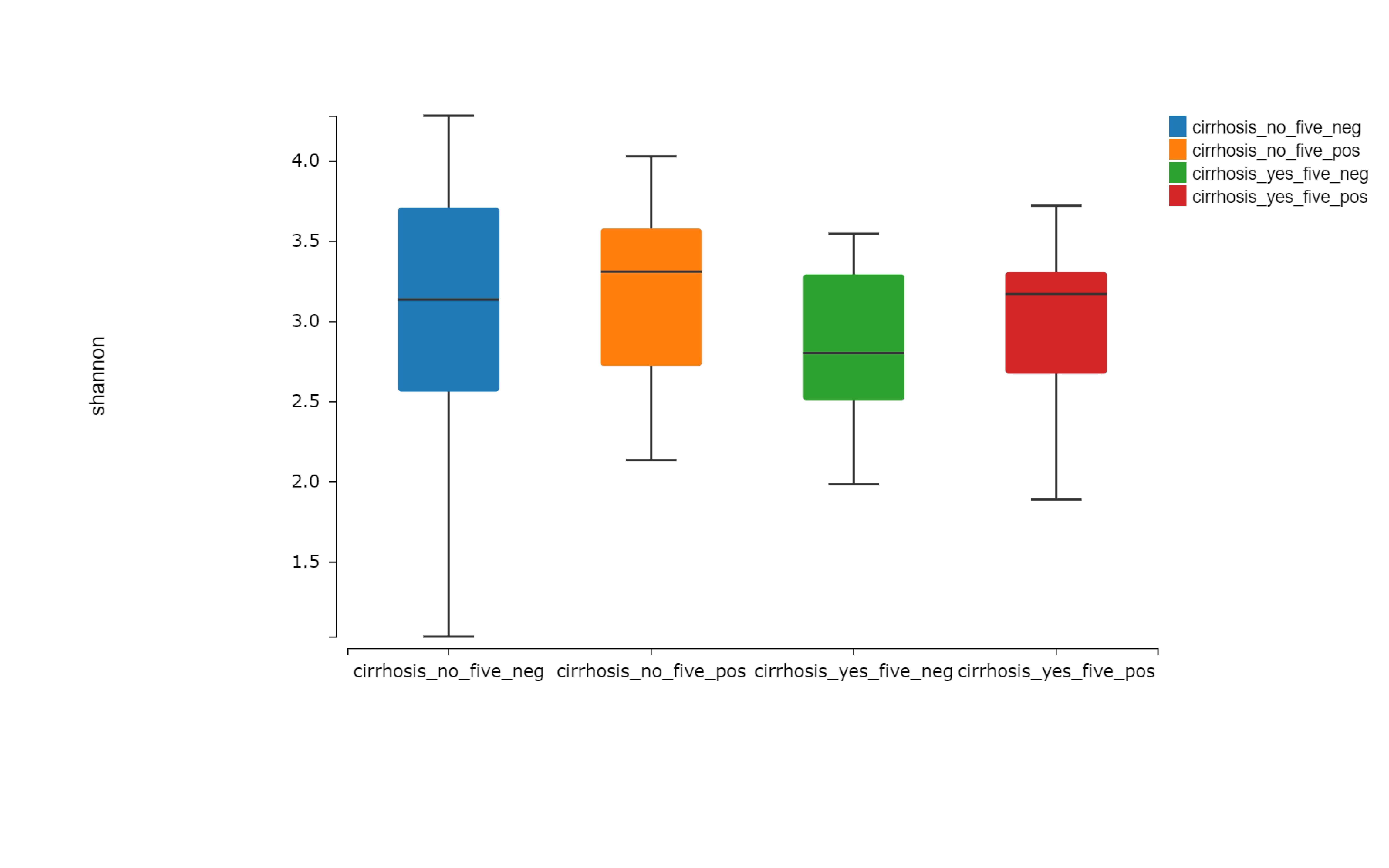

Supplement: Supplementary file 2 [file DataSheet_2.zip › Figure2-5/Figure4/Figure4B shannon.jpg]

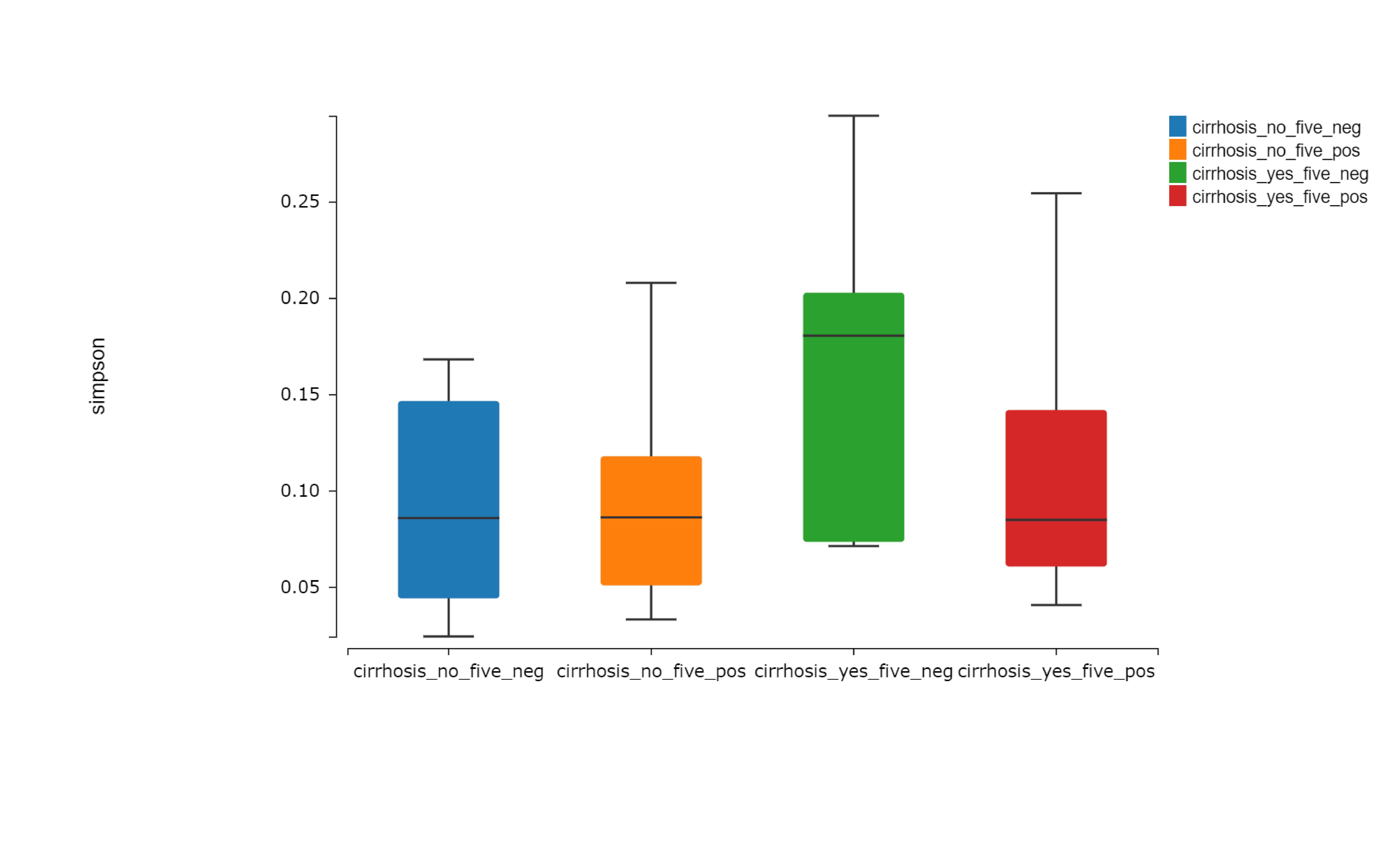

Supplement: Supplementary file 2 [file DataSheet_2.zip › Figure2-5/Figure4/Figure4B simpson.jpg]

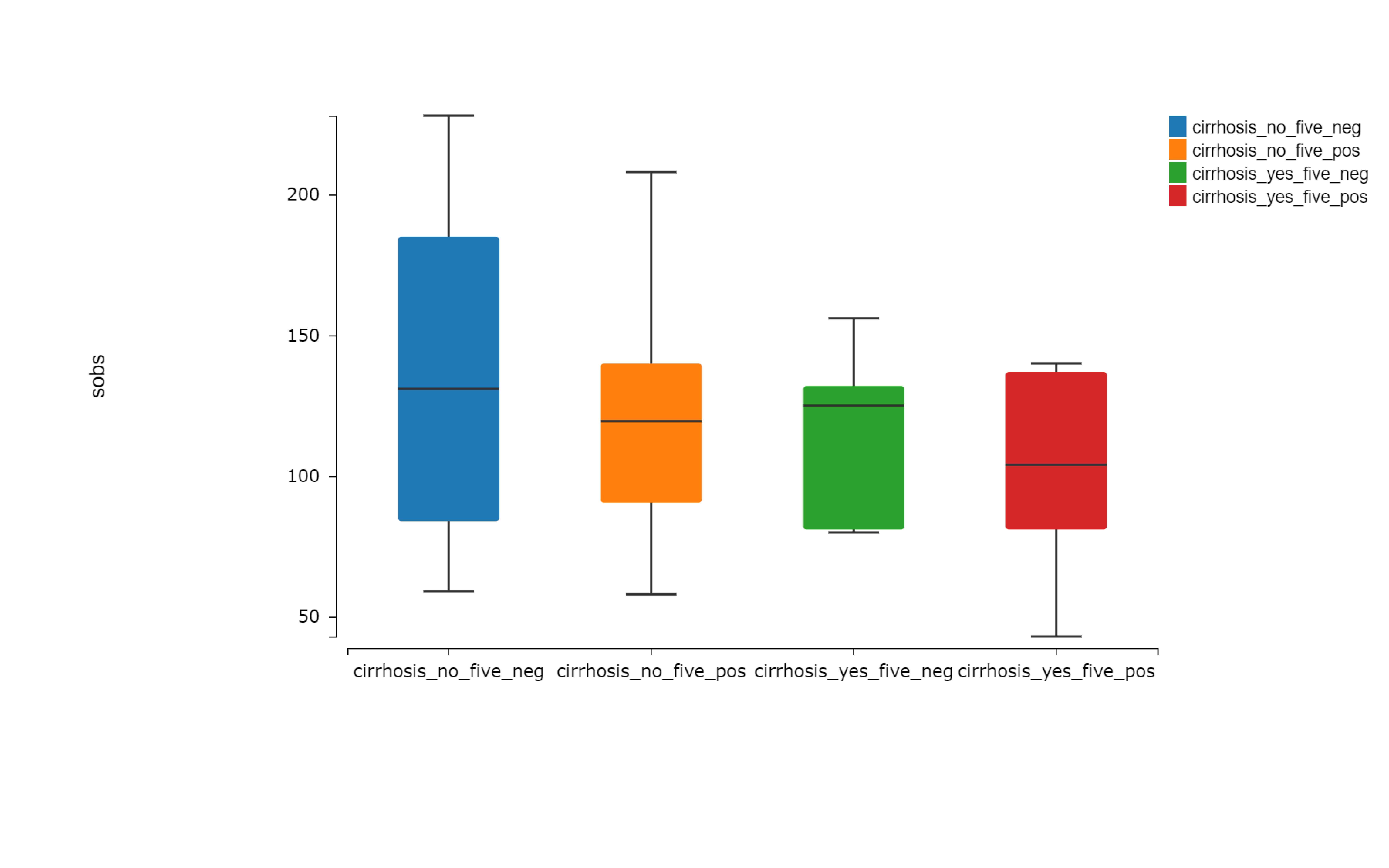

Supplement: Supplementary file 2 [file DataSheet_2.zip › Figure2-5/Figure4/Figure4B sobs.jpg]

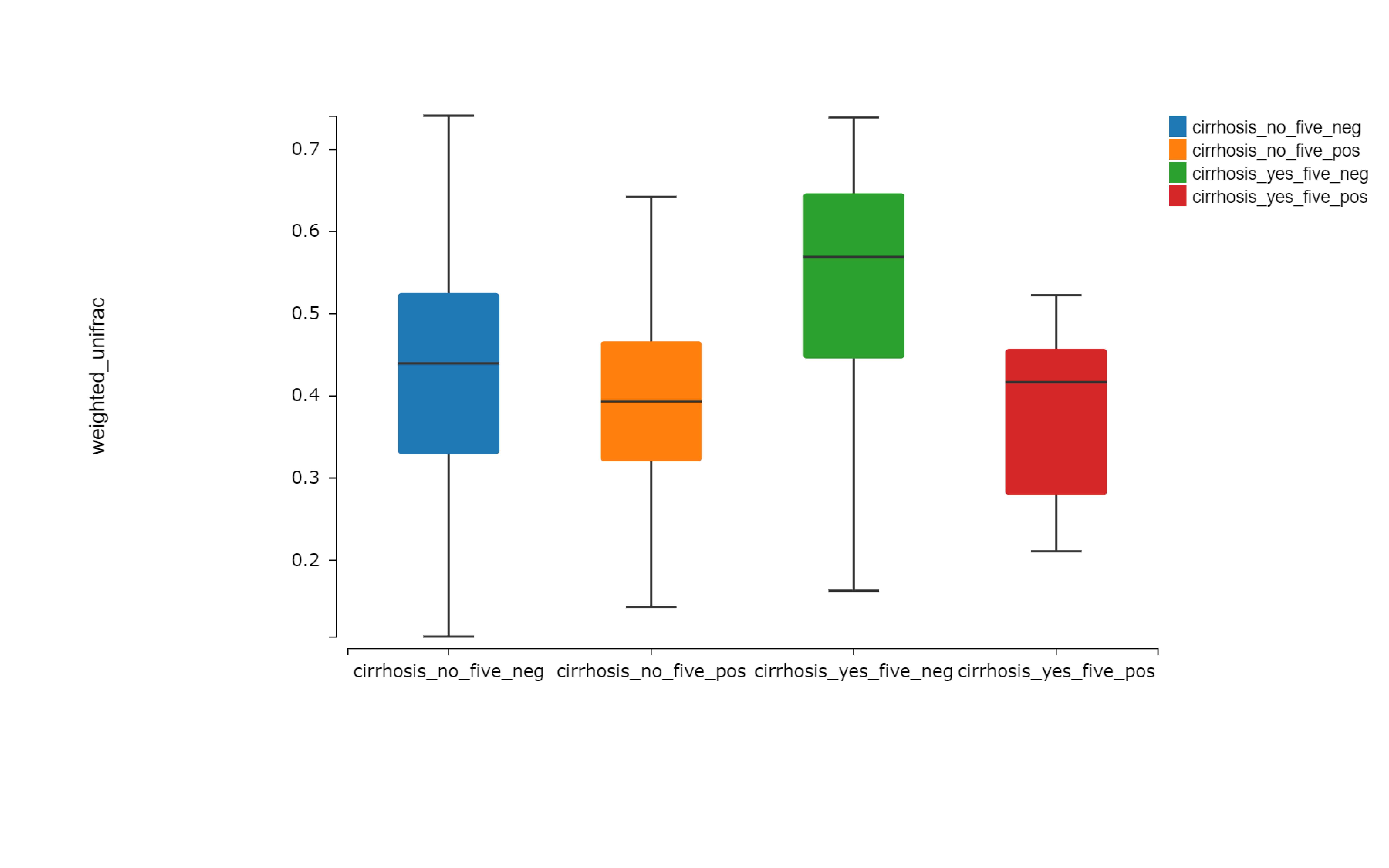

Supplement: Supplementary file 2 [file DataSheet_2.zip › Figure2-5/Figure4/Figure4C .jpg]

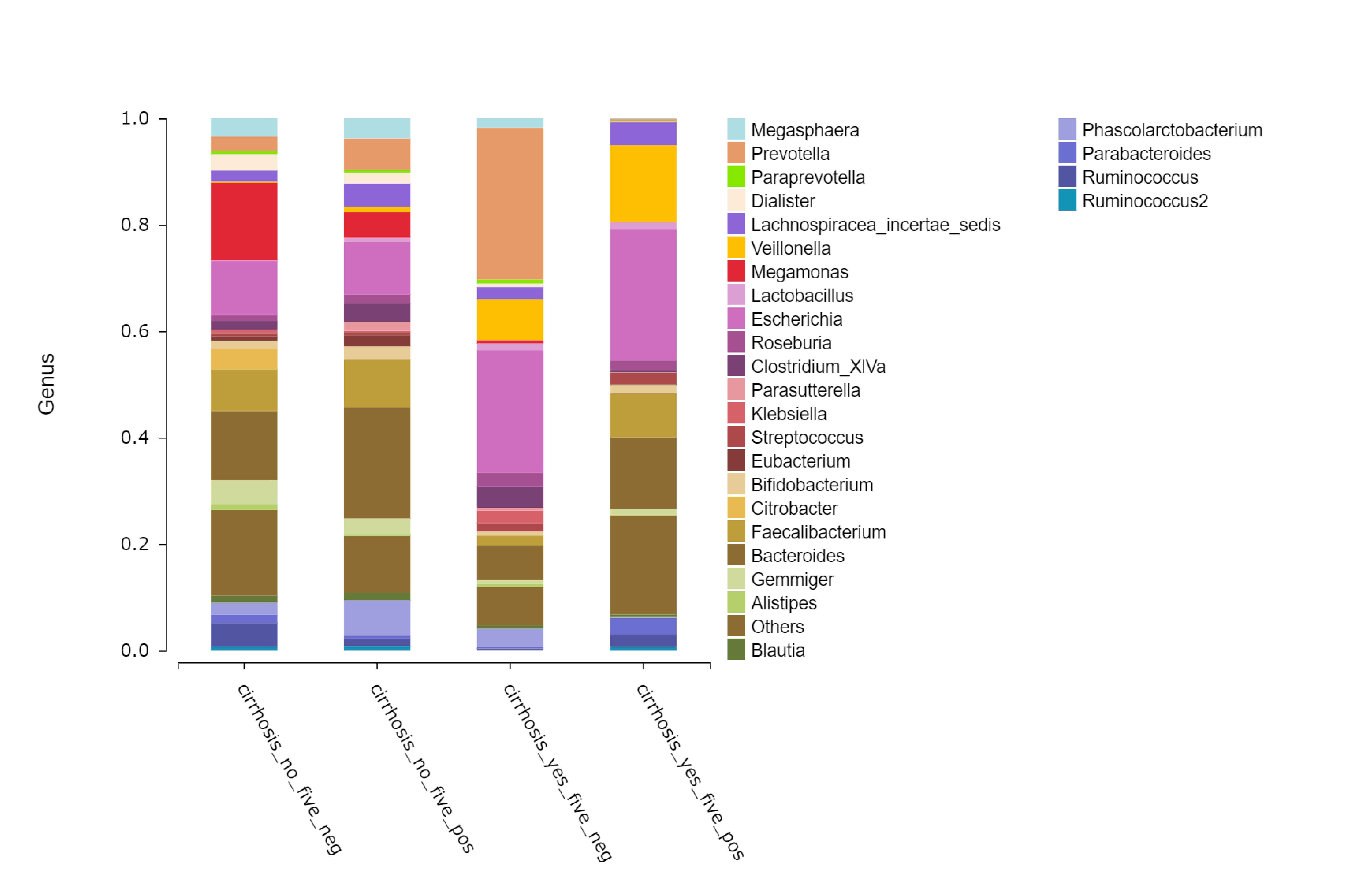

Supplement: Supplementary file 2 [file DataSheet_2.zip › Figure2-5/Figure4/Figure4D .jpg]

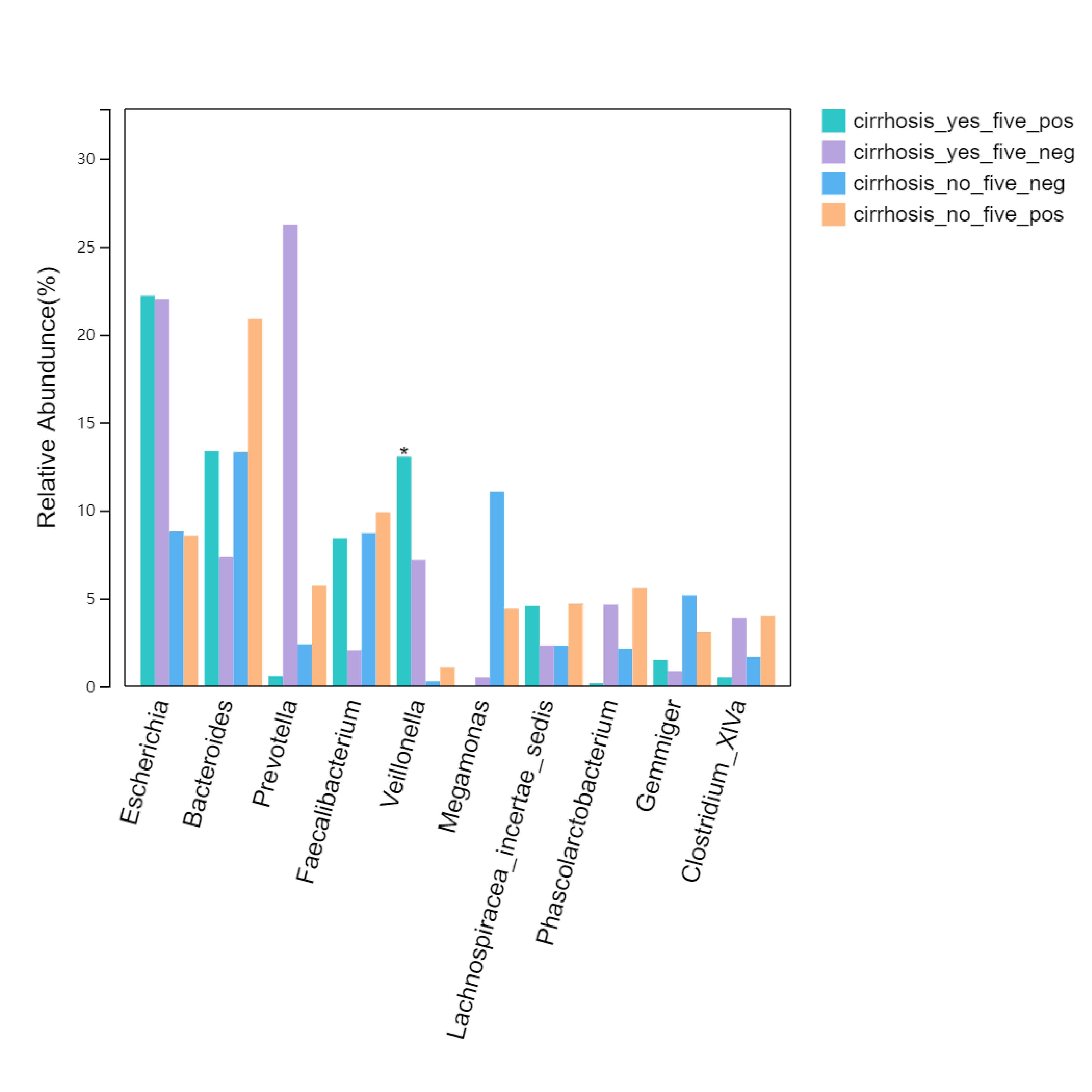

Supplement: Supplementary file 2 [file DataSheet_2.zip › Figure2-5/Figure4/Figure4E.jpg]

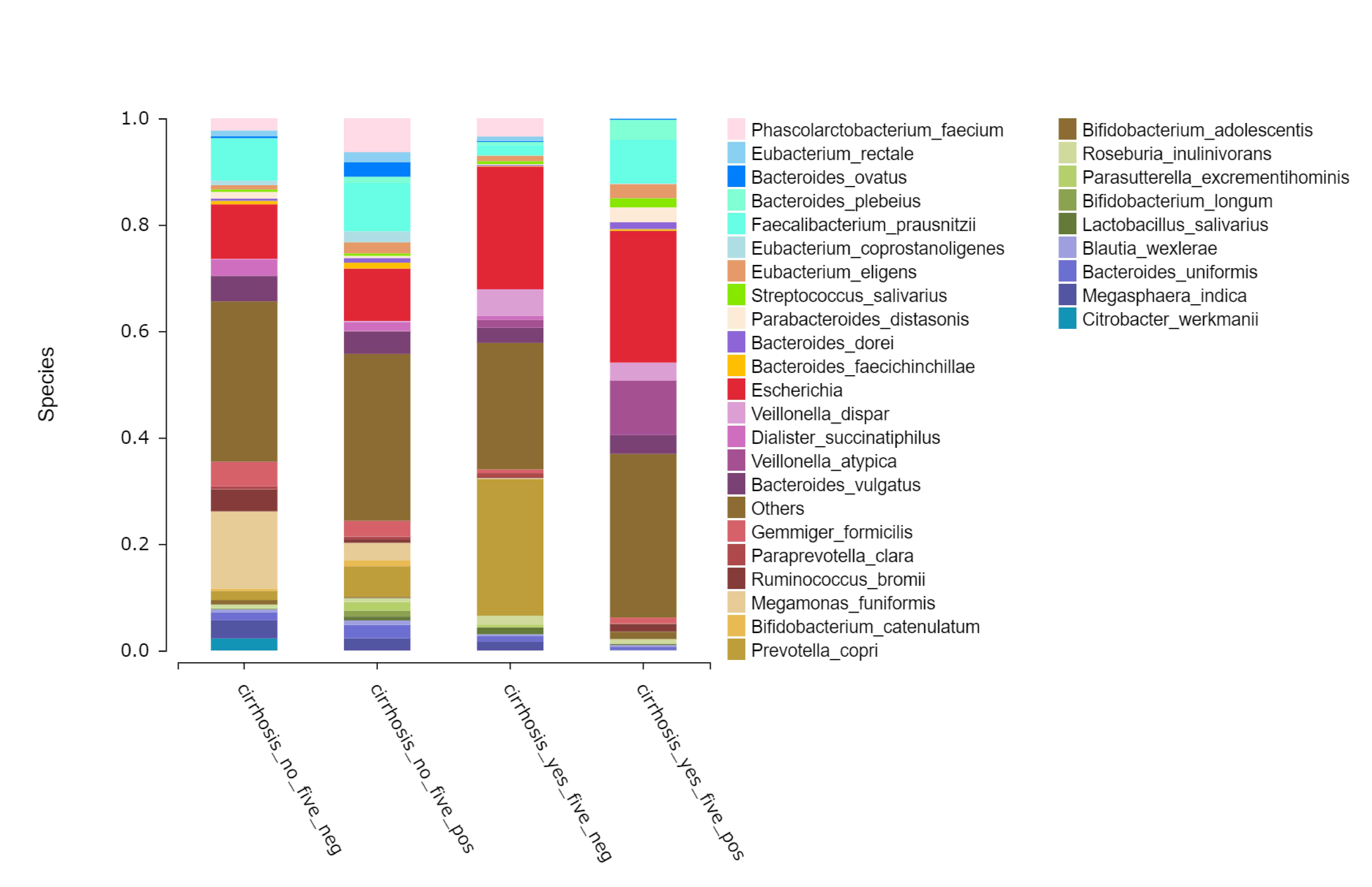

Supplement: Supplementary file 2 [file DataSheet_2.zip › Figure2-5/Figure4/Figure4F .jpg]

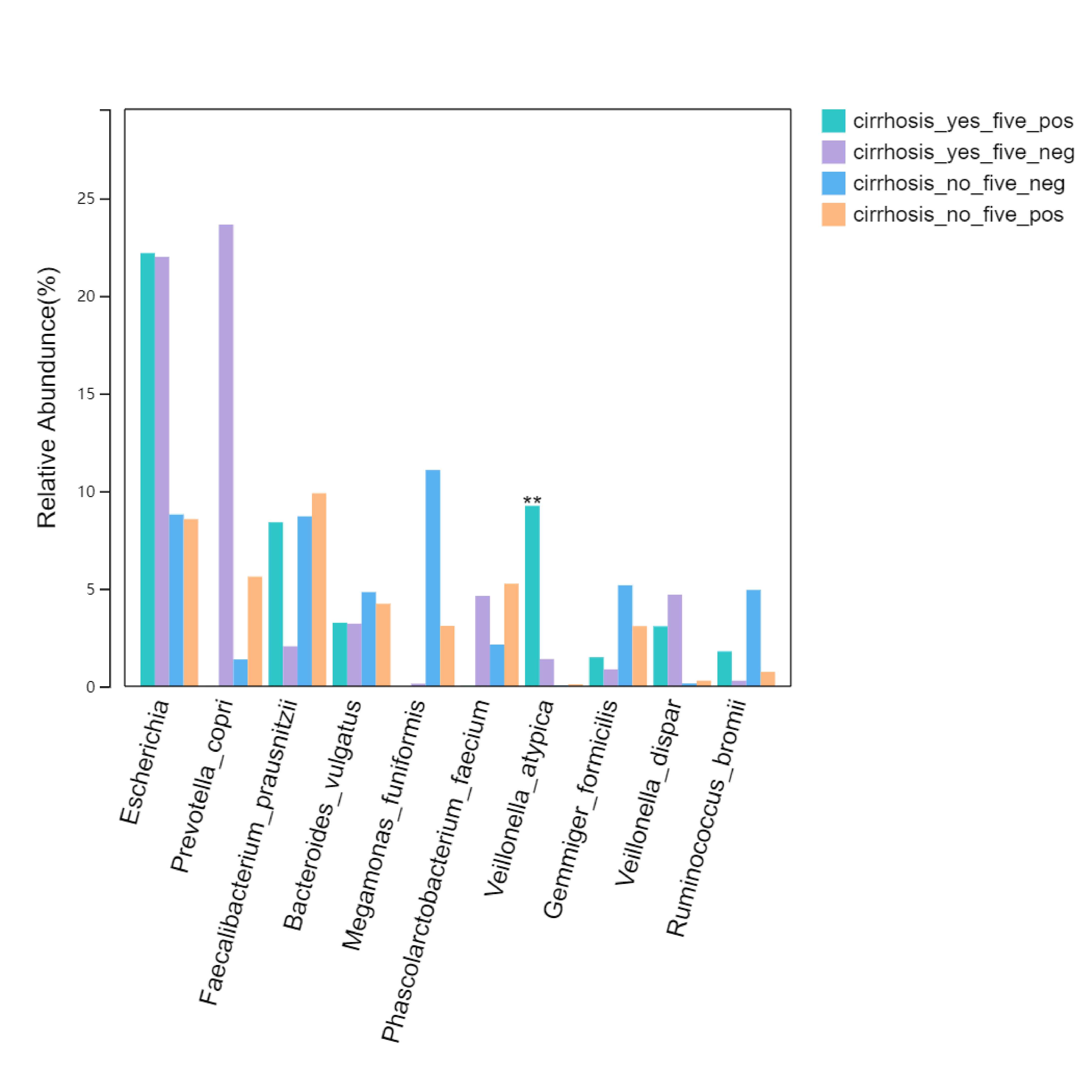

Supplement: Supplementary file 2 [file DataSheet_2.zip › Figure2-5/Figure4/Figure4G.jpg]

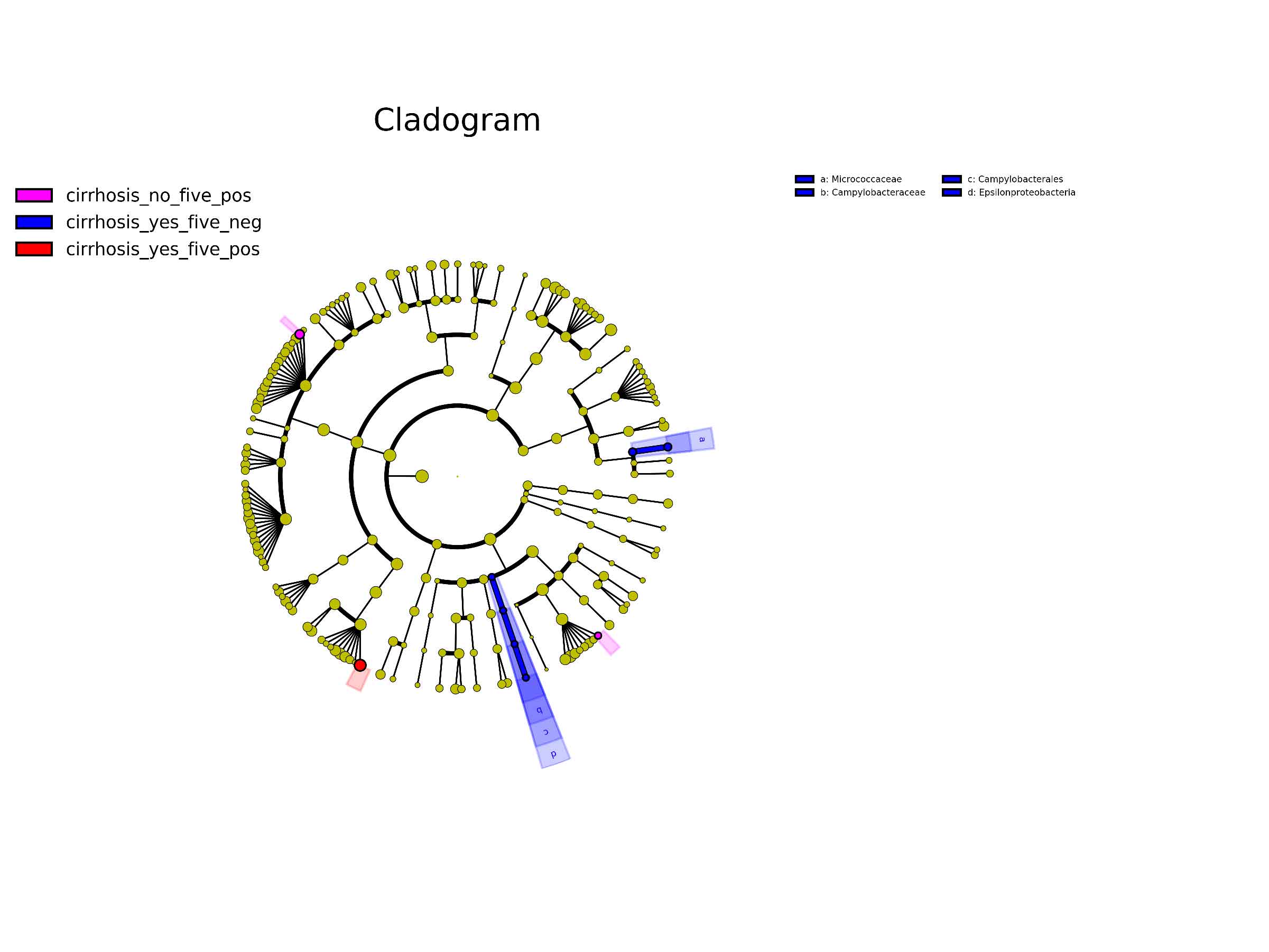

Supplement: Supplementary file 2 [file DataSheet_2.zip › Figure2-5/Figure4/Figure4H .jpg]

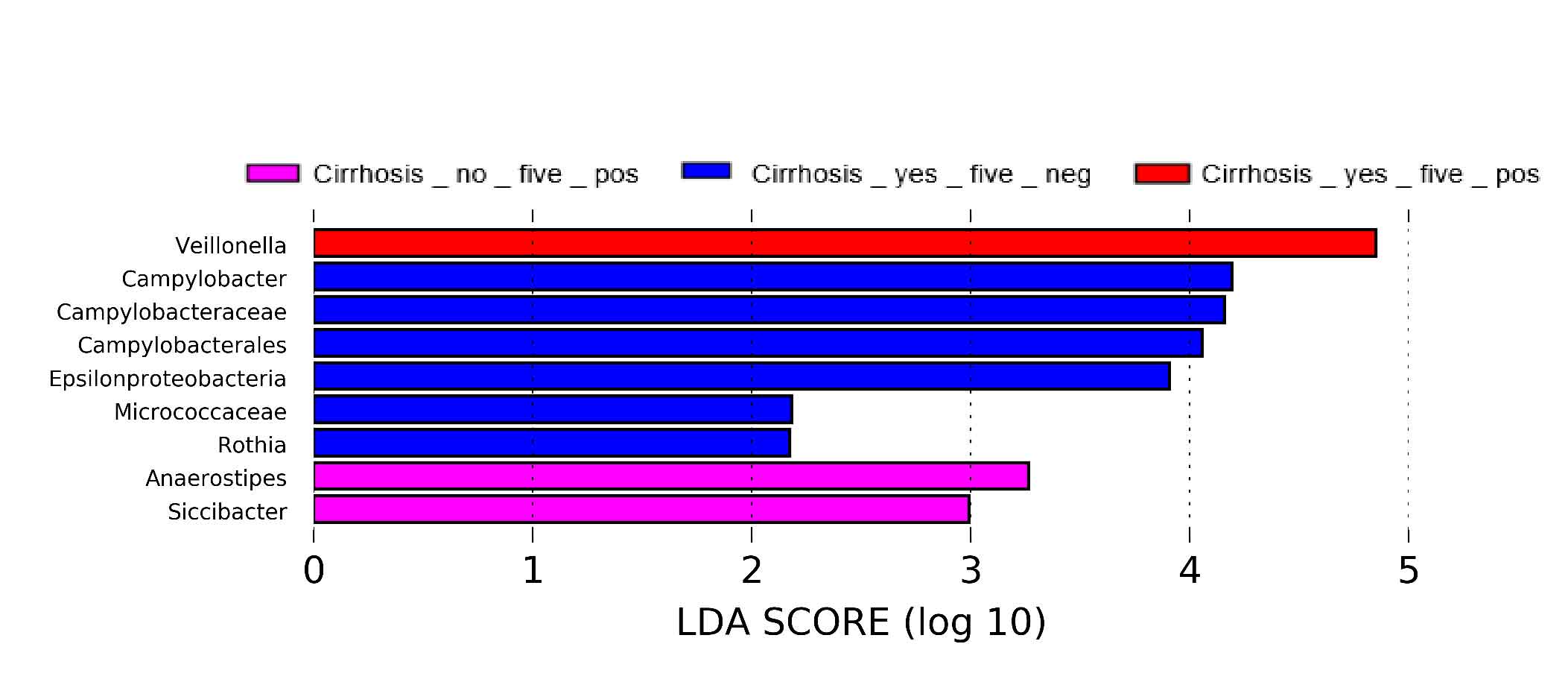

Supplement: Supplementary file 2 [file DataSheet_2.zip › Figure2-5/Figure4/Figure4I.jpg]

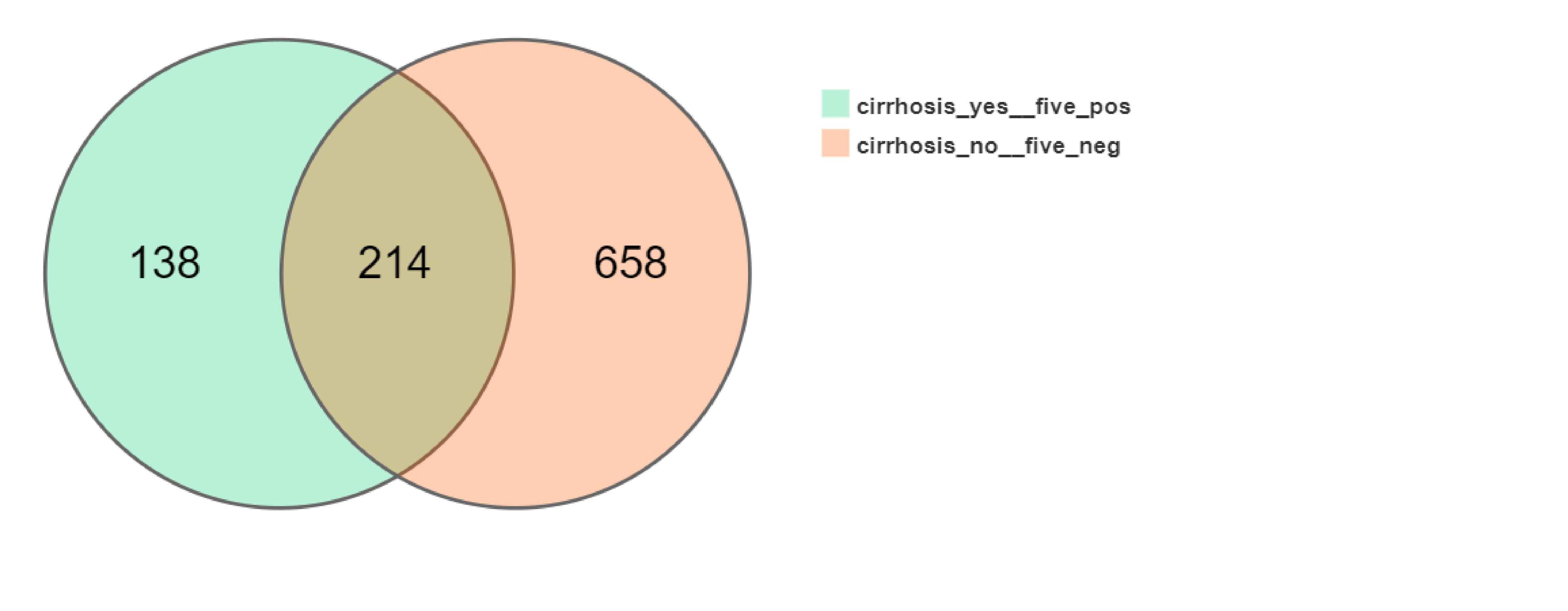

Supplement: Supplementary file 2 [file DataSheet_2.zip › Figure2-5/Figure5/Figure5A .jpg]

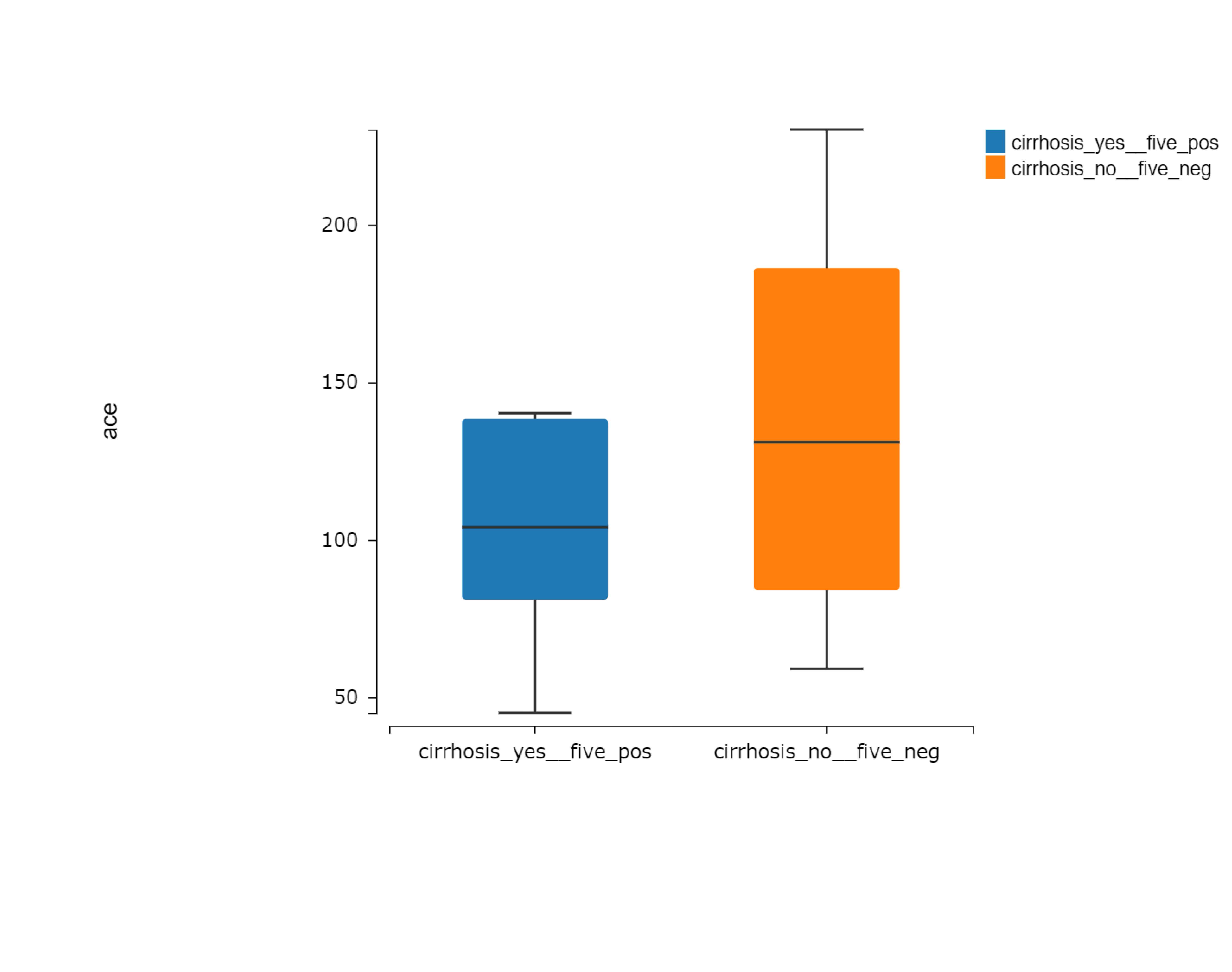

Supplement: Supplementary file 2 [file DataSheet_2.zip › Figure2-5/Figure5/Figure5B ace.jpg]

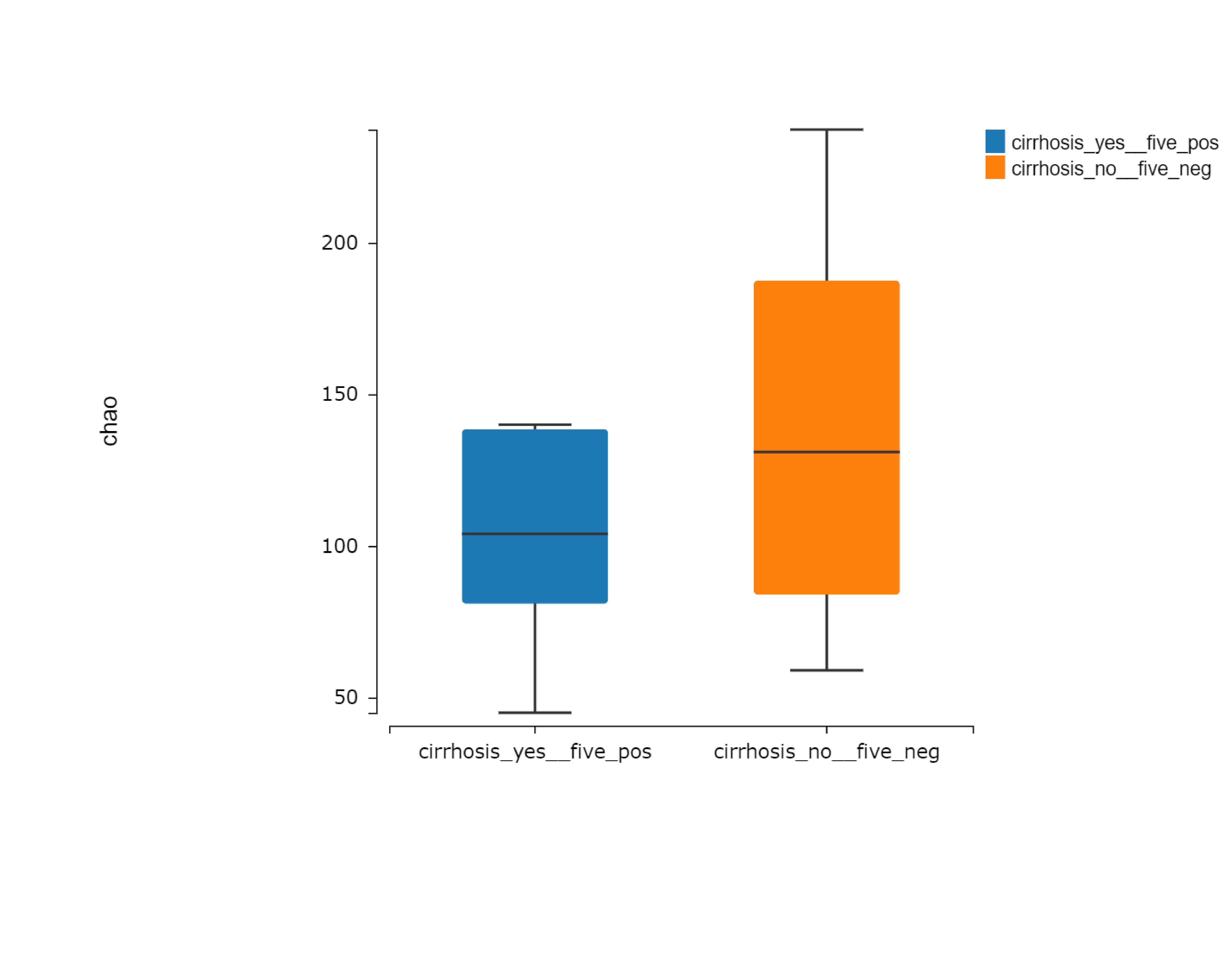

Supplement: Supplementary file 2 [file DataSheet_2.zip › Figure2-5/Figure5/Figure5B chao.jpg]

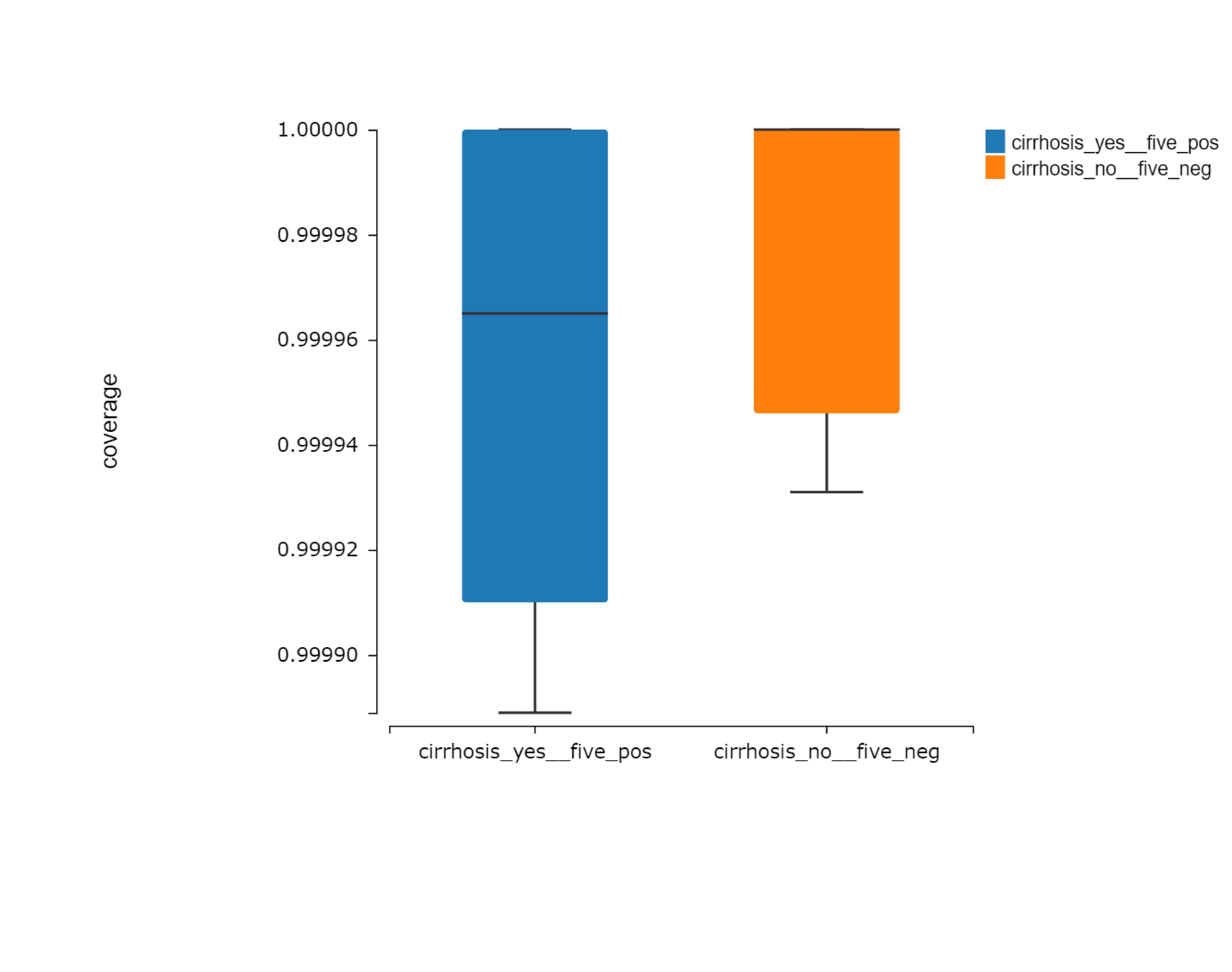

Supplement: Supplementary file 2 [file DataSheet_2.zip › Figure2-5/Figure5/Figure5B coverage.jpg]

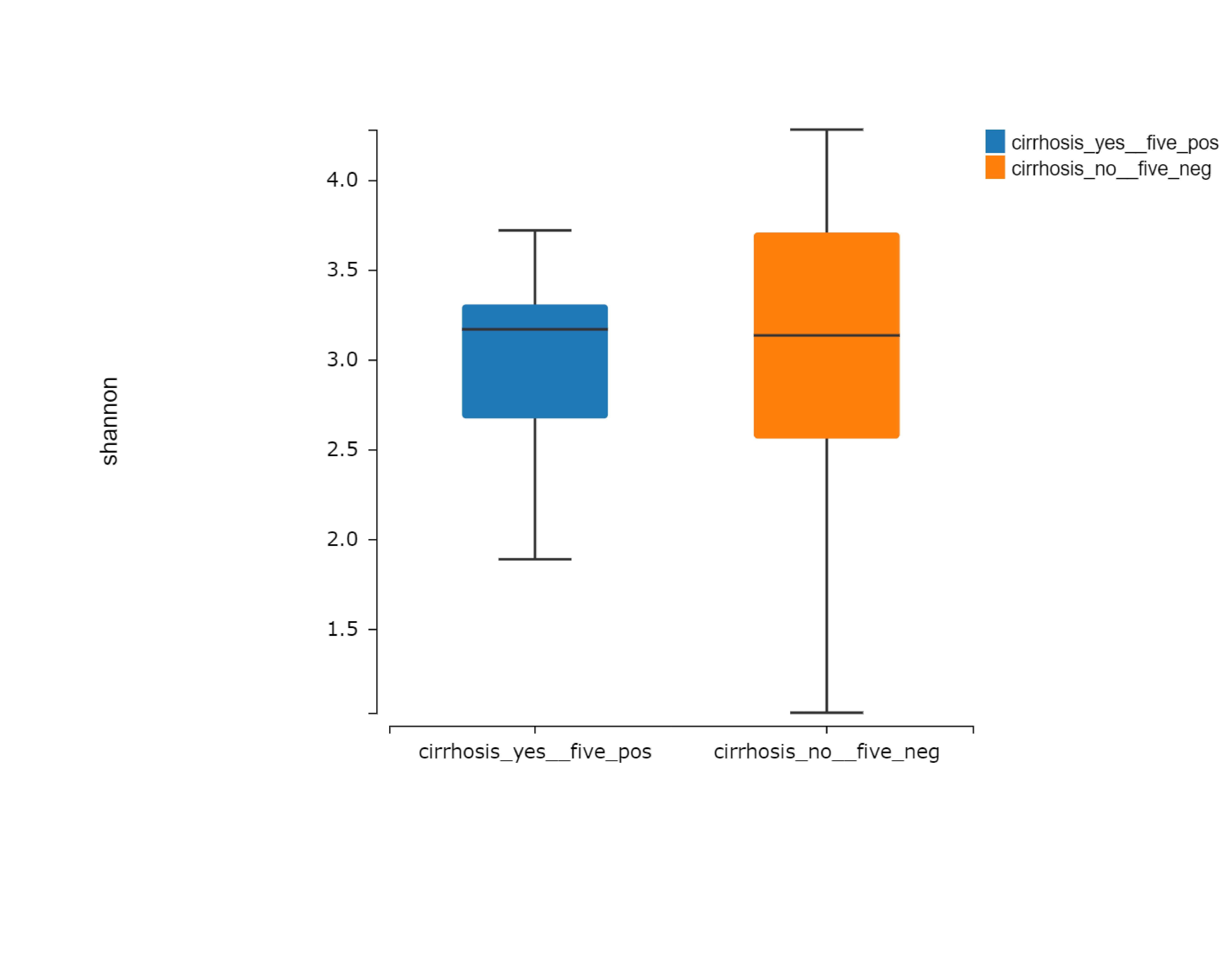

Supplement: Supplementary file 2 [file DataSheet_2.zip › Figure2-5/Figure5/Figure5B shannon.jpg]

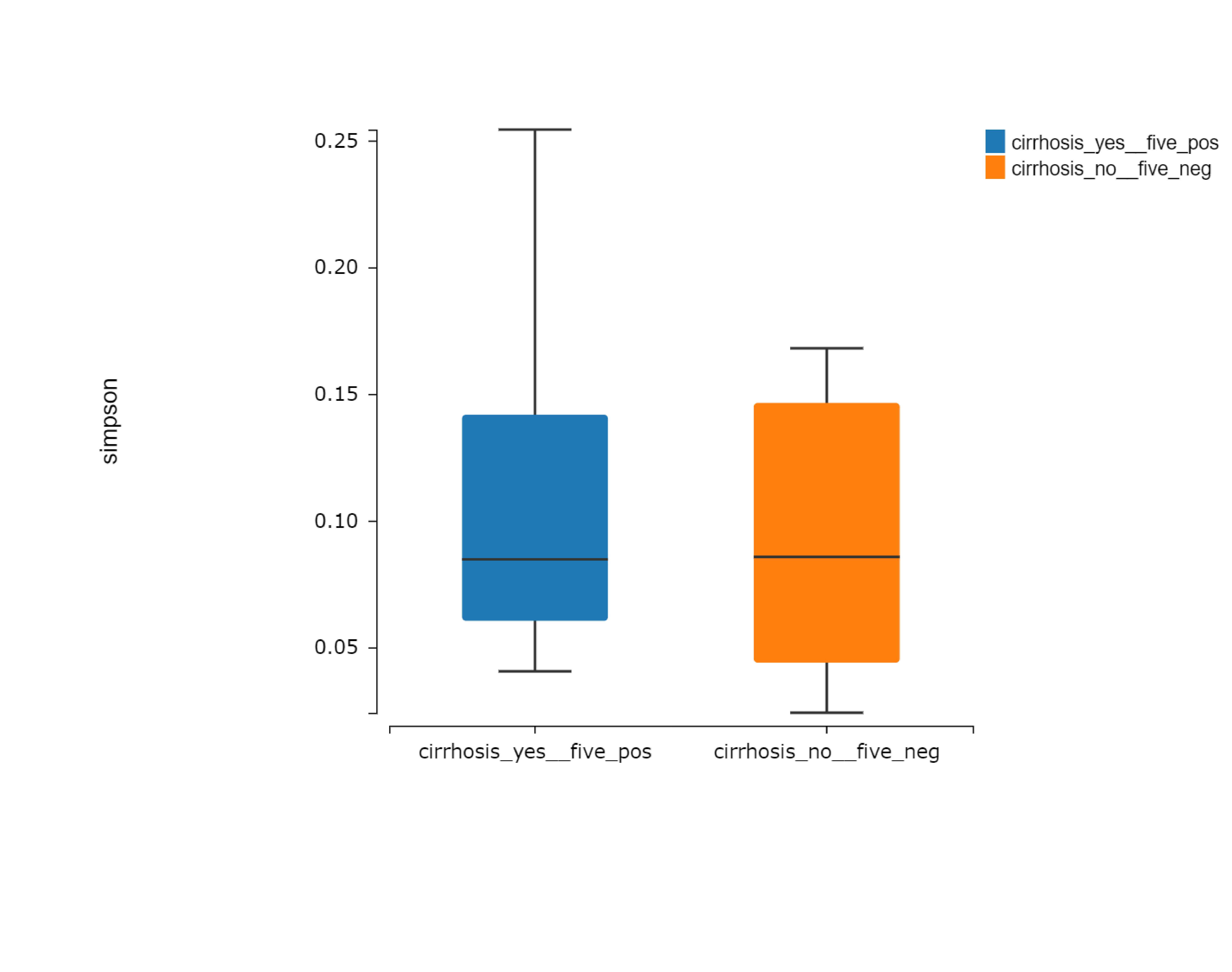

Supplement: Supplementary file 2 [file DataSheet_2.zip › Figure2-5/Figure5/Figure5B simpson.jpg]

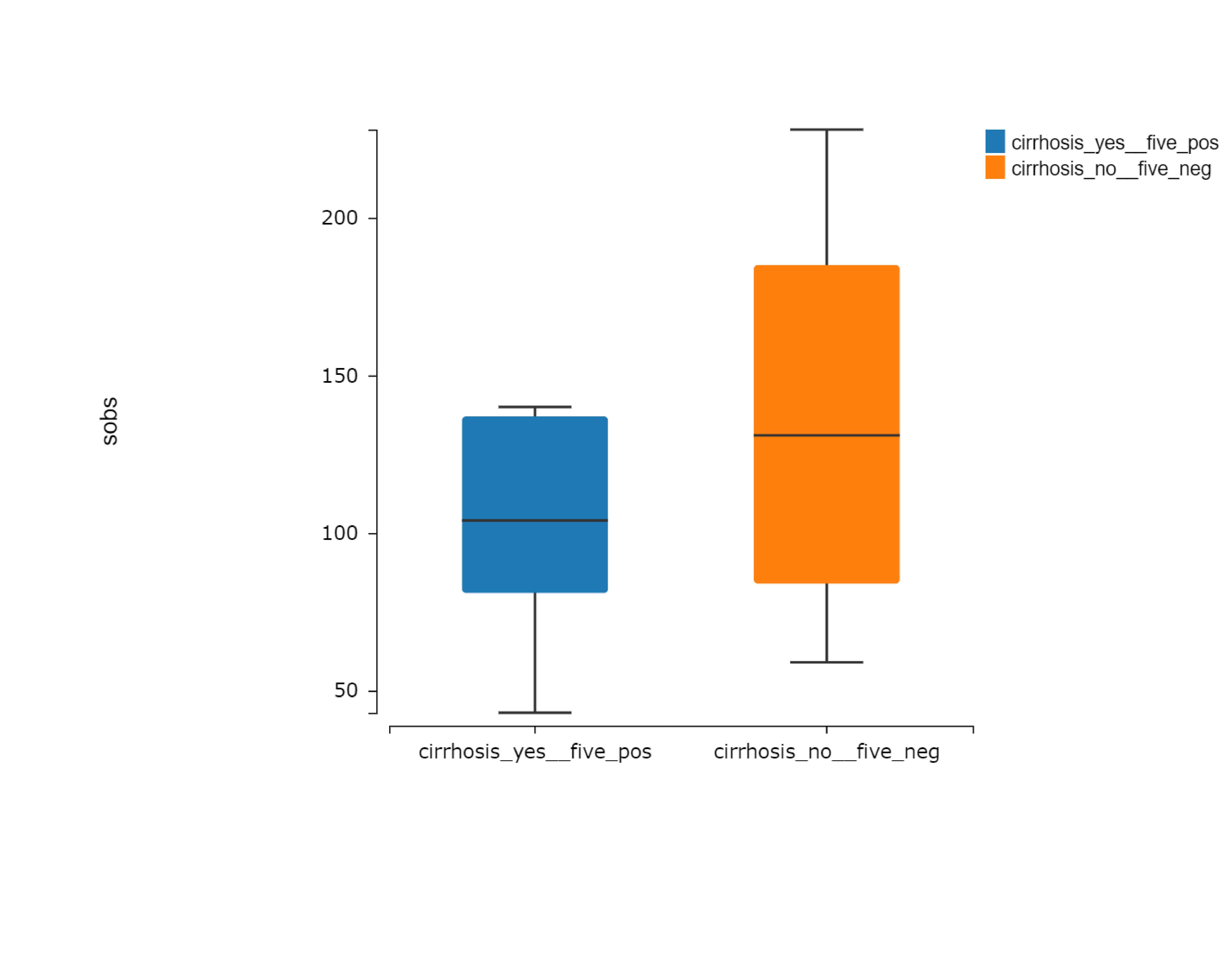

Supplement: Supplementary file 2 [file DataSheet_2.zip › Figure2-5/Figure5/Figure5B sobs.jpg]

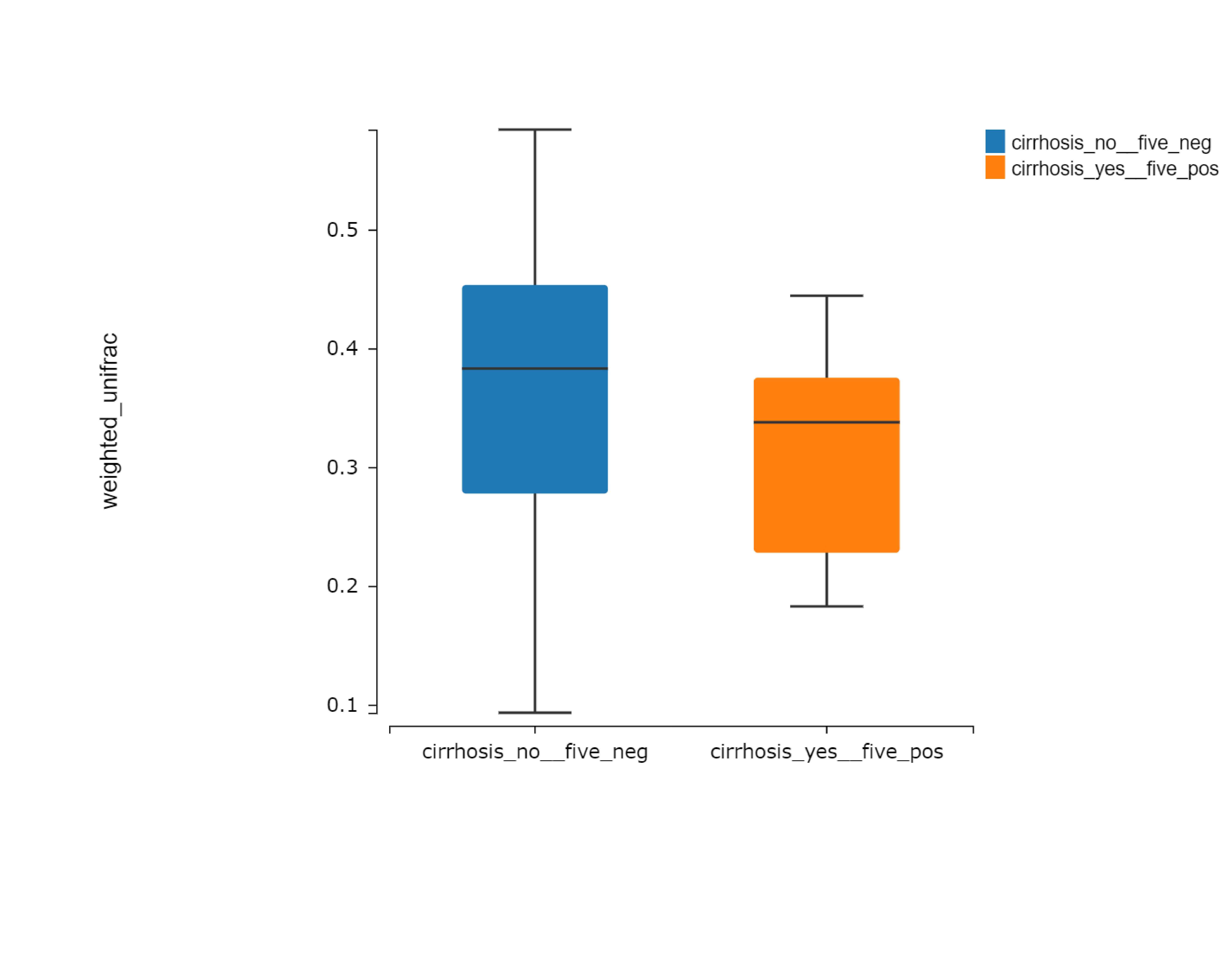

Supplement: Supplementary file 2 [file DataSheet_2.zip › Figure2-5/Figure5/Figure5C .jpg]

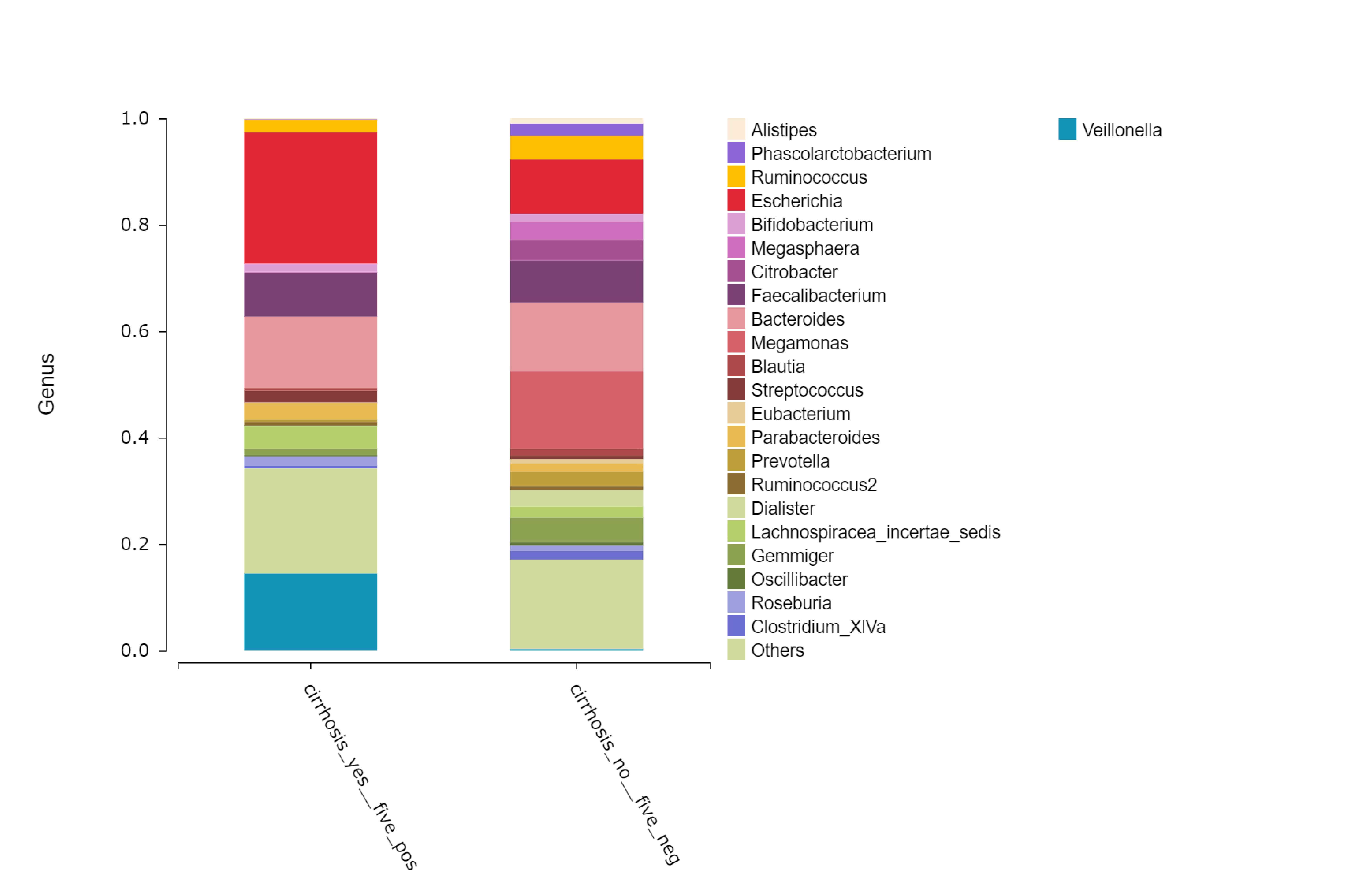

Supplement: Supplementary file 2 [file DataSheet_2.zip › Figure2-5/Figure5/Figure5D .jpg]

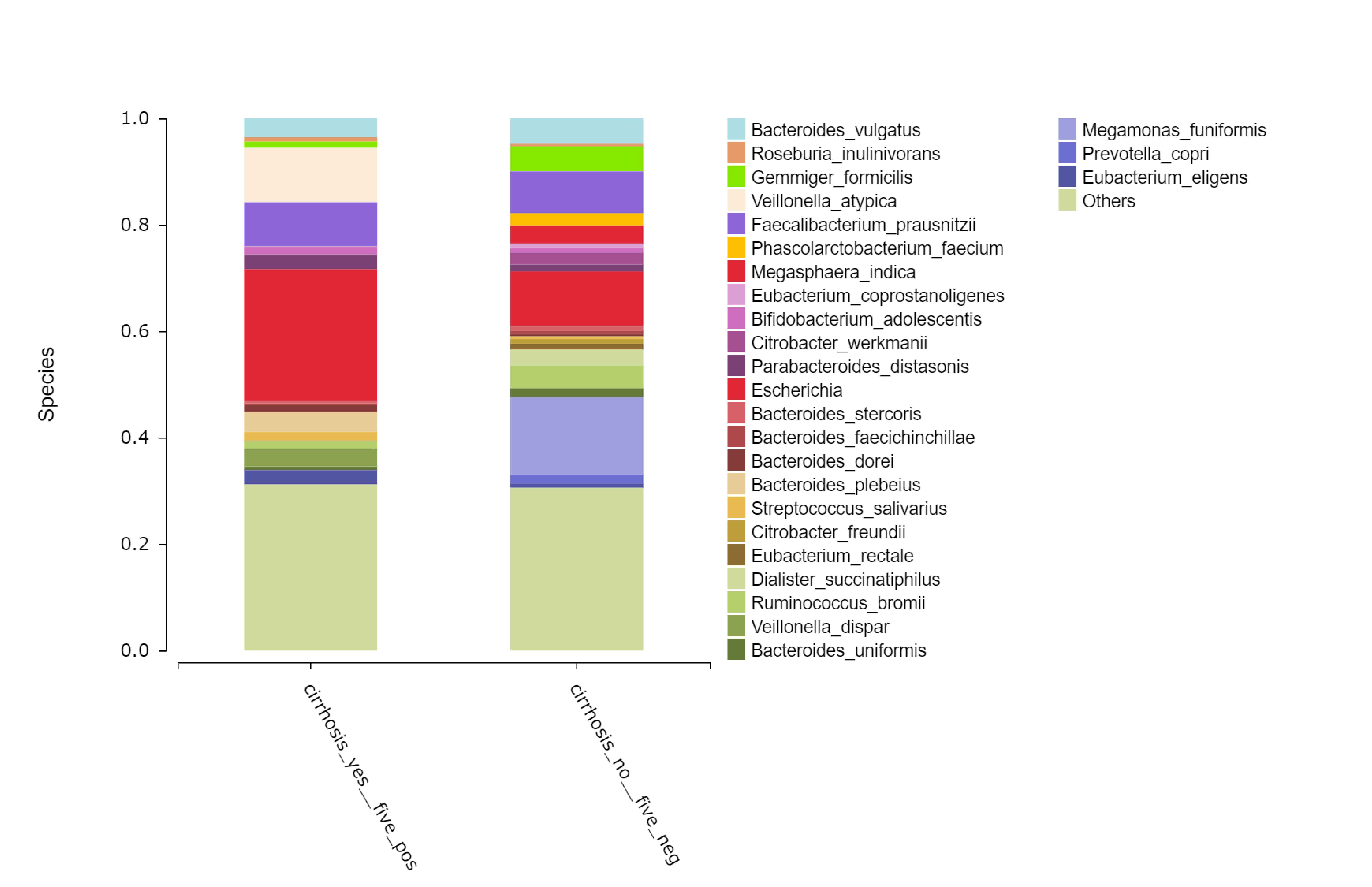

Supplement: Supplementary file 2 [file DataSheet_2.zip › Figure2-5/Figure5/Figure5D-1 .jpg]

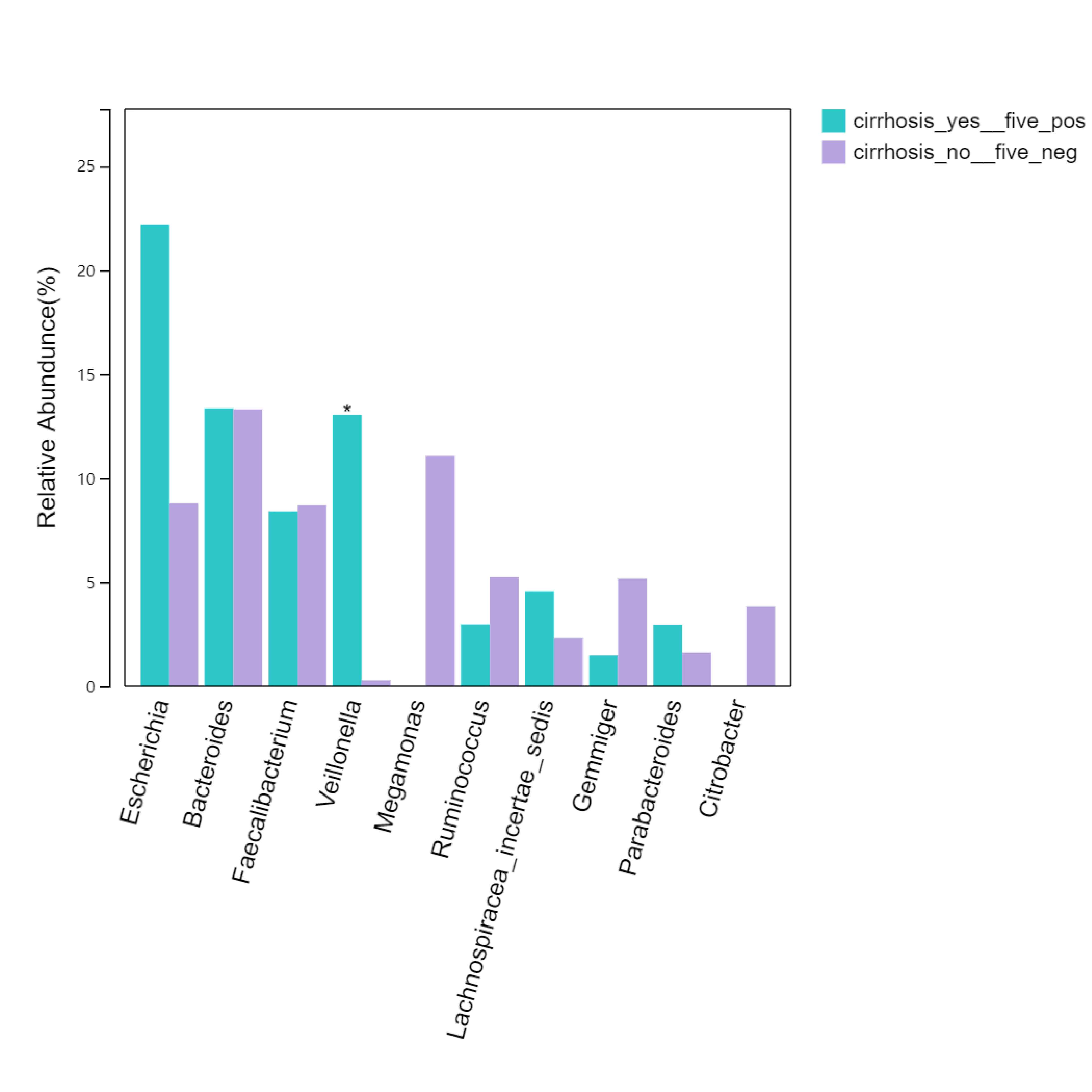

Supplement: Supplementary file 2 [file DataSheet_2.zip › Figure2-5/Figure5/Figure5E .jpg]

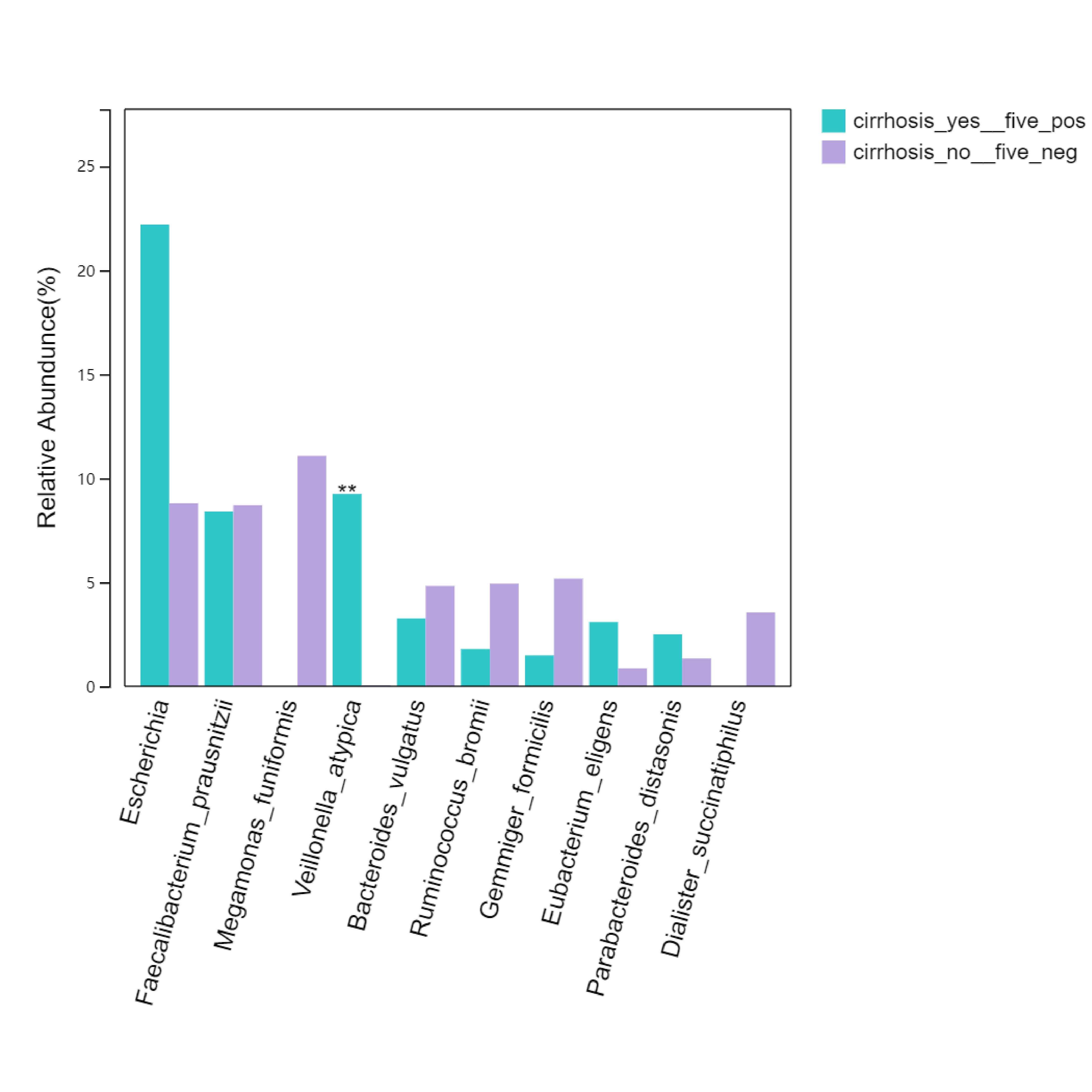

Supplement: Supplementary file 2 [file DataSheet_2.zip › Figure2-5/Figure5/Figure5E-1 .jpg]

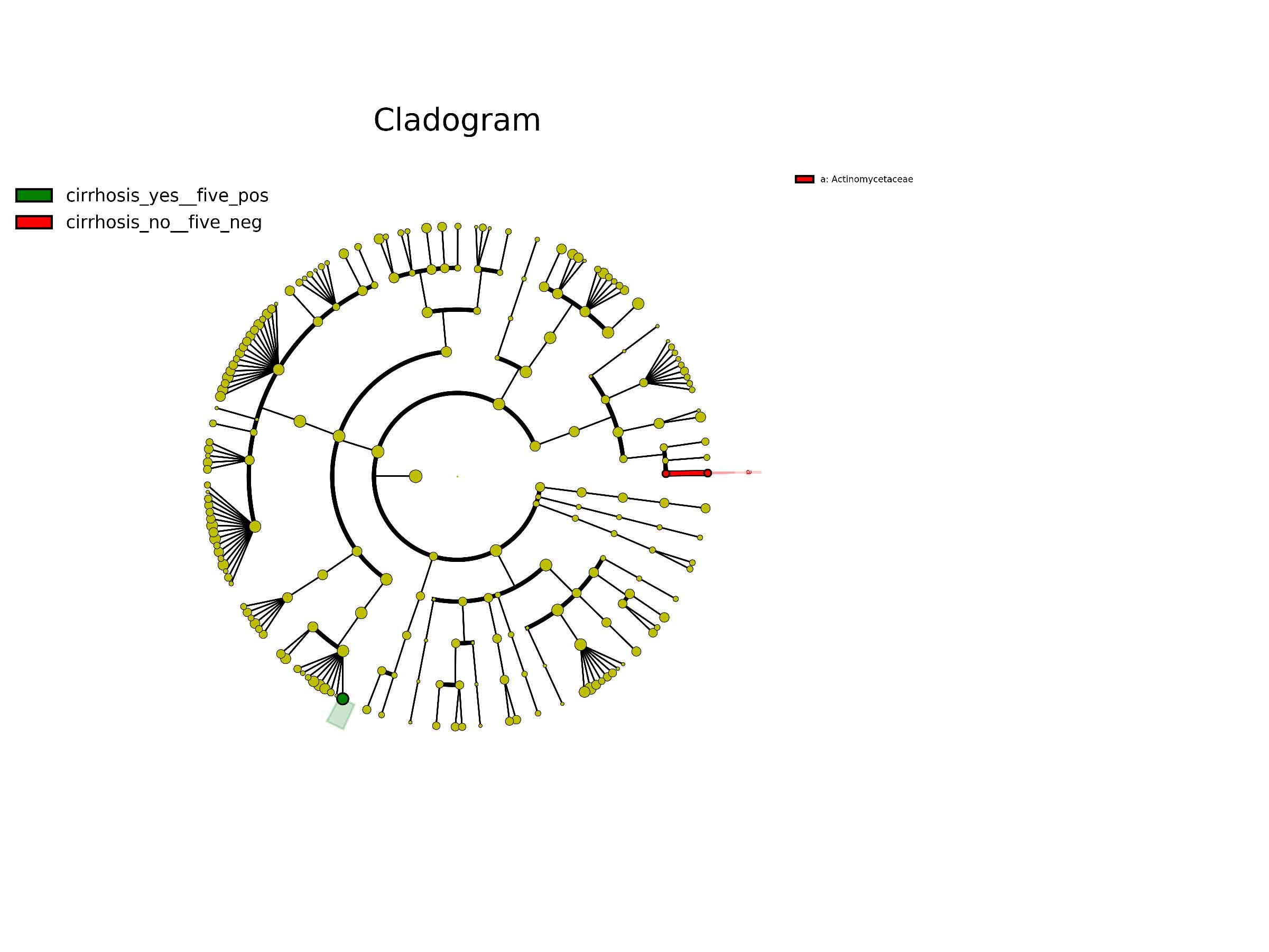

Supplement: Supplementary file 2 [file DataSheet_2.zip › Figure2-5/Figure5/Figure5F .jpg]

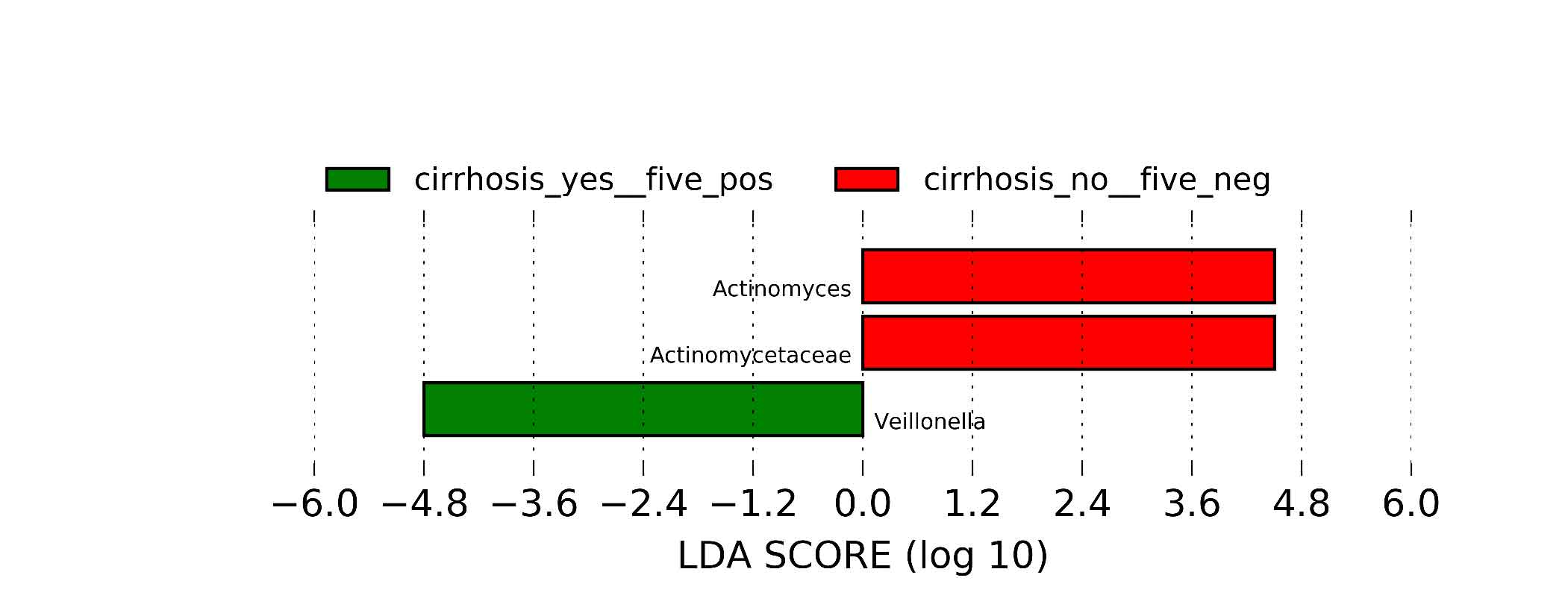

Supplement: Supplementary file 2 [file DataSheet_2.zip › Figure2-5/Figure5/Figure5G .jpg]

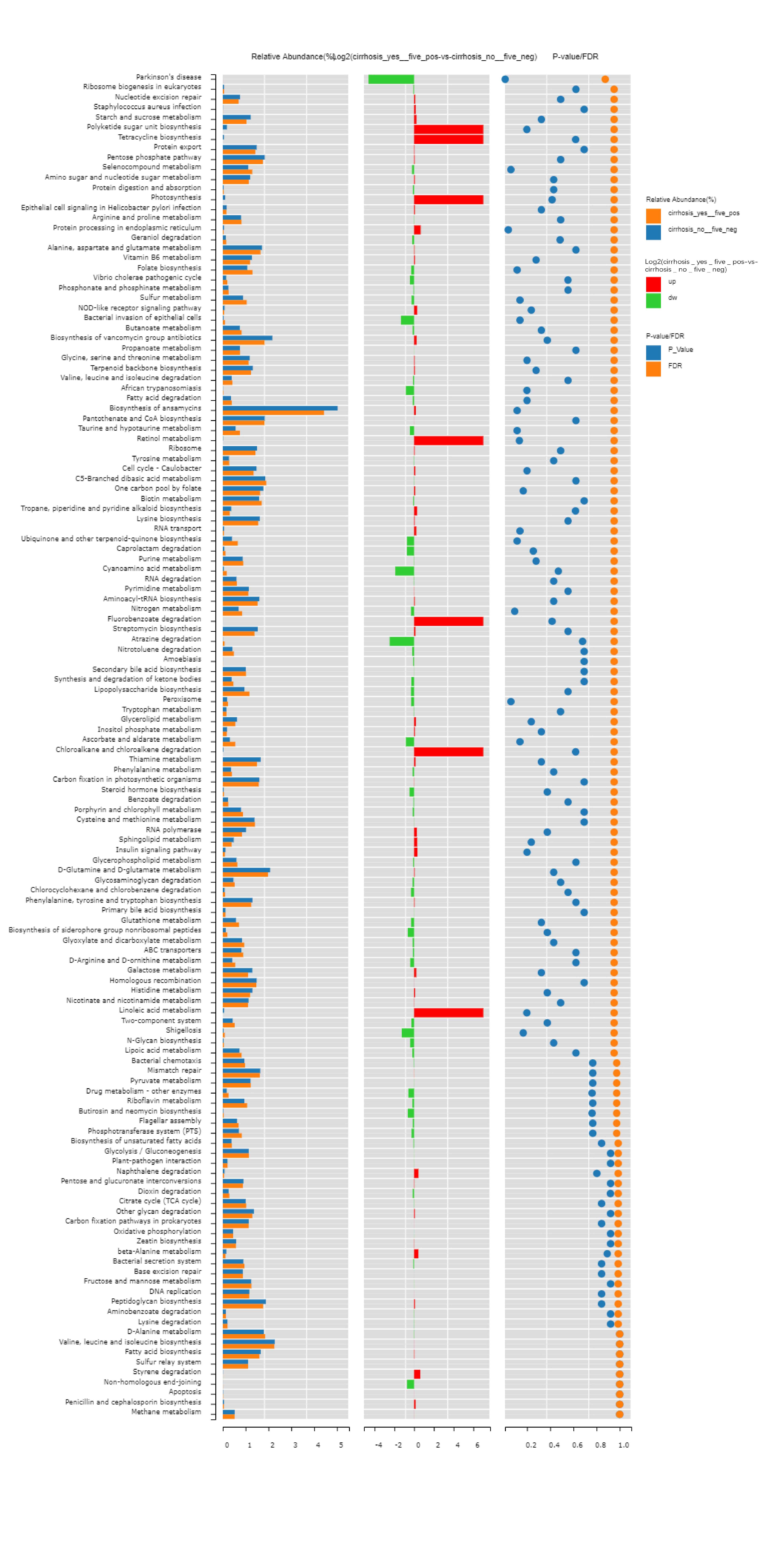

Supplement: Supplementary file 2 [file DataSheet_2.zip › Figure2-5/Figure5/Figure5H.jpg]
